# Multi-omic analysis of jaw-bone periosteum identifies the role of macrophage-derived SPP1 in *Ctsk*^*+*^*Fmod*^*+*^ periosteal cells

**DOI:** 10.1038/s41413-026-00585-7

**Published:** 2026-09-22

**Authors:** Zumu Yi, Yeyu Liu, Jing Wang, Chen Deng, Yixin Jin, Yili Qu, Chen Hu, Yi Man

**Affiliations:** 1 State Key Laboratory of Oral Diseases & National Center for Stomatology & National Clinical Research Center for Oral Diseases & Department of Oral Implantology, West China Hospital of Stomatology, Sichuan University, Chengdu, Sichuan China; 2 Department of Oral Implantology, The Affiliated Hospital of Stomatology, Kunming Medical University, Kunming, Yunnan China; 3 State Key Laboratory of Oral Diseases & National Center for Stomatology & National Clinical Research Center for Oral Diseases & Department of Prosthodontics, West China Hospital of Stomatology, Sichuan University, Chengdu, Sichuan China

**Keywords:** Bone, Bone quality and biomechanics

## Abstract

Periosteal cells (PCs) play a crucial role in bone regeneration. Currently, the spatial landscapes of jaw-bone periosteum during bone grafting remains unclear. In this study, we propose a modified guided bone regeneration (GBR) model and integrate single-cell RNA sequencing (scRNA-seq) and spatial transcriptomics (ST) to dissect its spatial characteristics. We identify a functional subset of *Ctsk*^*+*^*Fmod*^*+*^ PCs within the jaw-bone periosteum of rat and human. In addition, we describe a spatial niche around the bone graft substitute, characterized by massive infiltration of macrophages interacting with *Ctsk*^*+*^*Fmod*^*+*^ PCs. Notably, SPP1 secreted by macrophages attenuates the osteogenic capacity of *Ctsk*^*+*^*Fmod*^*+*^ PCs. The discovery of this *Ctsk*^*+*^*Fmod*^*+*^ subcluster, characterized by osteoprogenitor features and close interactions with macrophages, sheds light on the composition of the subperiosteal microenvironment and the heterogeneity of jaw-bone PCs.

## Introduction

The periosteum, an intricate and well-organized connective tissue envelope with abundant blood supply, covers the surface of most bones.^1^ Originating from the perichondrium, the mature periosteum is commonly categorized into inner and outer layers.^2^ Within this tissue, periosteal cells (PCs) display notable heterogeneity, comprising diverse cellular populations, ranging from periosteal stem cells (PSCs) to myeloid lineage cells (MCs), osteogenic cells and fibroblasts.^3–6^ PSCs play a crucial role in bone development and maintenance, as they collaboratively coordinate the intramembranous and endochondral ossification.^7^

Due to the cellular heterogeneity and functional variability of PCs, identifying these progenitor cells remains challenging. Studies have leveraged genetic lineage tracing to identify distinct pools of lineage-restricted embryonic and adult periosteal progenitors.^8^ Cathepsin K-lineage PCs highlight a periosteal population (*Ctsk*-GFP^*+*^*CD49*f^low^*CD51*^low^: *CD200*^*+*^*CD105*^−^) that play a role in bone regeneration through both endochondral ossification during fracture repair and intramembranous ossification at steady state.^9^ *Mx1-αSMA* co-labeled cells also label a subset of PCs, which has high colony formation potential and is crucial for injury repair.^10^ *Sox9*-lineage progenitors in the periosteum initiate cartilage callus formation by giving rise to skeletal cells.^11,12^ Noteworthy markers such as platelet-derived growth factor receptor α (*PDGFRα*), *Grem1*, *Gli1*, and *Nestin* (*Nes*) have also been employed in tracing different PCs subpopulation.^13–15^ The periosteum exhibits incredible diversity in populations, and distinct periosteal cell populations may exist in different regions.^6,16^ This recognition emphasizes the intricate nature of the periosteum and underscores the need for a comprehensive characterization of periosteal cell heterogeneity.

Accumulating evidence reveals distinctive biological characteristics exhibited by PCs within the jaw bones, attributed to variances in developmental, mechanical, and homeostatic properties when compared to long bones.^17–19^ Mandible PCs show enhanced osteogenic and angiogenic potential in comparison to tibia PCs.^20^ PCs in the jaw are equally vital to the formation and development of jaw bones. Clinically, healing of segmental jawbone defects occurs through endochondral ossification, where PCs play a pivotal role in the development of the cartilaginous callus.^12,21^ The local ablation of a unique *Ctsk-Ly6a* co-labeled subset of jaw bone *Ctsk*-lineage cells in periosteum has been reported to impede fracture repair.^17^ Nonetheless, studies elucidating the properties of PCs within the jaw bones are still scarce, leaving the functional and phenotypic characteristics of these cells largely unresolved.

Moreover, the immune microenvironment exhibits elevated activity within the alveolar bone, with macrophages demonstrating particularly robust interactions with periosteum-derived cells. Tartrate-resistant acid phosphatase-positive (*TRAP*^*+*^) macrophages, for instance, can recruit Nes^*+*^ and leptin receptor^*+*^ periosteum-derived cells for bone formation by secreting platelet-derived growth factor-BB (PDGF-BB).^22^ CD68^*+*^F4/80^*+*^ macrophages localized in the periosteum express and activate transforming growth factor β (TGF-β1) to facilitate the recruitment of PCs.^3^ Additionally, macrophage-derived SPP1 has been implicated in the regulation of stromal progenitor differentiation.^23^ *Spp1*^hi^ macrophages have been reported to significantly contribute to fibrotic processes across diverse tissues and organs.^23–26^ Nevertheless, the specific impact of these macrophages on jaw-bone PCs remains to be explored.

Single-cell RNA sequencing (scRNA-seq) studies of periosteum revealed the composition of periosteal cells and facilitated the identification of key effector cells involved in various physiological contexts.^27,28^ However, the disruption of the original tissue structure precludes the ability to discern alterations in the spatial organization of cells during periosteal activation. As the function of many biological systems depends on the spatial organization of their cells,^29^ it is essential to further investigate the spatial dynamic changes of the jaw-bone periosteum involved in osteogenesis using spatial transcriptomics (ST).

In this study, we propose a modified guided bone regeneration (GBR) model and integrate high-throughput scRNA-seq and ST to delineate the spatial characteristics of jaw-bone periosteum. Our findings clarify the presence of a functional subset of PCs within the jaw bones characterized by *Ctsk*^*+*^*Fmod*^*+*^. After the bone graft substitute is implanted, *Ctsk*^*+*^*Fmod*^*+*^ PCs were activated, contributing to the formation of intricate ecological niches alongside macrophage subpopulations around the bone graft substitute. Notably, SPP1 secreted by macrophages impair the osteogenic potential of *Ctsk*^*+*^*Fmod*^*+*^ PCs. Blocking of SPP1 reveals a significant enhancement in the osteogenic capacity of *Ctsk*^*+*^*Fmod*^*+*^ PCs, thereby fostering osteogenesis within the jaw-bone subperiosteal microenvironment.

## Results

### The establishment of modified guided bone regeneration (GBR) model and analysis of the surrounding microenvironment

Clinicians have modified GBR procedures by using the intact periosteum to cover implanted bone graft substitute because of the regenerative potential of the periosteum.^30,31^ We evaluated bone formation following the modified subperiosteal GBR procedures and compared the clinical outcome with conventional GBR.^32,33^ Histological analysis of tissues obtained 6 months post-surgery indicated the presence of newly formed bone beneath the periosteum (Fig. S1a–c). Additionally, cone-beam computed tomography (CBCT) and X-ray images demonstrated that the modified GBR technique, which preserved the integrity of the periosteum, led to acceptable horizontal bone augmentation (Fig. S1d, e).

**Fig. 1:**
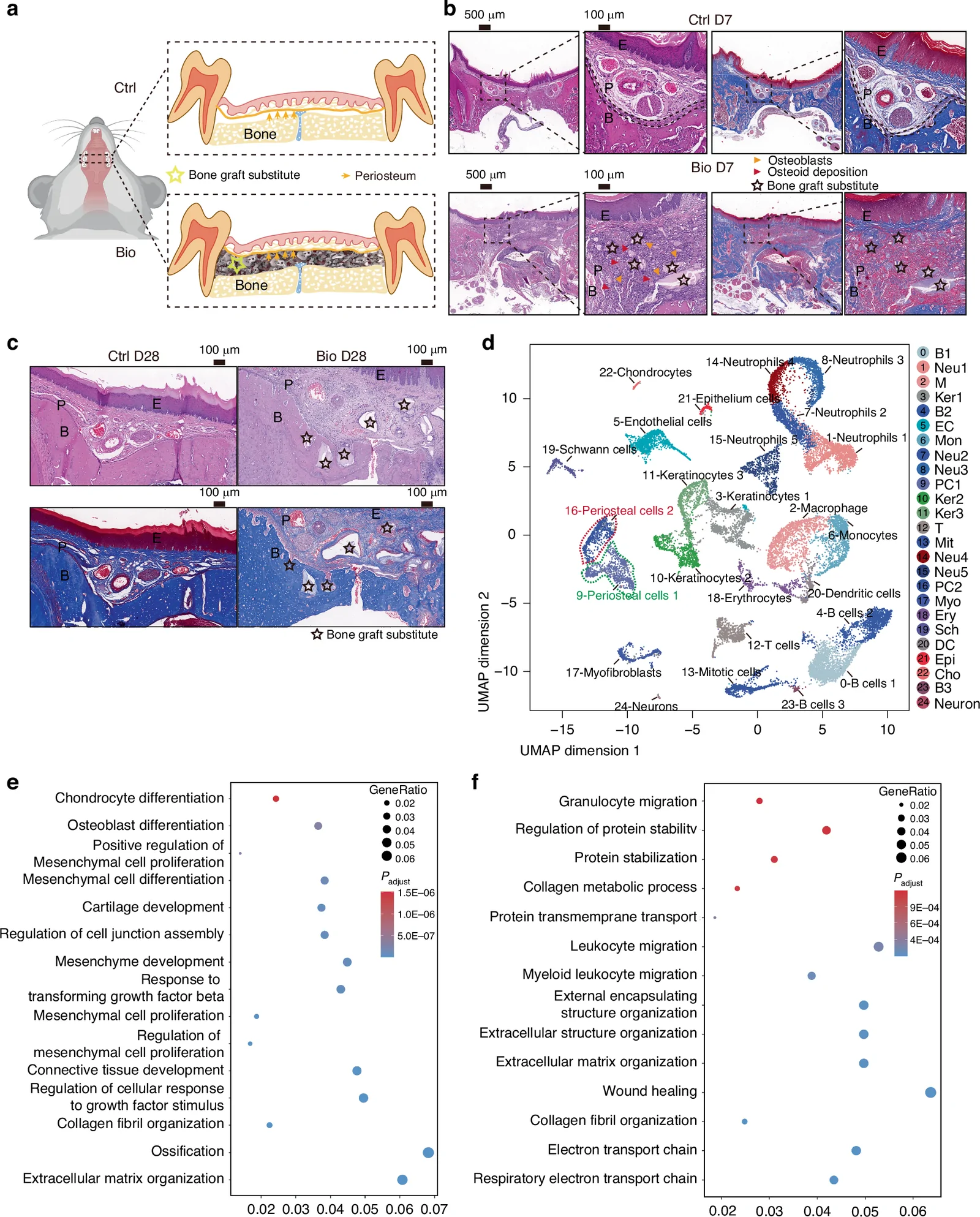
The establishment of a rat subperiosteal bone grafting model and single-cell landscape of Bio and Ctrl group at different timepoints. **a** Schematic diagram of rat subperiosteal bone grafting model. The animal model involves complete periosteum elevation followed by bone substitute implantation beneath the intact periosteal layer like the clinical procedure. Stars, bone graft substitute. Yellow arrows, periosteum. Representative H&E and Masson staining images of Ctrl and Bio group on day 7 (**b**) and 28 (**c**). Black dotted line, periosteum; Yellow arrows, osteoblasts; Red arrows, osteoid deposition; Stars, bone graft substitute. **d** Uniform Manifold Approximation and Projection (UMAP) plots of 19 377 cells from different groups, showing the cell number of 25 clusters. The green circle indicates periosteal cells 1 and the red circle indicates periosteal cells 2. B-B cells, Neu-Neutrophils, M-Macrophages, Ker-Keratinocytes, EC-Endothelial cells, Mon-Monocytes, PC-periosteal cells, T-T cells, Mit-Mitotic cells, Myo-Myofibroblasts, Ery-Erythrocytes, Sch-Schwann cells, DC-Dendritic cells, Epi-Epithelium cells, Cho-Chondrocytes, Neuron-Neurons. Differentially expressed genes (DEGs) of PCs (PC1 & PC2) between different groups on day 3 (**e**) and 7 (**f**)

The workflow of our study is shown in Fig. S2a. Inspired by the modified GBR procedures in clinical practice,^32–34^ we established a modified GBR model in the rat jaw bone (Fig. 1a). The group that received the bone graft substitute (Bio-Collagen) was labeled as the Bio group, while the group undergoing sham surgery was referred to as the Ctrl group. We measured the volume of each bone graft substitute to ensure that the implantation volume was the same for each animal (Fig. S2b, c). To ensure the integrity of the elevated periosteum, we assessed its structure post-elevation. In the untreated group, the periosteum remained attached to the hard palate, while in the operated group, it was completely elevated from the bony surface (Fig. S3a).

Subsequently, we examined whether the subperiosteal bone grafting model could promote de novo bone formation. Samples were harvested at day 3, 7, 14, and 28 post-operation. Accumulated red blood cells and fibrin networks were observed in the Bio group on day 3 (Fig. S3b). By postoperative day 7, osteoblasts and osteoid deposition were present near the bone graft substitute (Fig. 1b). The results of immunohistochemical staining for osterix also showed that there were *osterix*^*+*^ osteoblast cells around the bone graft substitute in the Bio group on day 7 (Fig. S3c). Concurrently, the periosteum underwent remodeling and lost its original structure. Immunofluorescence staining indicated the expression of osteogenesis-related markers around the bone graft substitute (Fig. S3f). New bone formation was notable by day 14, with pronounced newly formed bone in contact with the bone graft substitute by day 28 (Figs. 1c and Fig. S3b). In contrast, the control group exhibited periosteal thickening post-surgery (compared with no control (NC) group, Fig. S3d, e). Although the control group showed activation of the periosteum, there was no significant new bone formation, which may be related to the lack of osteogenic space. Taken together, these findings showed that the implantation of bone graft substitute under the periosteum could induce bone regeneration in the jaw bones, thereby confirming the successful establishment of the modified GBR model.

To elucidate the cellular composition within jaw-bone periosteum, three fresh samples were harvested from the surgical site per group (Bio and Ctrl) at different time points (day 3 and day 7) (Fig. S4a). We processed these samples into single-cell suspensions and subjected them with scRNA-seq profiling. Following quality control, our dataset contained 19 377 cells. Canonical correlation analysis (CCA) function of Seurat was used to correct the batch effect^35^ (Fig. S4b). We obtained 25 clusters and annotated each cluster with its respective markers (Figs. 1d and S4c). The identified marker genes for periosteal cell (PC) (*Ctsk*, *Postn*, *Prrx1,* and *Pdgfrα*), macrophage (*Cd68* and *Cd14*) and monocyte-macrophage (*Fcnb* and *Cd14*) were cell type specific (Fig. S4d, e).

A comparative analysis of cell proportions revealed an expansion of the periosteal cell populations (PC1 and PC2) on day 3 post-implantation. However, this difference was no longer significant by day 7. Meanwhile, the frequency of macrophages was notably higher in the Bio group at both time points (Fig. S4f–h).

We further compared the differentially expressed genes (DEGs) of PCs (PC1 & PC2) between two groups. On day 3, genes associated with osteogenesis-related activities, such as ossification and cartilage development, were highly expressed in the Bio group (Fig. 1e). This suggests that PCs were activated and engaged in osteogenesis-related activities upon implantation of the bone graft substitute under the periosteum. Previous studies showing that periosteal tissues respond rapidly to injury or mechanical stimulation, with early initiation of osteogenic differentiation programs.^36,37^ However, by day 7, genes associated with immune-related functions were up-regulated (Fig. 1f), which is consistent with the increased proportions of immune cell populations such as Neu1, Neu2, and M (Fig. S4g, h). Inflammation signals detected at day 7 likely reflect the formation of a material-associated inflammatory niche around the bone graft substitute. Biomaterial implantation elicits a foreign body response involving acute inflammation and monocyte or macrophage recruitment.^38,39^

### Heterogeneity analysis of PCs and identification of jaw-bone *Ctsk*^*+*^*Fmod*^*+*^ PCs

**Fig. 2:**
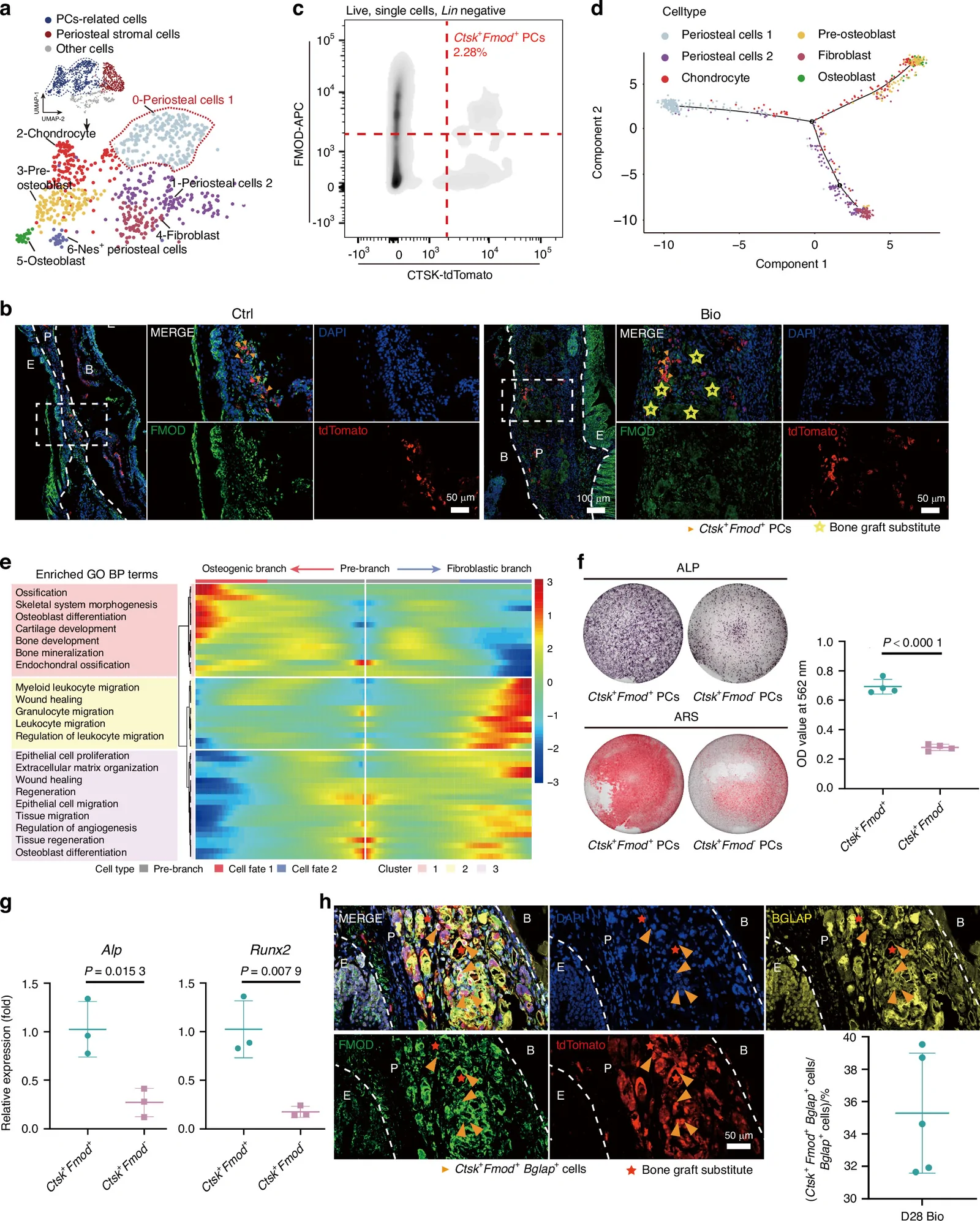
Periosteal cell identification and characterization in jaw bone. **a** UMAP plots showing subclustering results of PCs. The red circle indicates periosteal cells 1. **b** Representative confocal images of sample sections from Ctrl and Bio group. The periosteum contained *Ctsk*^*+*^*Fmod*^*+*^ PCs. Yellow arrows, *Ctsk*^*+*^*Fmod*^*+*^ PCs. Stars, bone graft substitute. E epithelium, P periosteum, B bone. **c** Schematic representation of the strategy used for Flow Cytometry analysis of *Ctsk*^*+*^*Fmod*^*+*^ PCs. **d** Trajectory reconstruction of PCs based on a pseudo-temporal order from left to right; color-coding by celltype. **e** Gene expression heatmap of branch 2 in a pseudo-temporal order. Osteogenic and Fibroblastic trajectories are shown on the left and right, respectively. And enriched GO BP terms of each cluster were shown. **f** ALP staining and ARS staining of *Ctsk*^*+*^*Fmod*^*+*^ PCs and *Ctsk*^*+*^*Fmod*^*−*^ PCs on day 7. **g** qPCR analysis of the mRNA levels of *Alp* and *Runx2* of *Ctsk*^*+*^*Fmod*^*+*^ PCs and *Ctsk*^*+*^*Fmod*^*−*^ PCs. **h** Representative confocal images of sample sections from Bio group at day 28. The periosteum contained *Ctsk*^*+*^*Fmod*^*+*^*Bglap*^*+*^ cells (an average of 35.29% of the total *Bglap*^*+*^ cells). Yellow arrows, *Ctsk*^*+*^*Fmod*^*+*^*Bglap*^*+*^ cells. Stars, bone graft substitute. E epithelium, P periosteum, B bone

We extracted cells defined as PCs and subdivided them into 12 clusters. Based on previously published marker genes, we defined PC-related cell populations, including periosteal cell 1 (*Ctsk*, *Fmod*, *Tnmd* and *Prrx2*), periosteal cell 2 (*Prrx1*, *Col8a1* and *Pdgfrα*), chondrocyte (*Sox6* and *Ucma*), pre-osteoblast (*Alpl* and *Ostn*), fibroblast (*Acta2* and *Tagln*), osteoblast (*Alpl* and *Bglap*) and *Nes*^*+*^ periosteal cell (*Nes* and *Dlx5*) populations^9,13,15,40^ (Figs. 2a and S5a,b). Additionally, a subset of periosteal stromal cells containing fibroblasts/stromal cells (*Aoc3*, *Gsn* and *Igfbp6*) and *Ecm1*^*+*^ fibroblasts (*Ecm1*, *Angptl1* and *Col3a1*) was identified (Fig. S5c).

To clarify the function of each subclusters, we obtained the top marker genes for each cluster and conducted GO analysis. GO analysis unveiled that the periosteal cell 1 subcluster was associated with ossification and osteoblast differentiation (Fig. S5d). Periosteal stromal cells were primarily associated with extracellular matrix and structural organization (Fig. S5e). The above results indicated that periosteal cell 1 (*Ctsk*^*+*^*Fmod*^*+*^ PCs) might contribute to the bone formation process.

To validate the presence and localization of *Ctsk*^*+*^*Fmod*^*+*^ PCs in jaw bones, we used *CtskCre*; *Rosa26-LSL-tdTomato* mice to trace the *Ctsk*^*+*^*Fmod*^*+*^ PCs in different groups. Consistent with the scRNA-seq results, a distinct population of *Ctsk*^*+*^*Fmod*^*+*^ cells were identified in the periosteum of jaw bones (Fig. 2b). In the Bio group, *Ctsk*^*+*^*Fmod*^*+*^ cells were present around the bone graft substitute (Fig. 2b). Since *Ctsk* may also mark osteoclasts, we performed TRAP staining to evaluate the spatial locations of *tdTomato*^*+*^ cells and *Trap*^*+*^ cells. The results showed that a small number of *Trap*^*+*^ cells appeared around the bone graft substitute, but they did not overlap with *tdTomato*^*+*^ cells (Fig. S5f). Flow cytometric analysis with fixation and permeabilization further confirmed the existence of the *Ctsk*^*+*^*Fmod*^*+*^ cell population (Figs. 2c and S6). We also analyzed changes in the proportions of different cell subsets. The number of cells in all periosteal subsets (except for *Nes*^*+*^ periosteal cells) was higher in the Bio group on day 3 (Fig. S5g). On day 7, a higher proportion of fibroblasts and pre-osteoblasts were observed in the Bio group (Fig. S5g). Previous studies have shown that Ctsk-lineage periosteal cells consist of three populations: 1) *CD105*^*−*^*CD200*^*+*^ bona fide periosteal stem cells (PSCs), 2) *CD105*^*−*^*CD200*^−^ non-stem periosteal progenitors 1 (PP1), 3) *CD105*^*+*^*CD200*^variable^ non-stem periosteal progenitors 2 (PP2).^8,9^ In this study, we found that the expression patterns of *Cd200* and *Cd105* in the *Ctsk*^*+*^*Fmod*^*+*^ PCs were different from those in long bone periosteal cells (Fig. S5h, i). Consequently, *CD200* and *CD105* are not entirely suitable for phenotypic generalization of progenitor cells in the context of the jawbone modified GBR model.

Since periosteal activation was a confounding factor in our experimental models (including both Ctrl and Bio groups), we investigated whether *Ctsk*^*+*^*Fmod*^*+*^ PCs exist as a resident population under unperturbed physiological conditions. We leveraged public scRNA-seq datasets from murine,^41^ human,^42^ and porcine^43^ cranial periosteum to characterize the steady-state cellular landscape. Our cross-species analysis identified a distinct cluster of *Ctsk*^*+*^*Fmod*^*+*^ PCs present across all examined datasets (Fig. S7a, b, d, e, g, h). GO enrichment analysis revealed that this population is significantly enriched in pathways related to ossification and osteoblast differentiation, exhibiting a transcriptional profile highly concordant with the *Ctsk*^*+*^*Fmod*^*+*^ PCs identified in our rat jaw bone periosteum data (Fig. S7c, f, i). Furthermore, lineage tracing in *CtskCre*; *Rosa26-LSL-tdTomato* mice validated the presence of these cells in situ under homeostasis (Fig. S7j). Taken together, these results establish that *Ctsk*^*+*^*Fmod*^*+*^ PCs are a bona fide resident population of the periosteum rather than a transient subset generated solely in response to injury or activation.

To generate a pseudo-temporal map of the differentiation trajectories of PC subtypes, we used Monocle2, an algorithm to reconstruct biological processes according to transcriptional similarity.^44^ Trajectory analyses illustrated that upstream periosteal cell 1 (*Ctsk*^*+*^*Fmod*^*+*^ PCs) bifurcated into two different branches: fibroblasts and osteoblasts (Figs. 2d and Fig. S8a, b). Expression levels of *Fmod*, *Wif1*, and *Tnmd* (*Ctsk*^*+*^*Fmod*^*+*^ PCs’ markers) were higher at the early transition states (Fig. S8c). As transition states progressed, genes associated with bone formation (such as *Alp*, *Bglap*, and *Ostn*) and fibroblastic genes (like *Acta2* and *Tagln*) began to increase (Fig. S8c, d). Differential expression analysis between branches shed light on gene expression changes along the trajectory. Cells in the osteogenic branch exhibited higher levels of genes related to osteogenesis compared to those in the fibroblast branch. These genes were enriched for GO terms such as “ossification”, “skeletal system morphogenesis”, “osteoblast differentiation” and “bone development” (Fig. 2e). Collectively, these gene expression patterns elucidated the potential role of *Ctsk*^*+*^*Fmod*^*+*^ PCs. When *Ctsk*^*+*^*Fmod*^*+*^ PCs were stimulated, they might differentiate into osteogenic cells, activating genes associated with osteogenesis.

Next, we attempted to isolate the *Ctsk*^*+*^*Fmod*^*+*^ PCs from jaw bones using multi-color flow cytometry (Fig. S9). After obtaining a single-cell suspension, we labeled the cells with antibodies and obtained *Lin*^*−*^*Ctsk*^*+*^*Fmod*^*+*^ and *Lin*^*−*^*Ctsk*^*+*^*Fmod*^*−*^ cells by fluorescence-activated cell sorting (FACS) (Fig. S9). Compared to *Ctsk*^*+*^*Fmod*^*−*^ cells, the *Ctsk*^*+*^*Fmod*^*+*^ cells exhibited better colony-forming ability (Fig. S8e). Under separate lineage-specific induction conditions, *Ctsk*^*+*^*Fmod*^*+*^ cells demonstrated stronger osteogenic and adipogenic capacity in vitro, as evidenced by ALP staining, quantitative analysis of ARS staining and Oil Red O staining (Figs. 2f and S8f). The *Ctsk*^*+*^*Fmod*^*+*^ cells displayed significantly higher expression of osteogenic markers (*Alp* and *Runx2*) and adipogenic markers (*Cebpa*, *Lpl*) (Figs. 2g and S8g). Finally, we used the *CtskCre; Rosa26-LSL-tdTomato* mice to trace the *Ctsk*^*+*^*Fmod*^*+*^ PCs in vivo. Abundant *Ctsk*^*+*^*Fmod*^*+*^*Bglap*^*+*^ cells could be seen around the bone graft substitute. Notably, *Ctsk*^*+*^*Fmod*^*+*^*Bglap*^*+*^ cells accounted for an average of 35.29% (35.29% ± 3.73%) of the total *Bglap*^*+*^ cells, suggesting that a considerable proportion of the cells involved in osteogenesis were derived from *Ctsk*^*+*^*Fmod*^*+*^ PCs (Fig. 2h).

Overall, our data revealed the landscape of PCs in the jaw bones. PCs comprised multiple functionally distinct cell subtypes. Among these, the *Ctsk*^*+*^*Fmod*^*+*^ subset exhibited multipotent differentiation potential. When the periosteum of jaw bone was stimulated by external stimuli (such as bone graft substitute), the *Ctsk*^*+*^*Fmod*^*+*^ PCs contribute to the generation of osteogenic cells, underscoring their pivotal role in bone formation processes.

### Characterization of *Ctsk*^*+*^*Fmod*^*+*^ PCs in human jaw-bone periosteum

**Fig. 3:**
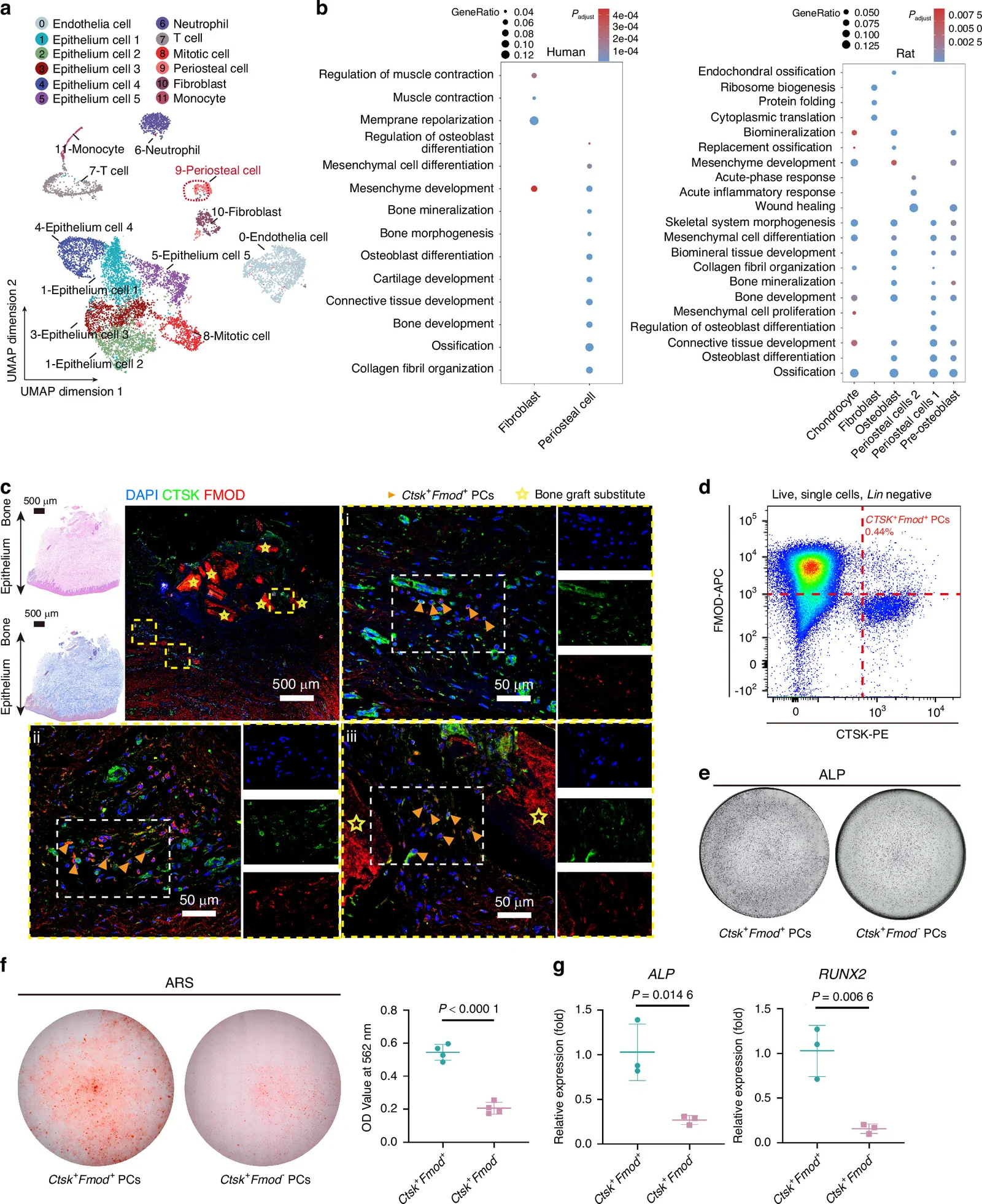
The *Ctsk*^*+*^*Fmod*^*+*^ PCs in human periosteum. **a** UMAP plots showing clustering results of human gingival and periosteal cells. The red circle indicates periosteal cell. **b** GO analysis of subclusters of periosteal cells in human(up) and rat (down). **c** Representative H&E and Masson staining image of human gingiva and periosteum after surgery and confocal images of human periosteum. The human periosteum contained *Ctsk*^*+*^*Fmod*^*+*^ PCs. Stars, bone graft substitute. Yellow arrows, *Ctsk*^*+*^*Fmod*^*+*^ PCs. **d** Schematic representation of the strategy used for FACs analysis of human *Ctsk*^*+*^*Fmod*^*+*^ PCs. **e** ALP staining of *Ctsk*^*+*^*Fmod*^*+*^ PCs and *Ctsk*^*+*^*Fmod*^*−*^ PCs on day 7. **f** ARS staining of *Ctsk*^*+*^*Fmod*^*+*^ PCs and *Ctsk*^*+*^*Fmod*^*−*^ PCs on day 14. **g** qPCR analysis of the mRNA levels of *ALP* and *RUNX2* of *Ctsk*^*+*^*Fmod*^*+*^ PCs and *Ctsk*^*+*^*Fmod*^*−*^ PCs

To verify the presence of *Ctsk*^*+*^*Fmod*^*+*^ PCs in human jaw-bone periosteum, we obtained human gingival and periosteum tissue samples and performed scRNA-seq to analyze the cellular components of human jaw-bone periosteum. Akin to our findings in rats, we identified a subset of cells marked by *Ctsk* and *Fmod* (Figs. 3a and S10a, b). GO analysis showed that this subcluster was related to ossification and mesenchymal cell differentiation, which was consistent with the features of periosteal cell 1 in the rat periosteal cell population (Fig. 3b).

We further acquired tissues from patients undergoing modified GBR procedures. Immunofluorescence staining of sample sections revealed the presence of *Ctsk* and *Fmod* labeled PCs in regions proximal to the bone (Fig. 3c). After dissecting the human periosteum, we isolated human *Lin*^*−*^*Ctsk*^*+*^*Fmod*^*+*^ and *Lin*^*−*^*Ctsk*^*+*^*Fmod*^*−*^ PCs via FACS (Figs. 3d and S10c). We evaluated the growth and differentiation potential of these sorted cells. Similarly, the human *Ctsk*^*+*^*Fmod*^*+*^ PCs showed good colony-forming ability akin to rat (Fig. S10d). Compared to *Ctsk*^*+*^*Fmod*^*−*^ PCs, the *Ctsk*^*+*^*Fmod*^*+*^ PCs showed stronger osteogenic and adipogenic capacity in vitro under separate lineage-specific induction conditions (Fig. 3e–g and S10e, f). These data confirmed the existence of *Ctsk*^*+*^*Fmod*^*+*^ cells in human jaw-bone periosteum and validated their potential for multi-directional differentiation.

### Macrophage infiltration in different groups

Macrophages have been reported to regulate tissue regeneration and closely interact with periosteal cells.^3,45^ We noticed that the Bio group had a higher proportion of macrophages (Fig. S4h). To explore the function of macrophages, we extracted macrophages, monocyte-macrophages and dendritic cells, then clustered them into 9 distinct subpopulations (Fig. S11a). According to known markers,^46–48^ we defined pro-inflammatory macrophages (PIMs) (*Arg1*, *Mmp12* and *Tgm2*), monocytes 1 and 2 (Mono 1 and 2) (*Vcan*, *Cd14* and *Camp*), anti-inflammatory macrophages (AIMs) (*Mrc1*, *Folr1* and *C1qc*), cycling cells 1 and 2 (*Top2a*, *Stmn1* and *Tpx2*), dendritic cells 1 and 2 (DC 1 and 2) (*Cd74* and *Cytip*) and osteoclasts (*Ctsk* and *Slc9b2*) (Fig. S11b).

We then compared the proportions of macrophage subtypes at different times. In the Bio group, the abundance of PIMs and AIMs was greater than that of the Ctrl group, indicative of enhanced recruitment of macrophages upon bone graft substitute implantation (Fig. S11c). It was worth noting that the AIMs were *Cd9*^*+*^*Trem2*^*+*^ macrophages (Fig. S11d). Recent studies have identified *Cd9*^*+*^*Trem2*^*+*^ macrophages expand in fibrotic organs.^49,50^ Furthermore, we assessed the expression of other fibroblast-associated markers (*Spp1*, *Gpnmb*, *Fabp5* and *Cd63*) in macrophage subsets across both groups^25^ (Fig. S11d). Intriguingly, PIMs and AIMs in the Bio group highly expressed these markers, suggestive of populations akin to scar-associated macrophages (SAMs).

Overall, the implantation of bone graft substitutes led to an increased recruitment of PIMs and AIMs to the periosteum, and they exhibited a pro-fibrotic profile in the Bio group.

### Spatial architecture of jaw-bone periosteum

To map the molecular and cellular architecture of PCs and macrophages, we performed ST with tissue sections obtained from different groups on day 3 and 7. Transcriptomics data for 1 152 and 1 033 spots were obtained, with a median depth of 1 694 and 2 417 genes/spot, respectively (Fig. S12a). We adhered to a standardized workflow for the analysis of our sequencing data (Fig. S12b). Histological structural annotation was conducted on HE images, categorizing the regions into keratinized stratified squamous epithelium, connective tissue layer and periosteum, as well as immune cells infiltration area. Dimensionality reduction revealed 6 clusters on day 3 and 9 clusters on day 7 and they were grouped by the morphological structure of the tissues and their markers (Fig. S12c). GO analysis of DEGs in each spatial clusters highlighted enrichment of terms related to osteogenesis in the periosteal region (Fig. S12d).

**Fig. 4:**
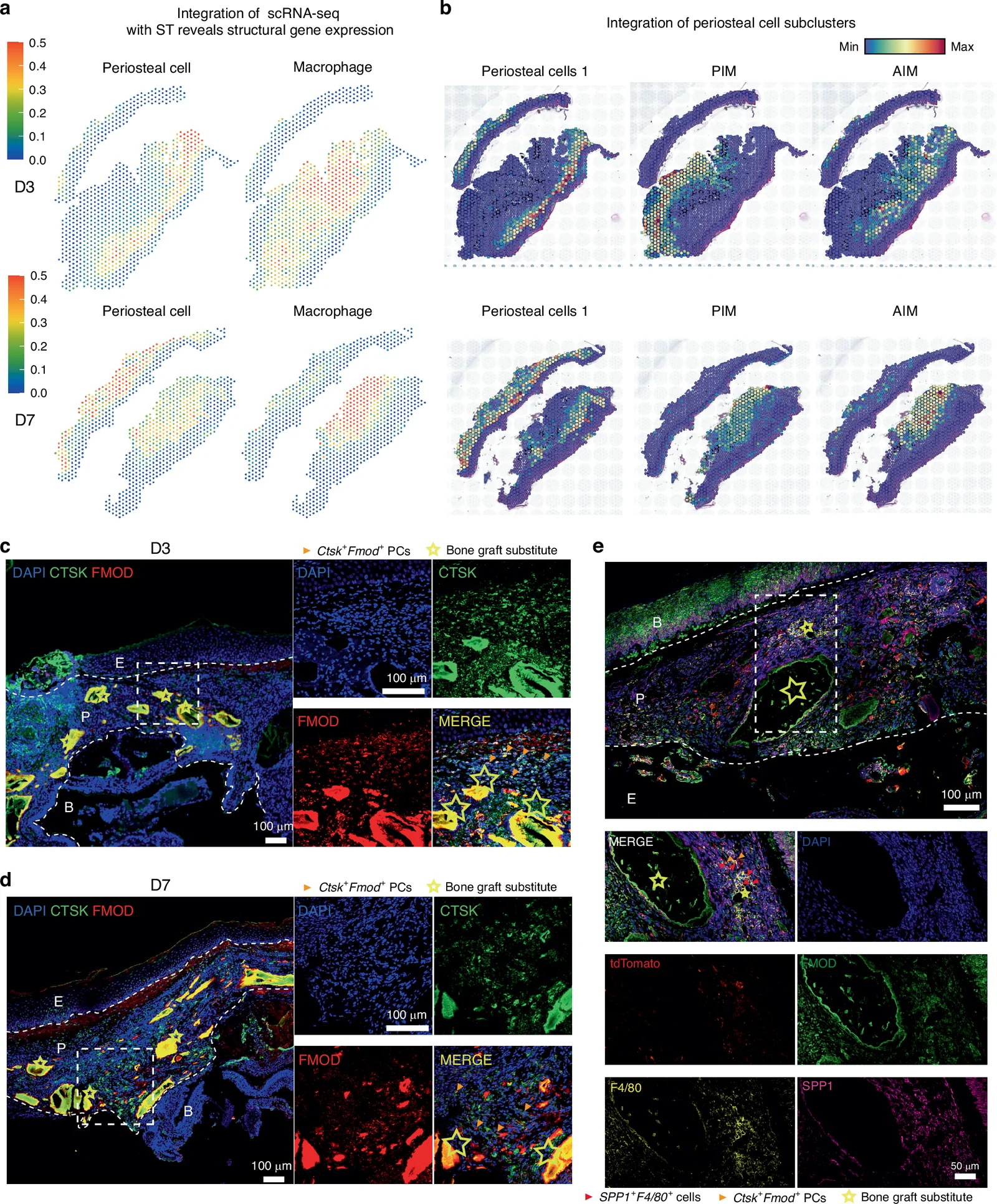
Spatial architecture of jaw-bone periosteum. **a** Spatial mapping of major cell types using the scRNA-seq dataset. **b** Spatial feature plots showing defined subtypes of PCs and macrophages distribution in tissue sections at day 3 and day 7. Representative confocal images of sample sections from day 3 (**c**) and day 7 (**d**) group. The periosteum contained *Ctsk*^*+*^*Fmod*^*+*^ PCs. yellow arrows, *Ctsk*^*+*^*Fmod*^*+*^ PCs. Stars, bone graft substitute. E epithelium, P periosteum, B bone. **e** Representative confocal images of sample sections from day 7 Bio group. Red arrows, *SPP1*^*+*^*F4/80*^*+*^ cells. Yellow arrows, *Ctsk*^*+*^*Fmod*^*+*^ PCs. Stars, bone graft substitute. E epithelium, P periosteum, B bone

To delineate and spatially map major cell types identified in the scRNA-seq dataset, we employed a robust cell type decomposition (RCTD) method.^51,52^ The accuracy of the mapping of periosteum cells and macrophages was confirmed by their spatial localizations and expression of marker genes (Figs. 4a and S12e, f). The migration of PCs around the bone graft substitute on day 7 was consistent with histological findings, and the classical structure of periosteum was lost (Fig. S12c).

Additionally, multimodal intersection analysis (MIA) was utilized to infer enrichment of major cell types.^53–55^ PCs (PC1 & PC2) were predominantly enriched in spatial cluster 0 on day 3 and in spatial cluster 0, 7 and 6 on day 7 (Fig. S12g, h). Notably, macrophages were also enriched in regions containing PCs on day 7, suggesting close spatial proximity between PCs and macrophages (Fig. S12h). Together, these data revealed the structural gene expression of periosteum and defined the spatial co-localization of PCs and macrophages.

Then, ST and scRNA-seq gene expression profiles were integrated using the Addmodulescore function in Seurat^48,56^ to trace the spatial heterogeneity of defined subclusters of PCs and macrophages. We used the top 50 differentially expressed marker genes of subtypes of PCs and macrophages to score spot gene expression profiles. Scoring of spots with periosteal cell 1 (*Ctsk*^*+*^*Fmod*^*+*^ PCs) genes from scRNA-seq showed a cluster highly associated with periosteal cell 1 (*Ctsk*^*+*^*Fmod*^*+*^ PCs) in ST (cluster 0 in day 3 and clusters 0, 5–7 in day 7) (Fig. S13a). This observation aligns closely with the spatial expression pattern of periosteal cell 1 (*Ctsk*^*+*^*Fmod*^*+*^ PCs) markers, thereby validating the accuracy of the spatial mapping (Fig. S13a). We applied the same analytical strategy to investigate the spatial localization of macrophage subpopulations (Fig. S13b). To validate the effectiveness of this approach, we also conducted comparative analyses with the SPOTlight.^57^ The results demonstrated comparable results between the two methods (Fig. S13c). Notably, periosteal cell 1 (*Ctsk*^*+*^*Fmod*^*+*^ PCs) migrated around the bone graft substitute, while they remained in situ in the periosteum in the Ctrl group (Fig. 4b). These findings were further validated by immunofluorescence staining conducted on day 3 and 7 (Fig. 2b, 4c–e). Both osteoblast-related cells (pre-osteoblasts and osteoblasts) and fibroblast-related cells (fibroblasts) were present around the bone graft substitute, suggesting coexisting fibroblastic and osteogenic activity (Fig. S13d). Subsequently, we examined the spatial localization of PIMs and AIMs. The migration patterns of PIMs and AIMs around the bone graft substitute over time were consistent with *Ctsk*^*+*^*Fmod*^*+*^ PCs (Fig. 4b). Since both PIMs and AIMs highly express *Spp1*, we used *Spp1* and *F4/80* for cell labeling to trace their positional distribution in *vivo*. The results showed that a large number of *Spp1*^*+*^ macrophages were distributed around the bone graft substitute, further validating the results of the ST analysis (Fig. 4e).

### Spatial niches of periosteum around the bone graft substitute

**Fig. 5:**
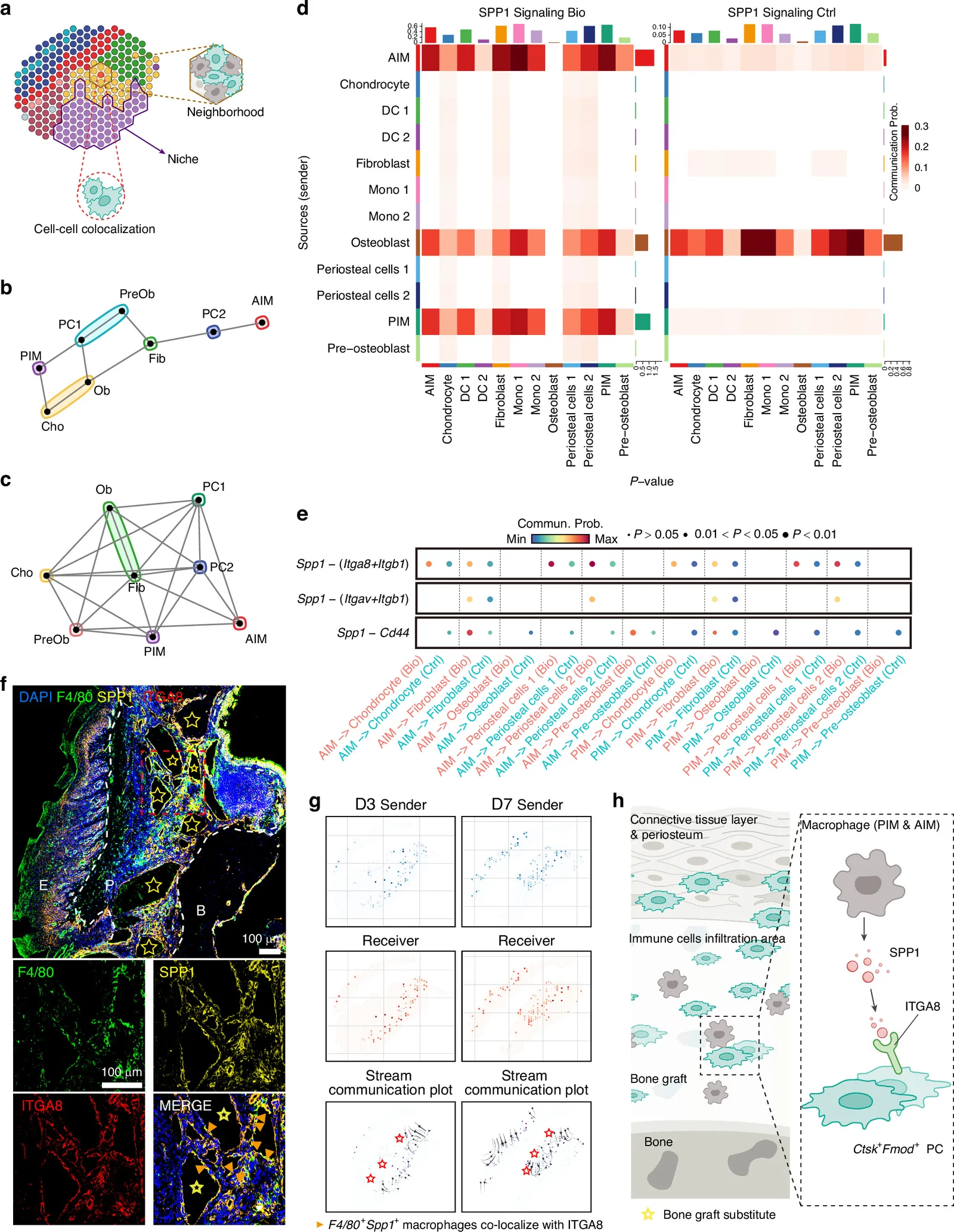
Spatial niches of periosteum around the bone graft substitute. **a** Schematic of cell–cell colocalization, neighborhood and niche definition. Juxta-view cell–cell interaction networks using Misty of day 3 (**b**) and day 7 (**c**). **d** Heatmap of SPP1 signaling pathways in Bio and Ctrl group. **e** Network plot showing the ligand-receptors analysis of SPP1 signaling in Bio and Ctrl group. **f** Representative confocal images of sample section. The *F4/80*^*+*^*Spp1*^*+*^ macrophages co-localize with *Itga8* (yellow arrows). Stars, bone graft substitute. E epithelium, P periosteum, B bone. **g** The sender, receiver and stream communication plot of Spp1-Itga8_Itgb1 COMMOT signals. Stars, bone graft substitute. **h** Illustration showing the crosstalk between periosteal cell 1 and macrophages subclusters in the subperiosteal microenvironment

To further elucidate the spatial dynamics of cellular interactions of periosteum around the bone graft substitute, we defined different neighborhood area sizes using MISTy: the intra-spot view (~ 55 μm) showing the cell–cell colocalization within a spot and the juxta-view (~ 200 μm) reflecting the local neighborhood (Fig. 5a).^58^ We observed the co-occurrence of periosteal subclusters, including osteoblasts and fibroblasts, within individual tissue spots (intra-spot view). Notably, the *Ctsk*^*+*^*Fmod*^*+*^ PCs (PC1) were located distally within these periosteal subclusters, revealing a close relationship between *Ctsk*^*+*^*Fmod*^*+*^ PCs (PC1) and osteoblasts or fibroblasts (Fig. S14a, b). On day 3, the *Ctsk*^*+*^*Fmod*^*+*^ PCs (PC1) colocalized with pre-osteoblast, informing an osteogenic microenvironment (Fig. 5b). When it turned to day 7, the *Ctsk*^*+*^*Fmod*^*+*^ PCs (PC1) and AIM were close in the space, and the osteoblast co-occurrent with fibroblast (Fig. 5c).

As the cells were more likely to interact with nearby cells,^59^ the close spatial proximity of *Ctsk*^*+*^*Fmod*^*+*^ PCs and AIMs on day 7 suggested that they might undergo cellular communication. We first applied CellChat^60^ to investigate cell–cell interactions including all annotated immune populations and PCs. Among all immune populations, macrophage subsets (especially AIM and PIM) showed the strongest communication with PCs (Fig. S15). Based on these results, we prioritized macrophage-periosteal signaling for downstream analysis. Comparative analysis of interaction strength between the Bio and Ctrl groups revealed heightened signaling pathways in the former, including SPP1 and TGF-β signaling (Fig. S14c). AIMs exhibited the highest interaction strength among all cell types, establishing robust regulatory relationships with *Ctsk*^*+*^*Fmod*^*+*^ PCs (PC1) (Fig. S14d, e).

Next, we compared significant pathways between different clusters in the Bio and Ctrl groups, among which the SPP1 signaling pathway was one of the most prominent (Fig. S14f, g). Macrophages are the main cells expressing SPP1 (Fig. S14h). Further exploration of the SPP1 signaling pathway indicated that AIMs sent strong signals, which were received by subsets of periosteal cells (*Ctsk*^*+*^*Fmod*^*+*^ PCs, periosteal cell 2 and fibroblasts) in the Bio group (Fig. 5d, e). Analysis of *Spp1* expression in macrophages across different groups showed predominant expression in the Bio group, particularly within PIMs and AIMs (Fig. S14i). Spatial plots showed preferential aggregation of ligands (Spp1) and receptors (Itga8+Itgb1; Itga5+Itbg1) around the bone graft substitute (Fig. S14j). We observed co-localization of *F4/80*^*+*^*Spp1*^*+*^ macrophages and *Itga8* in animal samples, Suggesting potential interactions between F4/80^*+*^Spp1^*+*^ macrophages and Itga8^*+*^ cells (Fig. 5f).

Finally, we aim to dissect spatial cell–cell communication by performing COMMOT (COMMunication analysis by Optimal Transport).^61^ There was less SPP1 signal in the Ctrl group at day 3. We confirmed that Spp1-Itga8_Itgb1 COMMOT signals showed the same direction (from immune cells infiltration area to the periosteum) in the Bio group while the Ctrl group had random directionality (especially in the lower left corner of the picture) at day 7 (Fig. 5g). Additionally, we found that the Spp1-Itga8_Itgb1 COMMOT signals were more concentrated around the bone graft substitute at day 7 (Fig. 5g).

In summary, macrophage subpopulations and periosteal cell subpopulations establish intricate ecological niches surrounding the bone graft substitute. Notably, AIMs and *Ctsk*^*+*^*Fmod*^*+*^ PCs exhibit close cellular interactions that influence osteogenesis and fibrogenesis within these niches. AIMs may modulate the biological functions of *Ctsk*^*+*^*Fmod*^*+*^ PCs through the SPP1 signaling pathway (Fig. 5h).

### SPP1 secreted by macrophages reduced the osteogenic capacity of *Ctsk*^*+*^*Fmod*^*+*^ PCs

**Fig. 6:**
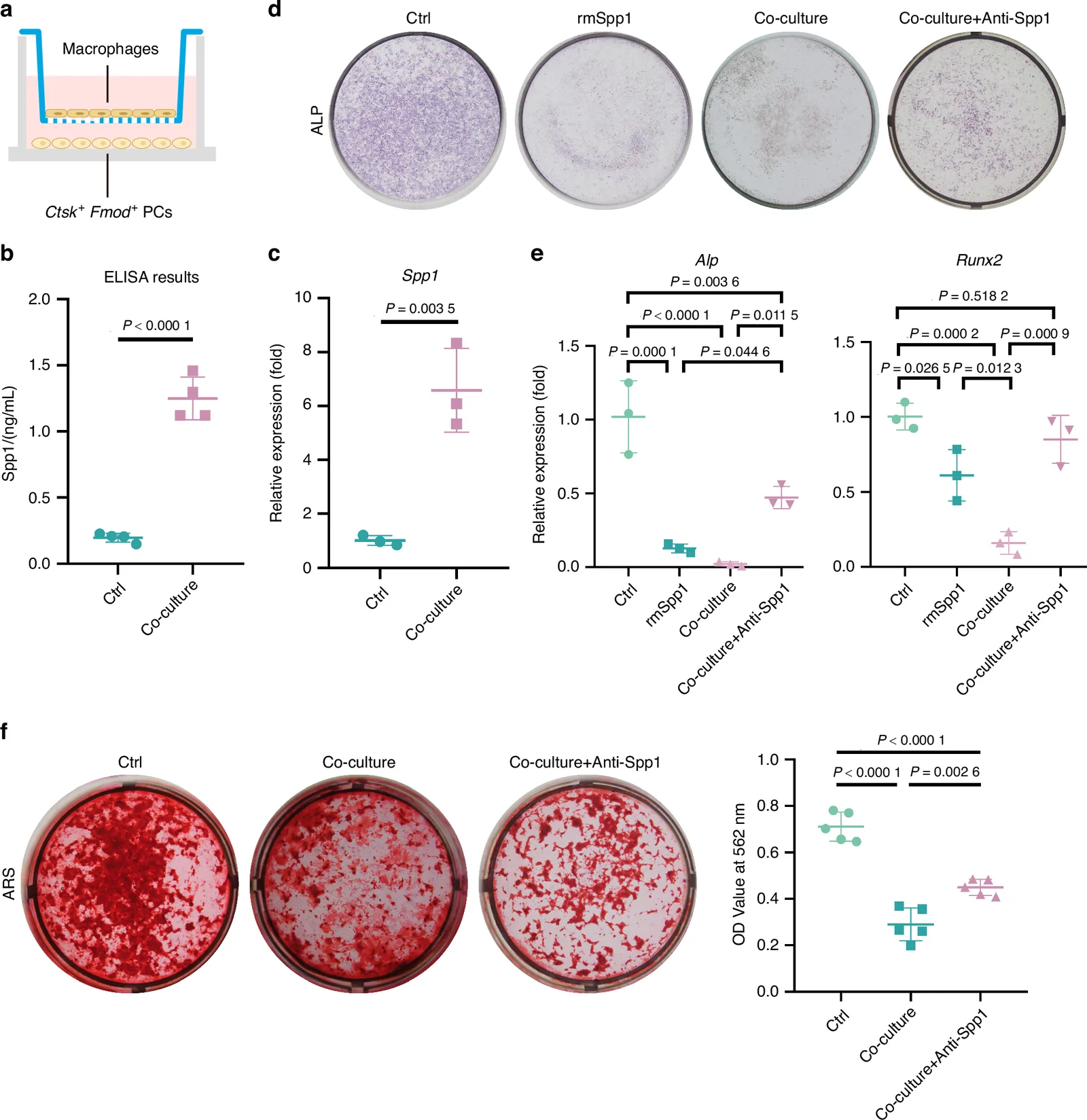
Spp1 secreted by macrophages reduced the osteogenic capacity of *Ctsk*^*+*^*Fmod*^*+*^ PCs. **a** Schematic representation of the co-culture system of macrophages and *Ctsk*^*+*^*Fmod*^*+*^ PCs. **b** ELISA results of upper chamber culture medium in different groups. **c** qPCR analyses of the mRNA levels of *Spp1* in macrophages with or without co-culturing with *Ctsk*^*+*^*Fmod*^*+*^ PCs. **d** ALP staining of PCs in different group on day 7. **e** qPCR analysis of the mRNA levels of *Alp* and *Runx2* of *Ctsk*^*+*^*Fmod*^*+*^ PCs in different group. **f** ARS staining and semiquantitative analysis of PCs in different groups on day 21

To validate the role of SPP1 secreted by macrophages, we co-cultured macrophages and sorted cells via a Transwell system (Fig. 6a). The group without macrophages placed in the upper chamber served as the Ctrl group. Forty-eight hours later, we collected the supernatant from the upper chamber. ELISA results showed elevated secretion of SPP1 by macrophages (Fig. 6b). This was consistent with increased *Spp1* gene expression observed in macrophages within the co-culture system, as demonstrated by PCR analysis (Fig. 6c). In addition, a group supplemented with 2.0 μg/mL mouse recombinant Spp1 protein (rmSpp1) was established.^62^ ALP staining on day 7 revealed that the addition of rmSpp1 to *Ctsk*^*+*^*Fmod*^*+*^ PCs cultures or co-culture with macrophages resulted in decreased osteogenic ability (Fig. 6d). The downregulation of mRNA expression levels of *Alp* and *Runx2* on day 7 in the coculture group could be reversed by blocking SPP1 (Fig. 6e). ARS staining and semiquantitative analysis on day 28 further demonstrated that blocking SPP1 improved the osteogenic effect of *Ctsk*^*+*^*Fmod*^*+*^ PCs (Fig. 6f). We also isolated Bone Marrow-Derived Macrophage (BMDM) from wild-type (WT) and SPP1 knockout (KO) mice. Then these cells (Mφ and KO-Mφ) were co-cultured with *Ctsk*^*+*^*Fmod*^*+*^ PCs respectively (Fig. S16a, b). Consistent with the previous results, *Ctsk*^*+*^*Fmod*^*+*^ PCs co-cultured with KO-Mφ showed stronger osteogenic ability (Fig. S16c–e). This further supports that macrophage-derived SPP1 negatively regulates the osteogenic potential of *Ctsk*^*+*^*Fmod*^*+*^ PCs.

**Fig. 7:**
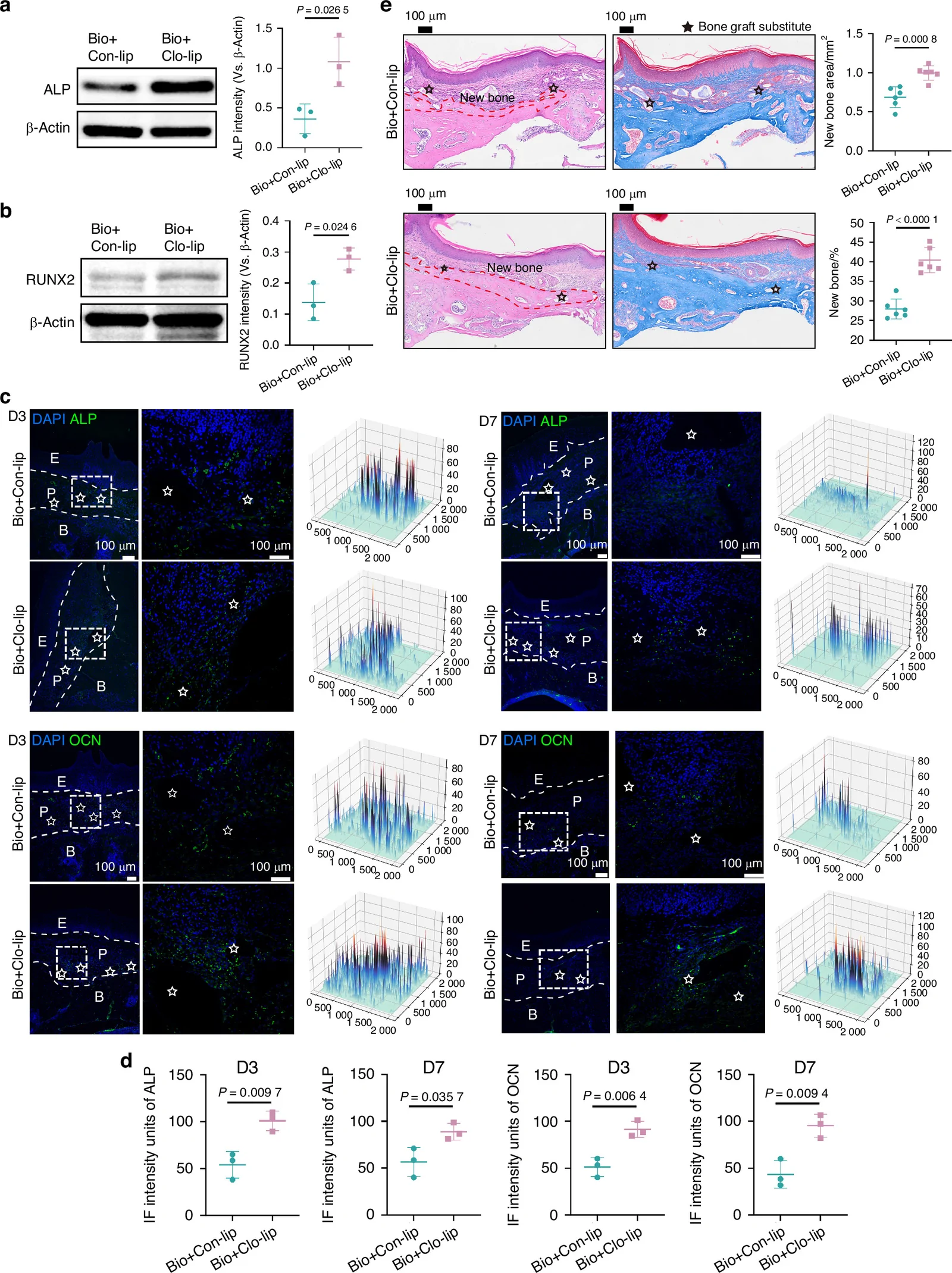
The role of Spp1 secreted by macrophages on *Ctsk*^*+*^*Fmod*^*+*^ PCs. **a** Workflow of macrophages depletion in vivo. Western blot analysis of ALP (**b**) and RUNX2 (**c**) expressed in two groups. β-Actin was used as a reference protein and corresponding quantification of the ALP and RUNX2 level. **c** Histological staining and 3D surface plot of fluorescence intensity of ALP and OCN in operation areas at day 3 and day 7. Stars, bone graft substitute. E epithelium, P periosteum, B bone. **d** Normalized fluorescence intensity at day 3 and day 7 in different groups. **e** Histological staining of H&E and Masson after 28 days and semiquantitative analysis of new bone area and the percentage of new bone. Stars, bone graft substitute. Red dotted line, new bone area

To further explore the effect of macrophages on *Ctsk*^*+*^*Fmod*^*+*^ PCs in vivo, we locally depleted macrophages at the surgical site via clodronate liposomes injection (named Bio+Clo-lip group)^63^ (Fig. S17a). The control group used the control-liposomes (named Bio+Con-lip group). Immunofluorescence and flow cytometric analysis confirmed a reduction of macrophages at the surgical site after drug injection (Fig. S17b). Furthermore, the depletion of macrophages significantly reduced local *Spp1* gene expression at the surgical site (Fig. S17c, d). Western blot analysis revealed higher expression levels of osteogenesis markers, ALP and RUNX2, in the Bio+Clo-lip group compared to the Bio+Con-lip group on day 7 (Fig. 7a, b). Similarly, immunofluorescence results also showed higher *Alp* and *Ocn* expression in the Exp group (Fig. 7c, d). Histological examinations further demonstrated that new bone formation increased following the depletion of macrophages and downregulation of *Spp1*, as evidenced by semiquantitative analysis of new bone area (Fig. 7e). Meanwhile, we observed that there was no significant difference in the total number of *Trap*^*+*^ cells between two groups (Fig. S18). This suggests that the number of osteoclasts were not significantly affected by the clodronate liposomes. However, we cannot fully exclude the possibility that altered osteoclast activity contributed to the increased bone mass observed after clodronate liposome treatment. Available evidence suggests that substantial macrophage reduction enhances osteogenic activity at the surgical site.

**Fig. 8:**
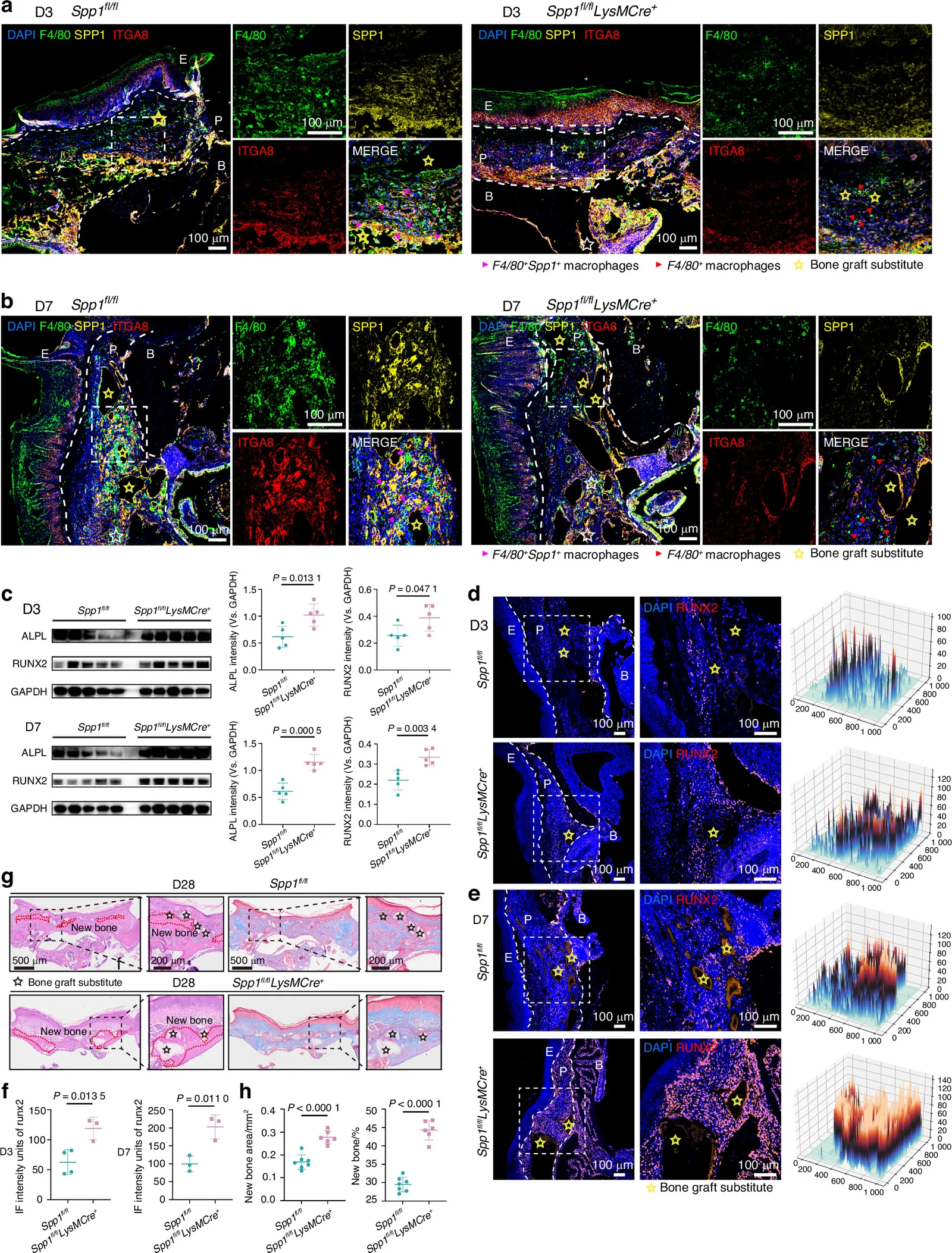
Deletion of *Spp1* in macrophages improved the osteogenic capacity of *Ctsk*^*+*^*Fmod*^*+*^ PCs. Representative confocal images of sample section of day 3 (**a**) and day 7 (**b**) in different groups. The *F4/80*^*+*^*Spp1*^*+*^ macrophages (pink arrows) co-localize with *Itga8* in *Spp1*^*fl/fl*^ mice. However, only F4/80^*+*^ macrophages (red arrows) could be found in *Spp1*^*fl/fl*^*LysMcre*^*+*^ mice. Stars, bone graft substitute. **c** Western blot analysis of ALP and RUNX2 expressed in different groups. GAPDH was used as a reference protein and corresponding quantification of the ALP and RUNX2 level. Histological staining and 3D surface plot of fluorescence intensity of RUNX2 in operation areas at day 3 (**d**) and day 7 (**e**) in different groups. Stars, bone graft substitute. E epithelium, P periosteum, B bone. **f** Normalized fluorescence intensity at day 3 and day 7 in different groups. **g** Histological staining of H&E and Masson after 28 days in different groups. Stars, bone graft substitute. Red dotted line, new bone area. **h** Semiquantitative analysis of new bone area and the percentage of new bone

Then we used *Spp1* flox/flox mice crossed with *LysM*-cre transgenic mice to obtain conditional homozygous *Spp1* knockout mice (*Spp1*^fl/fl^*LysMcre*^*+*^). The conditional knockout of Spp1 had no influence on the size and weight of the mice with their *Spp1*^*fl/fl*^ control littermates. Then we established the subperiosteal bone grafting model on these mice and harvested samples at days 3, 7, and 28 post-operation (Fig. S19a). We compared the expression of the SPP1 signaling pathway between different groups. The results demonstrated that *Spp1*^*fl/fl*^ control littermates exhibited a higher abundance of *F4/80*^*+*^*Spp1*^*+*^ macrophages co-localized with *Itga8* compared to *Spp1*^fl/fl^*LysMcre*^*+*^ mice (Fig. 8a, b). Recent work revealed that the SPP1 signaling pathway is triggered by the interaction between SPP1 and integrin, which subsequently leads to the activation of AKT pathways.^64^ Western blot indicated that the PI3K/AKT/mTOR was activated in *Spp1*^*fl/fl*^ control mice (Fig. S17e). The *Spp1*^fl/fl^*LysMcre*^*+*^ mice showed higher osteogenesis markers (ALP and RUNX2) according to the Western blot analysis (Fig. 8c). Additionally, knockout of *Spp1* of myeloid cells revealed an up-regulation of osteogenic markers (*Runx2*) on day 3 and 7, as evidenced by the immunofluorescence results (Fig. 8d–f). On day 28, we analyzed the new bone area across different groups. The results revealed that new bone formation was enhanced when *Spp1* expression in macrophages was reduced (Fig. 8g, h).

Finally, we used the integrin receptor antagonist GLPG - 0187 to preliminarily explore the role of the “SPP1 - Integrin” axis on the osteogenic ability of *Ctsk*^*+*^*Fmod*^*+*^ PCs.^65^ In vitro, *Ctsk*^*+*^*Fmod*^*+*^ PCs were treated with PBS, mouse recombinant SPP1 protein (rmSPP1), or a combination of rmSPP1 and GLPG-0187. The results indicated that the addition of GLPG-0187 partially rescued ALP activity and mineralized nodule formation (Fig. S19a, b). These in vitro findings were corroborated by in vivo experiments, where GLPG-0187 treatment led to an increase in new bone formation, as confirmed by Micro-CT analysis and histological semiquantification (Fig. S19c–f).

The above results indicated that macrophages recruited to the surgical site secreted more SPP1, impairing the osteogenic ability of *Ctsk*^*+*^*Fmod*^*+*^ PCs. Blocking SPP1 enhanced the osteogenic potential of *Ctsk*^*+*^*Fmod*^*+*^ PCs and promoted osteogenesis at the surgical site.

## Discussion

The periosteum has the ability to regenerate bone and can be used for bone defect repair.^66^ With the help of scRNA-seq, increasing evidence shows that functionally relevant cellular heterogeneity exists in the periosteum.^9,10,13,15^ However, normal cellular functions depend on the interaction of neighboring individual cell types. Understanding the spatial interactions and functional dynamics of periosteal cells is essential for uncovering the mechanisms underlying bone regeneration. Analyzing the traits and spatial characteristics of jaw-bone periosteal cells provides critical insights into this process. In this study, we map the jaw-bone periosteal microenvironment surrounding the bone graft substitute, offering a novel perspective on the spatial dynamics of the jaw-bone periosteum involved in osteogenesis.

First, we identify the key markers of PC subsets and dissect functional PC subpopulations. PCs of jaw bone included different cell subtypes with different functions, among which the *Ctsk*^*+*^*Fmod*^*+*^ subset might be a population of cells with differentiation potential. *Ctsk* labels a population of progenitors in the long bones and calvarial sutures.^9^ *Fmod*, encoding fibromodulin, is a member of small interstitial leucine-rich repeat proteoglycans (SLRPs).^67^ Studies have showed that *Fmod* is more commonly distributed in oral soft tissues and contributes to maintaining the bone phenotype.^68,69^ In this study, we found that *Ctsk* and *Fmod* labeled a subset of PCs. Recent study found that *Dlx5*^*+*^ fetal perichondrial cell could become an adipogenic subset of stromal cells.^70^ In our study, the *Ctsk*^*+*^*Fmod*^*+*^ subset did not express *Dlx5*, suggesting that different periosteal cell subpopulations may have distinct differentiation fates.

Through ST and immunofluorescence, we found that this population was in the periosteal layer, and when the periosteum was activated by bone graft substitute, these cells gradually migrated around the material. Notably, it was difficult to find the classic periosteal bilayer structure on day 7 in the Bio group, which may be related to the migration and remodeling of periosteal cells (Fig. 2d, m). Furthermore, in vitro colony formation tests and differentiation induction assays validated *Ctsk*^*+*^*Fmod*^*+*^ PCs as cells with good proliferation ability and differentiation potential. *Ctsk*^*+*^*Fmod*^*+*^ PCs migrating around the material differentiated into osteoblasts and participated in local osteogenic activities.

The activation of *Ctsk*^*+*^*Fmod*^*+*^ PCs is likely the result of a combination of factors, including the surgical elevation of the periosteum, local mechanical and matrix cues provided by the bone graft substitute, and graft-associated inflammatory signaling. By combining scRNA-seq and ST, we were able to dissect the characteristic of periosteal microenvironment around the bone graft substitute. Interactions between immediate neighboring spots revealed dependencies between *Ctsk*^*+*^*Fmod*^*+*^ PCs and AIMs. We also defined the niches surrounding the bone graft substitute. Unlike early stage (day 3), the periosteal stromal cells (especially the *Ctsk*^*+*^*Fmod*^*+*^ PCs) and immune cells (AIMs and PIMs) formed a complicated spatial niche (niche 5) around the bone graft substitute and weakened local osteogenic activity. Our analysis of macrophage composition revealed that AIMs expressed higher levels of *Spp1* and engaged in close interactions with *Ctsk*^*+*^*Fmod*^*+*^ PCs through Spp1 (in AIMs) and Itga5/Itga8+Itgb1(in *Ctsk*^*+*^*Fmod*^*+*^ PCs). The higher expression of *Spp1* could be attributed partly to the foreign body reaction (FBR). Previous studies have highlighted that in the FBR reaction, interactions of giant cells (GCs) are active and Spp1 (GC)-Cd44 (fibroblast) interactions are prominent.^71^ Notably, we observed an increase in *Spp1* expression in macrophages within the coculture system, suggesting that the effect of *Ctsk*^*+*^*Fmod*^*+*^ PCs on macrophages also contributed to the upregulation of *Spp1* in the Bio group.

*Spp1*, which is also known as osteopontin, exerts significant influence on various cellular functions, including cell adhesion, migration and survival via CD44.^72^ It serves as a key regulator of hematopoietic stem cells (HSCs) and acts as a physiologic-negative regulator of HSCs proliferation.^73,74^ SPP1 neutralization has been shown to impact the response of liver progenitor cells and reduce fibrogenesis.^75^ *Spp1* encodes the osteopontin protein and serves as an inflammatory and profibrotic marker.^26^ Macrophage-derived Spp1 could regulate stromal progenitor differentiation.^23^ However, the role of Spp1 in periosteal cells has not been elucidated. Recent work demonstrated a subset of *CD9*^*+*^*TREM2*^*+*^ macrophages, marked by *Spp1*, *Gpnmb*, *Fabp5* and *Cd63*, which played a profibrotic role in hepatic and pulmonary fibrosis. Within the subperiosteal spatial niche, we observed that AIMs also highly expressed these markers and engaged in close cellular interactions with *Ctsk*^*+*^*Fmod*^*+*^ PCs. Furthermore, SPP1 secreted by macrophages impaired the osteogenic ability of *Ctsk*^*+*^*Fmod*^*+*^ PCs, leading to a decrease in the expression of osteogenesis-related genes, and weakening of osteogenesis. In addition, blocking SPP1 restored the osteogenic potential of *Ctsk*^*+*^*Fmod*^*+*^ PCs and increased the amount of new bone formation on day 28.

Moreover, we found that the PI3K/AKT/mTOR was activated in *Spp1*^*fl/fl*^ control mice. Research has demonstrated that the PI3K/AKT/mTOR pathway plays a crucial role in tissue fibrosis.^76,77^ Blocking the PI3K/AKT/mTOR pathway alleviate pulmonary fibrosis by enhancing autophagy and suppressing activation of lung fibroblasts.^78^ However, conflicting results regarding the influence of the PI3K/Akt/mTOR pathway on the osteogenic differentiation capacity of stem cells persist. Tanaka’s research revealed that the suppression of the PI3K/AKT/mTOR signaling pathway significantly enhances the osteogenic and dentinogenic differentiation potential of stem cells derived from the apical papilla.^79^ Another study demonstrated that the activation of autophagy, mediated by the PI3K/Akt/mTOR signaling cascade, alleviates impaired osteogenesis in adipose-derived stem cells within a diabetic microenvironment.^80^ However, Pantovic et al. discovered that the late-stage activation of the Akt/mTOR signaling axis is essential for the differentiation of stem cells.^81^ We found that macrophage derived SPP1 suppress the osteogenic ability of *Ctsk*^*+*^*Fmod*^*+*^ PCs via the activation of PI3K/AKT/mTOR pathway. In our modified GBR model, *Ctsk*^*+*^*Fmod*^*+*^ PCs are subjected to a graft-associated inflammatory and foreign-body-like microenvironment, characterized by a high enrichment of macrophage-derived SPP1. Under these conditions, the activation of the PI3K/AKT/mTOR pathway by macrophage-derived SPP1 is more likely to promote fibrotic signaling rather than pro-osteogenic processes. More research employing pathway-specific activation and inhibition is needed to further explore the impact of the PI3K/AKT/mTOR pathway on *Ctsk*^*+*^*Fmod*^*+*^ PCs.

Although this study expanded our understanding of spatial characteristics of jaw-bone periosteum, it has certain limitations. As the limited resolution rate of ST data, it’s difficult to capture the finer cellular dynamics. Although we depleted the *Spp1* expression of macrophage, it would be better to specific deletion *Spp1* of AIMs to assess their role on *Ctsk*^*+*^*Fmod*^*+*^ PCs. More samples were needed to further dissect the spatial architecture of jaw-bone periosteum under different conditions.

In conclusion, this study revealed the heterogeneity of PCs in the jaw bones, highlighting the multidirectional differentiation potential of the *Ctsk*^*+*^*Fmod*^*+*^ subset. Moreover, the integrated spatial atlas allowed us to better understand the spatial dynamics of the jaw-bone periosteum involved in osteogenesis. The complicated spatial niche including AIMs and *Ctsk*^*+*^*Fmod*^*+*^ PCs exhibits a variety of biological processes, such as osteogenesis and fibrogenesis. Interaction between AIMs and *Ctsk*^*+*^*Fmod*^*+*^ PCs, mediated by SPP1 signaling, negatively impacts *Ctsk*^*+*^*Fmod*^*+*^ PCs osteogenic capacity, which can be partially rescued by blocking SPP1 (Fig. S20). These findings highlight our current understanding of jaw-bone periosteal cells and suggest implications for therapeutic interventions targeting bone defects.

## Materials and methods

### Animals

Wild-type, male SD rat purchased from Chengdu Dossy Experimental Animals Co., LTD. were six-week-old and had an average weight of 200 g. Spp1-flox mice (Strain No. T012963), Spp1-KO mice Strain (Strain No. T64052), H11-Ctsk-iCre mice (Strain No. T006950) and B6/JGpt-Rosa26^tm1(CAG-LSL-tdTomato)^/Gpt (Strain No. T002249) was purchased from GemPharmatech (Nanjing, China). And Lyz2-icre mice were offered by Dan Liu’s Lab from Westlake University. All the animal care and treatment procedures were carried out in accordance with international standards on animal welfare and approved by the Research Ethics Committee, West China Hospital of Stomatology, Sichuan University (WCHSIRB-D-2019-087).

### Modified GBR model

Briefly, after anesthesia, the palatal gingiva adjacent to the tooth was gently separated using a dental probe to establish the initial surgical access. A Buser periosteal elevator was then inserted through this access, and the mucoperiosteal tissue was carefully elevated from the palatal cortical bone surface to create a subperiosteal pocket while preserving the integrity of the periosteum. Next, Bio-Oss Collagen was placed between the periosteum and hard palate as the experimental group (named Bio group) and a blank control group (named Ctrl group) was set up. The surgical procedures of the Ctrl group were identical to those in the Bio group, including palatal gingival separation, elevation of the periosteal tissue, repositioning of the elevated periosteum, and suturing. Before implantation, the Geistlich Bio-Oss Collagen®(100 mg) was precisely cut using sharp, sterile scissors into pre-defined cuboid pieces. Specifically, each rat received a bone graft substitute with a volume of 3 mm × 2 mm × 2 mm, whereas each mouse received a bone bone graft substitute with a volume of 2 mm × 2 mm × 1.5 mm. The dimensions of each implant were verified before implantation using a ruler (Fig. S2). Finally, we sutured the surgical area with cross-stitch using 7-0 suture.

### Patient recruitment, ethical approval and biopsy specimen harvest

All healthy volunteers and patients participating in this study provided written informed consent prior to the treatment. This study was approved by the Research Ethics Committee, West China Hospital of Stomatology, Sichuan University (WCHSIRB-D-2019-099).

Patient inclusion and exclusion criteria were consistent with our published studies.^32^ Inclusion criteria for this study were: over 18 years of age, had received a single tooth replacement with a dental implant, had received horizontal bone augmentation with modified GBR, and had more than three months of healing.^32^ Exclusion criteria for this study were: patients with serious systemic diseases, patients who were pregnant or heavy smokers, and patients with uncontrolled periodontal disease.

For histological analysis, we took biopsy specimen from patients receiving a second-stage surgery. The excess bone overing the cover screw was taken from the bone augmentation area. For scRNA-seq, gingival biopsy (4 mm × 2 mm) from a healthy volunteer was obtained under local anesthesia.

### Sample processing, histology and histomorphometry

After healing for 3, 7, 14 and 28 days, animals were euthanized for sample harvest. The harvested maxillae were fixed in 4% paraformaldehyde overnight at 4 °C and then stored in 75% ethanol for subsequent experiments. After fixed, the samples were decalcified in 10% EDTA for 4 weeks. Before embedded in paraffin, the samples were dehydrated through an ascending ethanol series. Longitudinal sections were obtained for H&E staining and Masson staining and the histology of the section was observed.

Histomorphometric analysis of new bone formation was performed on H&E- and Masson-stained sections. Firstly, the Masson staining was used to roughly delineate the range of new bone. Newly formed bone was identified primarily as light-blue stained mineralized matrix within the grafted region. New bone was further defined by histological morphology, including continuity with bone-forming fronts and the enclosure or bridging of residual bone graft substitue by newly deposited bone matrix. For quantification, the bones between the teeth were first defined as the ROI at low magnification. Within this ROI, newly formed bone was annotated based on the above criteria and calculated. We simultaneously counted the area of the new bone region (new bone area) and the proportion of the new bone in the ROI (new bone%).

### Immunofluorescence and image analysis

The experimental procedure of immunofluorescence was consistent with our previous study.^46^ To determine osteogenesis in the subperiosteal region, we chose Alpl (Abcam, ab203106, 1:100), Runx2(Abcam, ab192256,1:500) and Osteocalcin (Abcam, ab93876, 1:200). To locate macrophage and expression of Spp1, we chose CD68 (Abcam, ab222914, 1:100), F4/80 (Abcam, ab111101,1:100), Itga8 (Abcam, ab243027, 1:200) and Osteopontin (Abcam, ab8448, 1:1 000). Immunofluorescence staining for Cathepsin K (Abcam, ab37259, 1:200) and Fibromodulin (Invitrogen, PA5-26250, 1:100) were performed for determination of the location of the PSCs. Briefly, we used the multiplex fluorescence immunohistochemistry kit (Panovue, Cat. 10203100020) for staining. After incubating the primary antibody at room temperature for 1 h, we used the goat anti-rabbit secondary antibody in the kit (incubated at room temperature for half an hour) and the monochromatic fluorescent dye (incubated at room temperature for 15 min) for subsequent staining. Finally, we used a confocal microscopy (Olympus) to capture images and ensured that the acquisition parameters were consistent.

To determine the composition of the cells that we sorted through Fluorescence activated Cell Sorting (FACS), sorted cells were washed with PBS(Gibco) and fixed in 4% paraformaldehyde for 30 min. Then cells were washed in 0.5% Triton X-100(Biofroxx)/PBS, blocked in 5% BSA(Biofroxx) and incubated with primary antibodies (Cathepsin K and Fibromodulin) at 4 °C overnight. The next day they were incubated with FICT (APExBIO, K1201, 1:400) and APC (Abcam, ab130805, 1:400) for 1 h at room temperature. DAPI solution (Solarbio) was added and incubated for 30 min at room temperature without light. Confocal microscopy (Olympus) was used to image the stained sections.

### Single cell RNA sequencing

Specimen harvest procedure for scRNA-seq was reported in our previous reports.^46^ Briefly, we obtained three fresh samples from the surgical area per group (Bio and Ctrl) at different timepoints (day 3 and day 7), containing the hard palatal mucosa, periosteum, and the maxillae hard palate. We cut the specimen into 1 mm width pieces and mixed them with type I collagenase (Gibco) and trypsin (Hyclone) for sample digestion at 37 °C lasted for 2.5 h. After dissociating, filtering, centrifuging the suspensions, we resuspended them in 3 mL of red blood cell lysis buffer (Biosharp). Then we remove the dead cells and debris by Dead Cell Removal Kit (Miltenyi). Last, 10× Genomics Chromium Single Cell 3′ v3 sequencing was performed using an Illumina 1.9 mode.

### Single-cell RNA-sequencing analysis

We used Cellranger pipeline software (V3.0) to generate the expression matrices for downstream analysis. After completing quality control and count normalization, we used the canonical correlation analysis for batch correction and integrated analysis (Seurat V4.1.2). Then we performed subpopulation analysis of PCs and macrophages. To infer the developmental progression of different subpopulation of PCs, we used the Monocle 2 package (V2.10.0) to order them in pseudotime. And we used Cellchat (V1.5.0) to predict communications among cell subpopulations.

### Fluorescence activated cell sorting (FACS) and cell cultures

To isolate *Ctsk*^*+*^*Fmod*^*+*^ and *Ctsk*^*+*^*Fmod*^−^ PCs, we obtained the periosteum from Jaw bones of 4-week-old SD rats (Chengdu Dossy Experimental Animals Co., LTD) and human sample. The harvested periosteum was minced and digested with Dispase II (2 mg/mL; Roche) and Collagenase P (1 mg/mL; Roche) for 45 min at 37 °C. Isolated periosteal cells were cultured and expanded for 1 week before FACS. Subsequently, we digested the cells to obtain a single-cell suspension. The suspensions were incubated in the dark with FVD780 (eBioscience, 1:1 000) for 30 min and primary antibody Cathepsin K (Abcam, ab37259, 1:300) and Fibromodulin (Invitrogen, PA5-26250, 1:300) for 1 h. After being washed 2 to 3 times with 0.5% PB buffer, the cell solutions were co-incubated with CD45 (Biolegend, 202205, 1:400), Ter-119 (BIOSS,BS-0468R-FITC, 1:100), CD31 (Biolegend, 116206, 1:50), PE Goat Anti-Mouse IgG (H + L) Antibody (Biolegend,405307,1:50) and APC Anti-Rabbit IgG (H + L) Antibody (Abcam, ab130805,1:400) for 0.5 h. FACS was performed using BD FACS Ariall and analysis was performed using FlowJo 10.8.1.

Sorted cells were cultured in DMEM media (Gibco) with 20% FBS (ZETA LIFE) in 5% CO_2_ at 37 °C. We replaced half of the media every 3 days and passaged cells once they were 70%–80% confluent.

### Colony formation test

500 sorted cells per well were seeded in 6-well plate. The medium was added as described above and it was changed every 3 days. After 10 days of culture, cells were fixed and stained with 1% crystal violet solution. Images were then taken with stereomicroscope (Olympus).

### Osteogenic, adipogenic and chondrogenic differentiation

For osteogenic induction, sorted cells were expanded and then cultured in osteogenic differentiation medium with ascorbic acid (50 μg/mL), β-glycerophosphate (5 mmol/L), and dexamethasone (100 nmol/L). Media was changed every 2 days. 7 days later, ALP staining was performed with Alkaline Phosphatase Assay Kit (Beyotime). And after 14 days of induction, Alizarin red staining (ARS) was performed. 1% ARS Solution (Solarbio) was used to stain the mineralized nodules. Cells were then washed thoroughly for analysis. 1% hexadecylpyridinium chloride (Solarbio) was added to degrade the mineral nodules. Finally, the solution was collected for detecting at 562 nm.

For adipogenic induction, sorted cells were allowed to differentiation in adipogenic differentiation medium (Cyagen Biosciences, RAXMX-90031) and changed every 3–4 days for a total of 21 days. Being washed with PBS and fixed with 4% Paraformaldehyde, cells were stained with Oil Red O Staining Kit (Beyotime) and then observed under a light microscope (Olympus).

For Chondrogneic differentiation, chondrogenic differentiation medium (Cyagen Biosciences, RAXMX-90041) was used in cell cultures. The media was changed every 3 days and allowed to differentiation for 28 days. Then, after being washed with PBS and fixed, cells were stained with 1% Alcian blue solution (Sigma).

### Spatial transcriptions and spatial transcriptomic analysis

Fresh full-thickness tissue above the bone surface was harvested at different timepoints (day 3 and day 7) and groups (Bio and Ctrl). No decalcification was performed before cryosectioning. Immediately after collection, samples were gently rinsed with ice-cold RNase-free PBS, embedded in OCT compound, snap-frozen in liquid nitrogen, and stored at −80 °C until further processing. The samples were cryosectioned to get gene expression slides. After fixation, staining and imaging, the slides from both groups were ready for tissue permeabilization with the Visium Tissue Optimization Slide & Reagent kit. After reverse transcription, the spatially barcoded cDNA was released and collected for ST library preparation. Only samples meeting the required quality criteria were used for library preparation. The P5 and P7 primers, used in Illumina amplification, would be included in the final libraries.

We used SpaceRanger software to process raw FASTQ files and aligned histology images. The gene-spot matrices were analyzed with the Seurat package (V4.1.2). We used the SCTransform function to perform normalization across spots and independent component analysis (ICA) for dimensionality reduction. RCTD was used to accurately assign cell types to ST spots.^51,52^ MIA was used to further confirm the result of RCTD method.^53–55^ The top 300 up-regulated marker genes with adjusted *P* value < 0.01 and average log_2_FC > 0.5 were used to calculate MIA enrichment scores.

To generate the spatial cluster gene signature overlap correlation matrix, we used the FindAllMarkers function to find top 50 markers of each cluster on the single-cell level. Then we used the AddModuleScore function in Seurat to derive signature scoring from scRNA-seq and ST signatures.^48,56^ And we generated spatial feature expression plots with the SpatialFeaturePlot function. To validate the effectiveness of this approach, we also conducted comparative analyses with the SPOTlight (V1.7.2).^57^

### Spatial map of cell dependencies and spatial niches in jaw-bone periosteum

The MISTy was used to define different neighborhood area sizes: the intra-spot view (~55 μm) and the juxta-view (~200 μm).^58^ To define the niche definitions from ST data, we followed the method from Ricardo et al. with the Scran package (v1.18.5). The COMMOT^61^ was used to infer cell–cell communication in ST data.

### Isolation of macrophages

The macrophages were isolated from femur and tibia from 6-week male rats (Chengdu Dossy Experimental Animals Co., LTD). Briefly, the long bones were dissected and the bone marrow was flushed out. After centrifugation, the cells were resuspended in 5 mL of red blood cell lysis buffer (Solarbio). Resuspended in α-MEM medium (Gibco) for more than 16 h, the suspended cells were collected, centrifuged, and resuspended in α-MEM medium with 10% FBS, 1% penicillin/streptomycin(Thermo) and 50 ng/mL M-CSF (Peprotech).

### Co-culturing of PSCs and macrophages

For transwell co-culturing, 2 × 10^4^ sorted cells were seeded into 24-well plate. The 0.4 μm-pore size Corning transwell inserts (Sigma) containing 1 000 macrophages were placed into the 24-well plate with sorted cells. The sorted cells were cultured in osteogenic differentiation medium and changed every 3 days. ALP staining and ARS staining were performed as described above at 7 and 21 days later.

### Elisa

Spp1 levels were measured by collecting the supernatant from the upper chamber of the transwell co-culture system after 24 h. The concentrations of Spp1 were determined according to the kit’s protocol (Jianglai Biotechnology).

### qPCR

Total RNA of PSCs and macrophages of the transwell co-culture system was purified with FastPure Cell/Tissue Total RNA Isolation Kit V2 (Vazyme). cDNA was synthesized using HiScript III RT SuperMix for qPCR (Vazyme). The Taq Universal SYBR qPCR Master Mix (Vazyme) was used for qPCR on PCR instrument (qTOWER3G, Analytik Jena). Gapdh was used as an endogenous control and the $2^{-{\Delta}{\Delta}{{\rm{Ct}}}}$ method was used to calculate the relative expression level of mRNA.

### Macrophage depletion in rat subperiosteal bone grafting model

Macrophages were depleted by local injection of 100 μL clodronate Liposomes (YEASEN) every 3 days. The ctrl group was injected with 100 μL control Liposomes (YEASEN). After healing for 3, 7, and 28 days, animals were euthanized for sample harvest. H&E staining, Masson staining, and immunofluorescence were performed when samples were fixed, decalcified and embedded in paraffin. The ImageJ software was used to analyze the results.

### Flow cytometry analysis

To confirm the success of macrophage depletion model, the fresh samples from the surgical area per group were obtained on day 3 and 7. After obtaining cell suspension by the above method, the cells were suspended in 300 μL staining buffer and incubated on ice for 30 min with FVD780 (eBioscience, 1:1 000) and then with CD45 (Biolegend, 202205, 1:300) for 30 min. For intracellular antibody staining, cells were permeabilized and stained with CD68 (Abcam, ab63856, 1:5 000) for 30 min. The Beckman CytoFLEX Analyzer was used to test samples and Flowjo software (V10.8.1) was used to analyzed and visualized the flow cytometry data.

### Western blot assay

Protein expression was analyzed by western blot assay. Briefly, samples were harvested after 7 days. The total protein of samples was calculated with the BCA protein kit (Servicebio). 20 μg proteins were separated on 4%–12% NuPAGE gel (Solarbio) and transferred to polyvinylidene fluoride membranes (Immobilon-P). After blocking with 5% BSA for 1 h, the membranes were incubated with primary antibody followed by incubating with secondary antibodies. Chemiluminescence reagents (Servicebio) were added to the membranes and the targeted protein bands were visualized. The antibody information was as follows: ALP (AFFINITY, DF12525, 1:2 000), RUNX2 (BIOSS, BS-1134R, 1:2 000), p-AKT (Cellsignal, #4060, 1:2 000), AKT (Cellsignal, #9272, 1:1 000), p-PI3K (Cellsignal, #4228, 1:1 000), PI3K (Cellsignal, #4257, 1:1 000), p-mTOR (Cellsignal, #4060, 1:1 000), mTOR (Cellsignal, #2983, 1:1 000) and β-actin (Servicebio, GB15003, 1:2 000). The Image J software was used to quantify the total gray of the targeted bands.

### Inhibition of integrin

GLPG-0187 (MedChemExpress, HY-100506) was used to inhibit RGD-binding integrins. For in vitro experiments, *Ctsk*^*+*^*Fmod*^*+*^ PCs were treated with PBS, rmSPP1, and rmSPP1 in the presence of GLPG-0187 at 20 nmol/L for 2 h. For in vivo experiments, GLPG-0187 was administered at 50 mg/kg intraperitoneally administered daily.

### Micro-computed tomography (CT) assay

Mouse maxilla samples were scanned using micro-CT (VivaCT 80, SCANCO Medical AG) at a voltage of 55 kV and current of 145 μA with a 200 ms exposure time (integration time), and a voxel size of 10.4 μm. Following 3D volume rendering of the scan data using analytics software (SCANCO), we define the first appearance of the bone graft substitute and the subsequent 140 layers as the the region of interest (ROI) area. bone volume/total volume (BV/TV) and bone mineral density (BMD) were then calculated to compare new bone formation among the different groups.

### Statistics

We dealt the data with Case Viewer software, ImageJ software, and GraphPad Prism 8.0 software. The statistical significance was analyzed using analysis of one-way analysis of variance (ANOVA) with Tukey post-hoc test or Student’s *t* test. *P* < 0.05 was considered statistically significant and *P* > 0.05 was marked as ns (not significant). Exact repeat numbers of each experiment were indicated in the figure legends.

## Supplementary information

Supplementary materials

## Data Availability

All data supporting the findings of this study are available within the article and the Extended Data files. The raw sequence data reported in this paper have been deposited in the Genome Sequence Archive (Genomics, Proteomics & Bioinformatics 2025) in National Genomics Data Center (Nucleic Acids Res 2025), China National Center for Bioinformation / Beijing Institute of Genomics, Chinese Academy of Sciences (GSA: CRA046828; CRA046848) that are publicly accessible at https://ngdc.cncb.ac.cn/gsa. No original code is reported and the code is available from the corresponding author upon request.

## References

[1] Seeman, E. Periosteal bone formation-a neglected determinant of bone strength. *N. Engl. J. Med.* **349**, 320–323 (2003). 12878736 10.1056/NEJMp038101

[2] Lin, Z., Fateh, A., Salem, D. M. & Intini, G. Periosteum: biology and applications in craniofacial bone regeneration. *J. Dent. Res.* **93**, 109–116 (2014). 24088412 10.1177/0022034513506445 PMC3895334

[3] Deng, R. X. et al. Periosteal CD68^*+*^ F4/80^*+*^ macrophages are mechanosensitive for cortical bone formation by secretion and activation of TGF-beta 1. *Adv. Sci.* **9**, 2103343 (2022). 10.1002/advs.202103343 PMC8787385 34854257

[4] Chang, H. & Knothe Tate, M. L. Concise review: the periosteum: tapping into a reservoir of clinically useful progenitor cells. *Stem Cells Transl. Med.* **1**, 480–491 (2012). 23197852 10.5966/sctm.2011-0056 PMC3659712

[5] Matthews, B. G. et al. Heterogeneity of murine periosteum progenitors involved in fracture healing. *eLife* **10**, e58534 (2021). 10.7554/eLife.58534 PMC7906599 33560227

[6] Jeffery, E. C., Mann, T. L. A., Pool, J. A., Zhao, Z. & Morrison, S. J. Bone marrow and periosteal skeletal stem/progenitor cells make distinct contributions to bone maintenance and repair. *Cell Stem Cell* **29**, 1547–1561.e1546 (2022). 36272401 10.1016/j.stem.2022.10.002

[7] Tsukasaki, M. et al. Periosteal stem cells control growth plate stem cells during postnatal skeletal growth. *Nat. Commun.* **13**, 4166 (2022). 35851381 10.1038/s41467-022-31592-x PMC9293991

[8] Chen, R. et al. Sfrp4 is required to maintain Ctsk-lineage periosteal stem cell niche function. *Proc. Natl. Acad. Sci. USA* **120**, e2312677120 (2023). 37931101 10.1073/pnas.2312677120 PMC10655581

[9] Debnath, S. et al. Discovery of a periosteal stem cell mediating intramembranous bone formation. *Nature* **562**, 133 (2018). 30250253 10.1038/s41586-018-0554-8 PMC6193396

[10] Ortinau, L. C. et al. Identification of functionally distinct Mx1+alpha SMA plus periosteal skeletal stem cells. *Cell Stem Cell* **25**, 784 (2019). 31809737 10.1016/j.stem.2019.11.003 PMC7055207

[11] Roelofs, A. J. et al. Identification of the skeletal progenitor cells forming osteophytes in osteoarthritis. *Ann. Rheum. Dis.* **79**, 1625–1634 (2020). 32963046 10.1136/annrheumdis-2020-218350 PMC8136618

[12] Zhu, J. et al. Endochondral repair of jawbone defects using periosteal cell spheroids. *J. Dent. Res.* **103**, 31–41 (2024). 37968792 10.1177/00220345231205273

[13] Xu, J. J. et al. PDGFR alpha reporter activity identifies periosteal progenitor cells critical for bone formation and fracture repair. *Bone Res.* **10**, 7 (2022). 10.1038/s41413-021-00176-8 PMC8786977 35075130

[14] Rindone, A. N. et al. Quantitative 3D imaging of the cranial microvascular environment at single-cell resolution. *Nat. Commun.* **12**, 6219 (2021). 34711819 10.1038/s41467-021-26455-w PMC8553857

[15] Mo, C. Y. et al. Single-cell transcriptomics of LepR-positive skeletal cells reveals heterogeneous stress-dependent stem and progenitor pools. *EMBO J.* **41**, EMBJ2021108415 (2022). 10.15252/embj.2021108415 PMC8844986 34957577

[16] Cao, Y. et al. Characterization of adult human skeletal cells in different tissues reveals a CD90^*+*^ CD34^*+*^ periosteal stem/progenitor population. *Bone* **178**, 116926 (2024). 37793499 10.1016/j.bone.2023.116926

[17] Ding, Y., Mo, C., Geng, J., Li, J. & Sun, Y. Identification of periosteal osteogenic progenitors in jawbone. *J. Dental Res.* **101**, 1101–1109 (2022). 10.1177/00220345221084200 35319300

[18] Hsiao, H. Y., Yang, C. Y., Liu, J. W., Brey, E. M. & Cheng, M. H. Periosteal osteogenic capacity depends on tissue source. *Tissue Eng. Part A* **24**, 1733–1741 (2018). 29901423 10.1089/ten.TEA.2018.0009

[19] He, F., Umrath, F., von Ohle, C., Reinert, S. & Alexander, D. Analysis of the influence of jaw periosteal cells on macrophages phenotype using an innovative horizontal coculture system. *Biomedicines* **9**, 1753 (2021). 10.3390/biomedicines9121753 PMC8698728 34944569

[20] Xu, Y. et al. Site-specific periosteal cells with distinct osteogenic and angiogenic characteristics. *Clin. Oral. Investig.* **27**, 7437–7450 (2023). 37848582 10.1007/s00784-023-05333-3

[21] Marcucio, R. S., Miclau, T. 3rd & Bahney, C. S. A shifting paradigm: transformation of cartilage to bone during bone repair. *J. Dent. Res.* **102**, 13–20 (2023). 36303415 10.1177/00220345221125401 PMC9791286

[22] Gao, B. et al. Macrophage-lineage TRAP^*+*^ cells recruit periosteum-derived cells for periosteal osteogenesis and regeneration. *J. Clin. Investig.* **129**, 2578–2594 (2019). 30946695 10.1172/JCI98857 PMC6538344

[23] Coulis, G. et al. Single-cell and spatial transcriptomics identify a macrophage population associated with skeletal muscle fibrosis. *Sci. Adv.* **9**, eadd9984 (2023). 37418531 10.1126/sciadv.add9984 PMC10328414

[24] Morse, C. et al. Proliferating SPP1/MERTK-expressing macrophages in idiopathic pulmonary fibrosis. *Eur. Respir. J.* **54**, 1802441 (2019). 10.1183/13993003.02441-2018 PMC8025672 31221805

[25] Fabre, T. et al. Identification of a broadly fibrogenic macrophage subset induced by type 3 inflammation. *Sci. Immunol.* **8**, eadd8945 (2023). 37027478 10.1126/sciimmunol.add8945

[26] Hulsmans, M. et al. Recruited macrophages elicit atrial fibrillation. *Science* **381**, 231–239 (2023). 37440641 10.1126/science.abq3061 PMC10448807

[27] Nakamura, K. et al. The periosteum provides a stromal defence against cancer invasion into the bone. *Nature* **634**, 474–481 (2024). 39169177 10.1038/s41586-024-07822-1

[28] Liu, R. et al. Mechanosensitive protein polycystin-1 promotes periosteal stem/progenitor cells osteochondral differentiation in fracture healing. *Theranostics* **14**, 2544–2559 (2024). 38646641 10.7150/thno.93269 PMC11024844

[29] Moses, L. & Pachter, L. Museum of spatial transcriptomics. *Nat. Methods* **19**, 534–546 (2022). 35273392 10.1038/s41592-022-01409-2

[30] Trombelli, L., Pramstraller, M., Severi, M., Simonelli, A. & Farina, R. Peri-implant tissue conditions at implants treated with sub-periosteal peri-implant augmented layer technique: a retrospective case series. *Clin. Oral. Implants Res.* **31**, 992–1001 (2020). 32781494 10.1111/clr.13646

[31] Severi, M. et al. Effect of lateral bone augmentation procedures in correcting peri-implant bone dehiscence and fenestration defects: a systematic review and network meta-analysis. *Clin. Implant Dent. Relat. Res.* **24**, 251–264 (2022). 35316573 10.1111/cid.13078 PMC9315147

[32] Deng, C., Yi, Z., Xiong, C., Man, Y. & Qu, Y. Using the intact periosteum for horizontal bone augmentation of peri-implant defects: a retrospective cohort study. *Br. J. Oral. Maxillofac. Surg.* **60**, 1325–1331 (2022). 36357244 10.1016/j.bjoms.2022.09.012

[33] Liu, Y., Lan, D., Gao, J., Deng, C. & Man, Y. Guided bone regeneration for peri-implant augmentation: a retrospective study comparing two surgical techniques with a mean follow-up of 26 months. *Clin. Oral. Implants Res.* **35**, 573–584 (2024). 38467593 10.1111/clr.14254

[34] Mouraret, S. et al. The potential for vertical bone regeneration via maxillary periosteal elevation. *J. Clin. Periodontol.* **41**, 1170–1177 (2014). 25229322 10.1111/jcpe.12310

[35] Butler, A., Hoffman, P., Smibert, P., Papalexi, E. & Satija, R. Integrating single-cell transcriptomic data across different conditions, technologies, and species. *Nat. Biotechnol.* **36**, 411–420 (2018). 29608179 10.1038/nbt.4096 PMC6700744

[36] Colnot, C. Skeletal cell fate decisions within periosteum and bone marrow during bone regeneration. *J. Bone Miner. Res.* **24**, 274–282 (2009). 18847330 10.1359/jbmr.081003 PMC3276357

[37] Perrin, S. et al. Single-nucleus transcriptomics reveal the differentiation trajectories of periosteal skeletal/stem progenitor cells in bone regeneration. *eLife* **13**, RP92519 (2024). 10.7554/eLife.92519 PMC11623931 39642053

[38] Dondossola, E. & Friedl, P. Host responses to implants revealed by intravital microscopy. *Nat. Rev. Mater.* **7**, 6–22 (2022).

[39] Padmanabhan, J. et al. Allometrically scaling tissue forces drive pathological foreign-body responses to implants via Rac2-activated myeloid cells. *Nat. Biomed. Eng.* **7**, 1419–1436 (2023). 10.1038/s41551-023-01091-5 PMC10651488 37749310

[40] Halasi, M. et al. Melanocyte-secreted fibromodulin constrains skin inflammation in mice injected with lupus serum. *ClinImmunol.* **241**, 109055 (2022). 10.1016/j.clim.2022.109055 35640789

[41] Reeves, J. et al. Rejuvenating aged osteoprogenitors for bone repair. *eLife* **13**, RP104068 (2024). 10.7554/eLife.104068 PMC11655067 39692737

[42] Jeong, Y. et al. Identification of LRP1^*+*^CD13^*+*^ human periosteal stem cells that require LRP1 for bone repair. *JCI Insight* **9**, e173831 (2024). 10.1172/jci.insight.173831 PMC11601900 39405183

[43] Li, Y. et al. EGR1 promotes craniofacial bone regeneration via activation of ALPL⁺PDGFD⁺ periosteal stem cells. *Adv. Sci.* **12**, e10243 (2025). 10.1002/advs.202410243 PMC12376705 40650643

[44] Qiu, X. et al. Reversed graph embedding resolves complex single-cell trajectories. *Nat. Methods* **14**, 979–982 (2017). 28825705 10.1038/nmeth.4402 PMC5764547

[45] Vi, L. et al. Macrophage cells secrete factors including LRP1 that orchestrate the rejuvenation of bone repair in mice. *Nat. Commun.* **9**, 5191 (2018). 30518764 10.1038/s41467-018-07666-0 PMC6281653

[46] Hu, C. et al. Dissecting the microenvironment around biosynthetic scaffolds in murine skin wound healing. *Sci. Adv.* **7**, eabf0787 (2021). 10.1126/sciadv.abf0787 PMC8153724 34039601

[47] Qi, J. et al. Single-cell and spatial analysis reveal interaction of FAP^*+*^ fibroblasts and SPP1(+) macrophages in colorectal cancer. *Nat. Commun.* **13**, 1742 (2022). 35365629 10.1038/s41467-022-29366-6 PMC8976074

[48] Yang, Y. et al. Tracing immune cells around biomaterials with spatial anchors during large-scale wound regeneration. *Nat. Commun.* **14**, 5995 (2023). 37752124 10.1038/s41467-023-41608-9 PMC10522601

[49] Hou, J. et al. TREM2 sustains macrophage-hepatocyte metabolic coordination in nonalcoholic fatty liver disease and sepsis. *J. Clin. Invest.* **131**, e135197 (2021). 10.1172/JCI135197 PMC7880419 33586673

[50] Ramachandran, P. et al. Resolving the fibrotic niche of human liver cirrhosis at single-cell level. *Nature* **575**, 512–518 (2019). 31597160 10.1038/s41586-019-1631-3 PMC6876711

[51] Cable, D. M. et al. Robust decomposition of cell type mixtures in spatial transcriptomics. *Nat. Biotechnol.* **40**, 517–526 (2022). 33603203 10.1038/s41587-021-00830-w PMC8606190

[52] Rajachandran, S. et al. Dissecting the spermatogonial stem cell niche using spatial transcriptomics. *Cell Rep.* **42**, 112737 (2023). 37393620 10.1016/j.celrep.2023.112737 PMC10530051

[53] Moncada, R. et al. Integrating microarray-based spatial transcriptomics and single-cell RNA-seq reveals tissue architecture in pancreatic ductal adenocarcinomas. *Nat. Biotechnol.* **38**, 333–342 (2020). 31932730 10.1038/s41587-019-0392-8

[54] Konieczny, P. et al. Interleukin-17 governs hypoxic adaptation of injured epithelium. *Science* **377**, eabg9302 (2022). 35709248 10.1126/science.abg9302 PMC9753231

[55] Castillo, R. L. et al. Spatial transcriptomics stratifies psoriatic disease severity by emergent cellular ecosystems. *Sci. Immunol.* **8**, eabq7991 (2023). 37267384 10.1126/sciimmunol.abq7991 PMC10502701

[56] Ji, A. L. et al. Multimodal analysis of composition and spatial architecture in human squamous cell carcinoma. *Cell* **182**, 497–514.e422 (2020). 32579974 10.1016/j.cell.2020.05.039 PMC7391009

[57] Elosua-Bayes, M., Nieto, P., Mereu, E., Gut, I. & Heyn, H. SPOTlight: seeded NMF regression to deconvolute spatial transcriptomics spots with single-cell transcriptomes. *Nucleic Acids Res.* **49**, e50 (2021). 33544846 10.1093/nar/gkab043 PMC8136778

[58] Tanevski, J., Flores, R. O. R., Gabor, A., Schapiro, D. & Saez-Rodriguez, J. Explainable multiview framework for dissecting spatial relationships from highly multiplexed data. *Genome Biol.* **23**, 97 (2022). 35422018 10.1186/s13059-022-02663-5 PMC9011939

[59] Almet, A. A., Cang, Z., Jin, S. & Nie, Q. The landscape of cell-cell communication through single-cell transcriptomics. *Curr. Opin. Syst. Biol.* **26**, 12–23 (2021). 33969247 10.1016/j.coisb.2021.03.007 PMC8104132

[60] Jin, S. et al. Inference and analysis of cell-cell communication using CellChat. *Nat. Commun.* **12**, 1088 (2021). 33597522 10.1038/s41467-021-21246-9 PMC7889871

[61] Cang, Z. et al. Screening cell-cell communication in spatial transcriptomics via collective optimal transport. *Nat. Methods* **20**, 218–228 (2023). 36690742 10.1038/s41592-022-01728-4 PMC9911355

[62] Rosin, J. M., Sinha, S., Biernaskie, J. & Kurrasch, D. M. A subpopulation of embryonic microglia respond to maternal stress and influence nearby neural progenitors. *Dev. Cell* **56**, 1326–1345.e1326 (2021). 33887203 10.1016/j.devcel.2021.03.018

[63] Xiong, X. et al. Cannabis suppresses antitumor immunity by inhibiting JAK/STAT signaling in T cells through CNR2. *Signal Transduct. Target Ther.* **7**, 99 (2022). 35383142 10.1038/s41392-022-00918-y PMC8983672

[64] Eun, J. W. et al. Cancer-associated fibroblast-derived secreted phosphoprotein 1 contributes to resistance of hepatocellular carcinoma to sorafenib and lenvatinib. *Cancer Commun.* **43**, 455–479 (2023). 10.1002/cac2.12414 PMC10091107 36919193

[65] Yang, W. et al. Succinic acid-induced macrophage endocytosis promotes extracellular vesicle-based integrin Beta1 transfer accelerating fibroblast activation and sepsis-associated pulmonary fibrosis. *Adv. Sci.* **12**, e07411 (2025). 10.1002/advs.202507411 PMC12631846 40898357

[66] Duchamp de Lageneste, O. et al. Periosteum contains skeletal stem cells with high bone regenerative potential controlled by Periostin. *Nat. Commun.* **9**, 773 (2018). 29472541 10.1038/s41467-018-03124-z PMC5823889

[67] Kalamajski, S. & Oldberg, Å Fibromodulin binds collagen type I via Glu-353 and Lys-355 in leucine-rich repeat 11. *J. Biol. Chem.* **282**, 26740–26745 (2007). 17623650 10.1074/jbc.M704026200

[68] Li, C. J. et al. Long noncoding RNA Bmncr regulates mesenchymal stem cell fate during skeletal aging. *J. Clin. Invest.* **128**, 5251–5266 (2018). 30352426 10.1172/JCI99044 PMC6264619

[69] Qian, H., Xiao, Y. & Bartold, P. M. Immunohistochemical localization and expression of fibromodulin in adult rat periodontium and inflamed human gingiva. *Oral. Dis.* **10**, 233–239 (2004). 15196146 10.1111/j.1601-0825.2004.00996.x

[70] Matsushita, Y. et al. The fate of early perichondrial cells in developing bones. *Nat. Commun.* **13**, 7319 (2022). 36443296 10.1038/s41467-022-34804-6 PMC9705540

[71] Kim, Y. S. et al. Different molecular features of epithelioid and giant cells in foreign body reaction identified by single-cell RNA sequencing. *J. Invest. Dermatol.* **142**, 3232–3242.e3216 (2022). 35853485 10.1016/j.jid.2022.06.014

[72] Wang, K. X. & Denhardt, D. T. Osteopontin: role in immune regulation and stress responses. *Cytokine Growth Factor Rev.* **19**, 333–345 (2008). 18952487 10.1016/j.cytogfr.2008.08.001

[73] Nilsson, S. K. et al. Osteopontin, a key component of the hematopoietic stem cell niche and regulator of primitive hematopoietic progenitor cells. *Blood* **106**, 1232–1239 (2005). 15845900 10.1182/blood-2004-11-4422

[74] Guidi, N. et al. Osteopontin attenuates aging-associated phenotypes of hematopoietic stem cells. *EMBO J.* **36**, 840–853 (2017). 28254837 10.15252/embj.201694969 PMC5376966

[75] Coombes, J. D. et al. Osteopontin neutralisation abrogates the liver progenitor cell response and fibrogenesis in mice. *Gut* **64**, 1120–1131 (2015). 24902765 10.1136/gutjnl-2013-306484 PMC4487727

[76] Yue, B. et al. SPP1 induces idiopathic pulmonary fibrosis and NSCLC progression via the PI3K/Akt/mTOR pathway. *Respir. Res.* **25**, 362 (2024). 39369217 10.1186/s12931-024-02989-7 PMC11456247

[77] Qin, W., Cao, L. & Massey, I. Y. Role of PI3K/Akt signaling pathway in cardiac fibrosis. *Mol. Cell. Biochem.* **476**, 4045–4059 (2021). 34244974 10.1007/s11010-021-04219-w

[78] Li, X. et al. Duvelisib attenuates bleomycin-induced pulmonary fibrosis via inhibiting the PI3K/Akt/mTOR signalling pathway. *J. Cell. Mol. Med.* **27**, 422–434 (2023). 36651446 10.1111/jcmm.17665 PMC9889612

[79] Tanaka, Y. et al. Suppression of AKT-mTOR signal pathway enhances osteogenic/dentinogenic capacity of stem cells from apical papilla. *Stem cell Res. Ther.* **9**, 334 (2018). 30486861 10.1186/s13287-018-1077-9 PMC6264601

[80] Lou, F. et al. Activation of autophagy mediated by PI3K/Akt/mTOR signalling cascade alleviates impaired adipose-derived stem cell osteogenesis in a diabetic microenvironment. *Br. J. Pharmacol.* **182**, 3522–3538 (2025). 40170369 10.1111/bph.70016

[81] Pantovic, A. et al. Coordinated time-dependent modulation of AMPK/Akt/mTOR signaling and autophagy controls osteogenic differentiation of human mesenchymal stem cells. *Bone* **52**, 524–531 (2013). 23111315 10.1016/j.bone.2012.10.024

