# Supplementary material for: Multi-omic analysis of jaw-bone periosteum identifies the role of macrophage-derived SPP1 in Ctsk+Fmod+ periosteal cells
Source: Bone Res. 2026 Sep 22;14:101. doi: 10.1038/s41413-026-00585-7 (PMC13597513; doi:10.1038/s41413-026-00585-7)
Supplement: Supplementary file 1 [file 41413_2026_585_MOESM1_ESM.pdf]

Supplementary Materials for

Multi-omic analysis of jaw-bone periosteum identifies the role of macrophage-derived SPP1 in *Ctsk+Fmod+* periosteal cells

Zumu Yi *et al.*

*Corresponding author. huchen_scu@scu.edu.cn (Chen Hu); manyi780203@126.com (Yi Man).

This PDF file includes:

Figs. S1 to S20

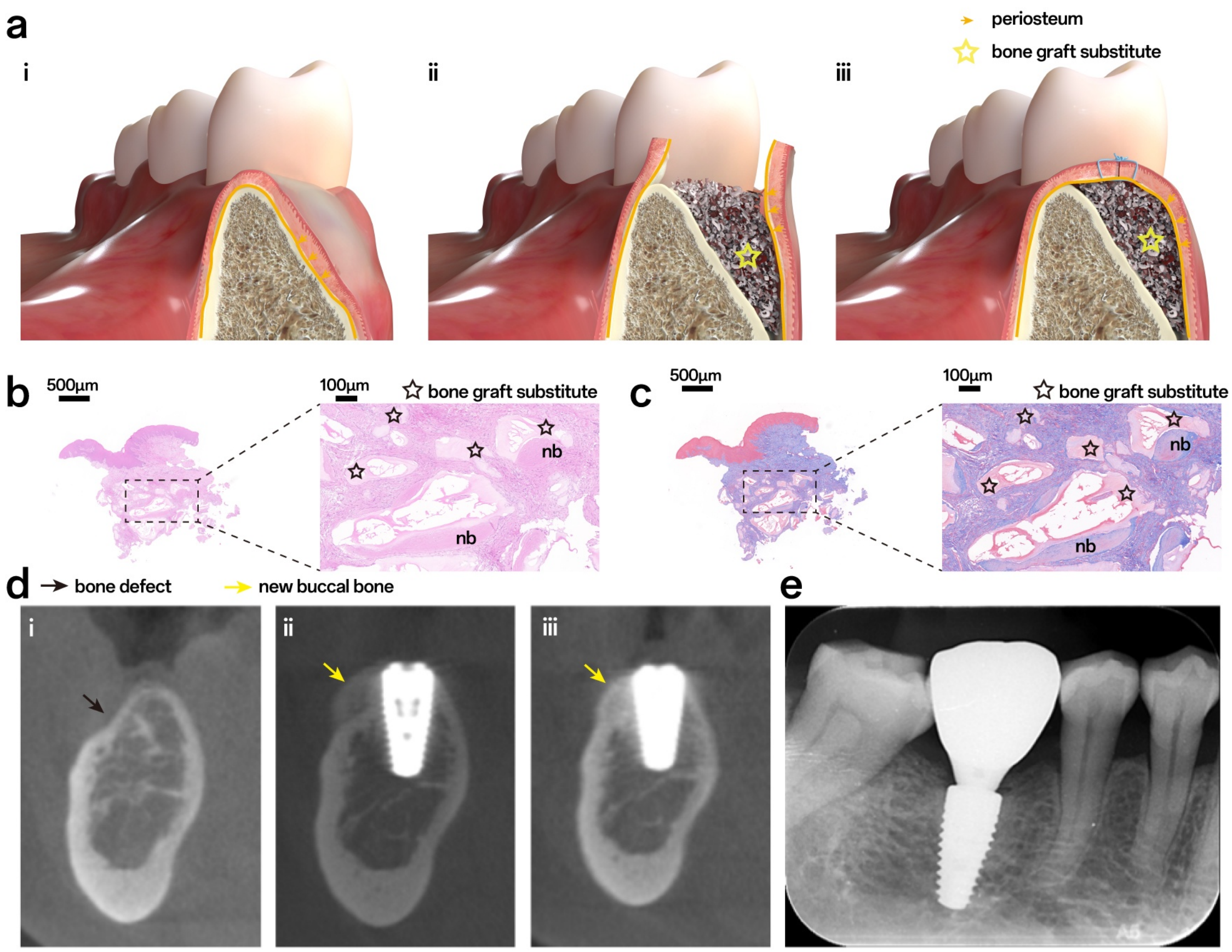

Fig. S1.

Clinical procedures of modified sub-periosteal guided bone regeneration (GBR) and the histological analysis of newly formed tissue harvested 6 months post-surgery. **(a)** The schematic diagram for the surgical procedures of modified GBR. i) The buccal bone defect of the alveolar ridge; ii) A full-thickness mucoperiosteal flap was elevated and extended beyond the defect area. Then the bone substitute was filled into the space between the bone defect and the intact periosteum; iii) The surgical area was closed. Stars, bone graft substitute. Yellow arrows, periosteum. Representative H&E; **(b)** and Masson staining **(c)** images of samples collected 6 months after surgery from a male patient aged 46. New bone formed around the bone graft substitute. Stars, bone graft substitute. Abbreviations: nb, new bone. **(d)** The Cone-beam computed tomography (CBCT) record of modified GBR case of a male patient aged 52. i) The coronal view of CBCT before the surgery: the buccal bone showed a concave shape at the crestal area (white arrow); ii) The coronal view of CBCT after implantation and the application of modified GBR: sufficient buccal bone surrounded the implant (yellow arrow); iii) The coronal view of CBCT 6-month post-surgery: the stable buccal bone surrounded the implant (yellow arrow). **(e)** The periapical film of the implant 3 months after the final restoration.

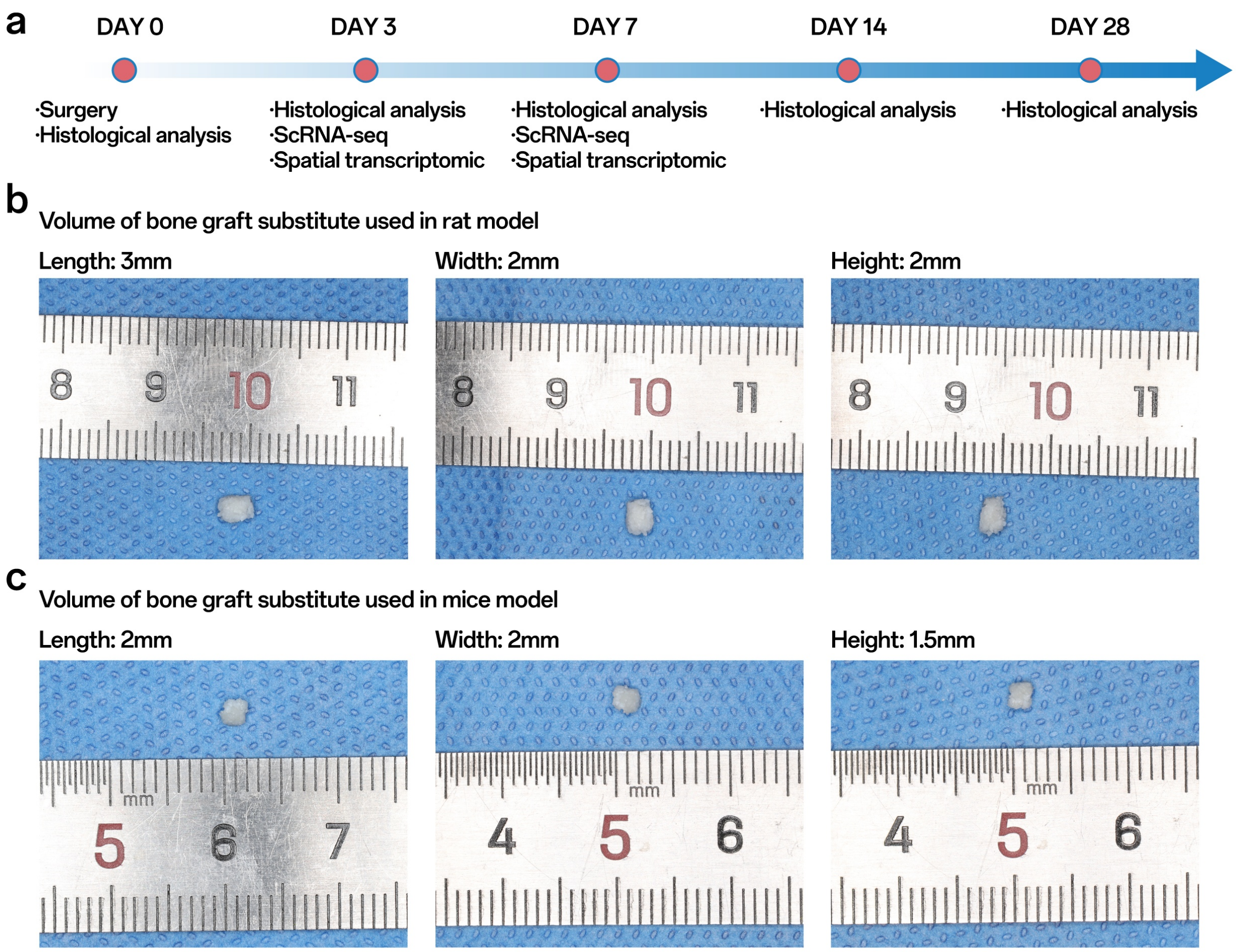

Fig. S2.

Standardization of graft substitute volume in the modified GBR model (a) Workflow of rat subperiosteal bone grafting model establishment and sample analysis. **(b)** Representative measurement of the graft substitute used in the rat model, with a standardized volume of 3 × 2 × 2 mm. **(c)** Representative measurement of the graft substitute used in the mouse model, with a standardized volume of 2 × 2 × 1.5 mm.

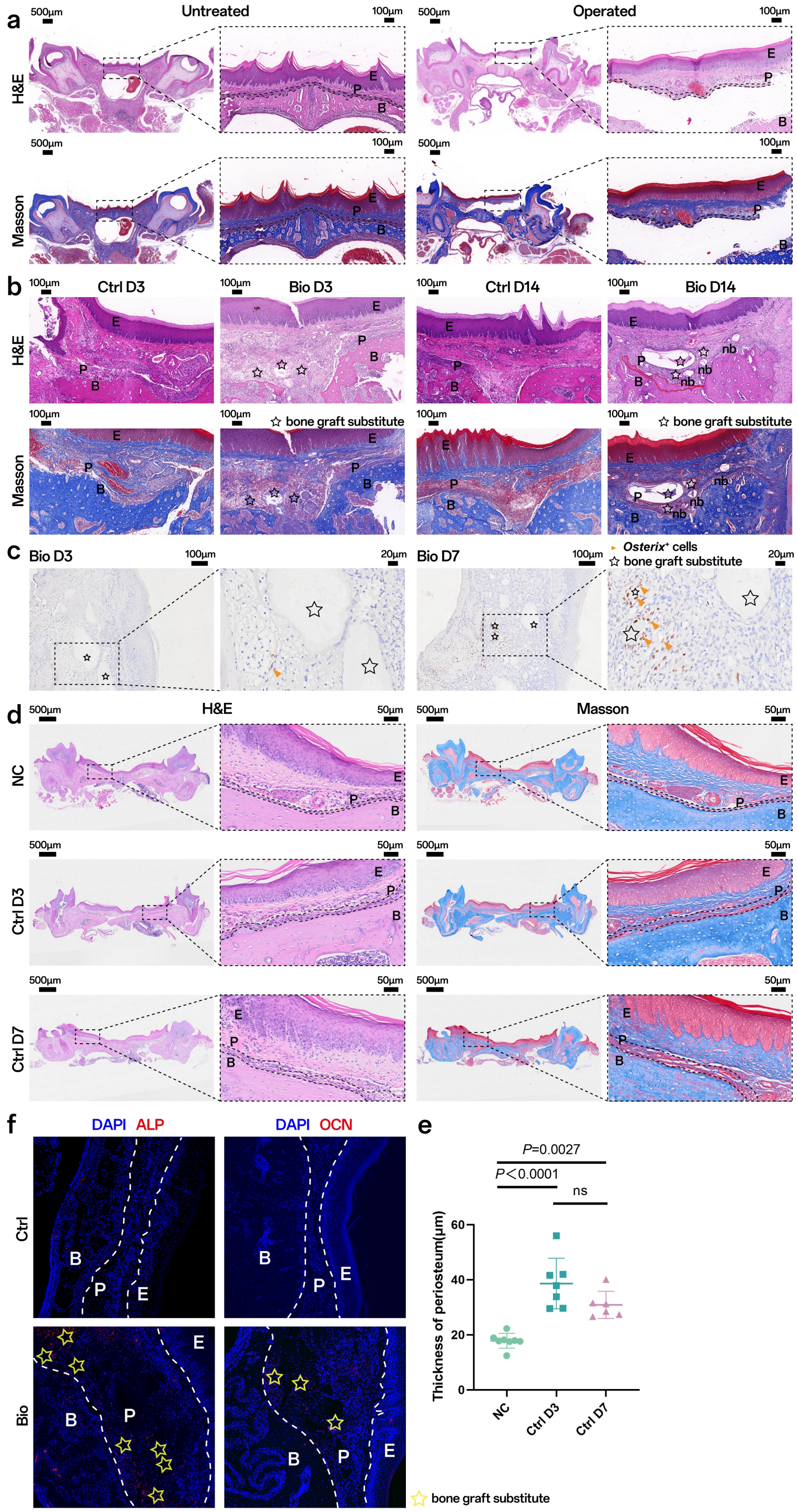
a
Untreated
Operated
H&E
Masson
500μm
100μm
E
P
B
b
Ctrl D3
Bio D3
Ctrl D14
Bio D14
nb
☆ bone graft substitute
c
Bio D3
Bio D7
20μm
Osterix+ cells
☆ bone graft substitute
d
H&E
Masson
NC
Ctrl D3
Ctrl D7
50μm
f
DAPI ALP
DAPI OCN
Ctrl
Bio
☆ bone graft substitute
e
P=0.0027
P<0.0001
ns
Thickness of periosteum(μm)
60
40
20
0
NC
Ctrl D3
Ctrl D7

Fig. S3.

Histological analysis of rat modified GBR model. **(a)** Representative H&E; and Masson staining images of untreated and operated group. The periosteum was completely elevated from the bony surface in the operated group. **(b)** Representative H&E; and Masson staining images of Ctrl and Bio group on Day3, Day14. At post-surgical Day3, more blood cells and fibrin networks could be found in Bio group. New bone began to occur at Day14 in Bio group near the bone graft. Red dotted line, new bone area. **(c)** Immunohistochemical staining of the *osterix*+ cells in the D3Bio and D7Bio. Stars, bone graft. Yellow arrows, *osterix*+ cells. **(d)** Representative H&E; and Masson staining images of no control (NC) group and operated (Ctrl) group and the quantitative analyses of periosteal thickness between different groups **(e)**. ns, not significant. **(f)** Representative confocal images of sample sections from Ctrl and Bio group at Day3. Notice the location of ALP and OCN expressions. Stars, bone graft. Abbreviations: E, epithelium; P, periosteum; B, bone; nb, new bone.

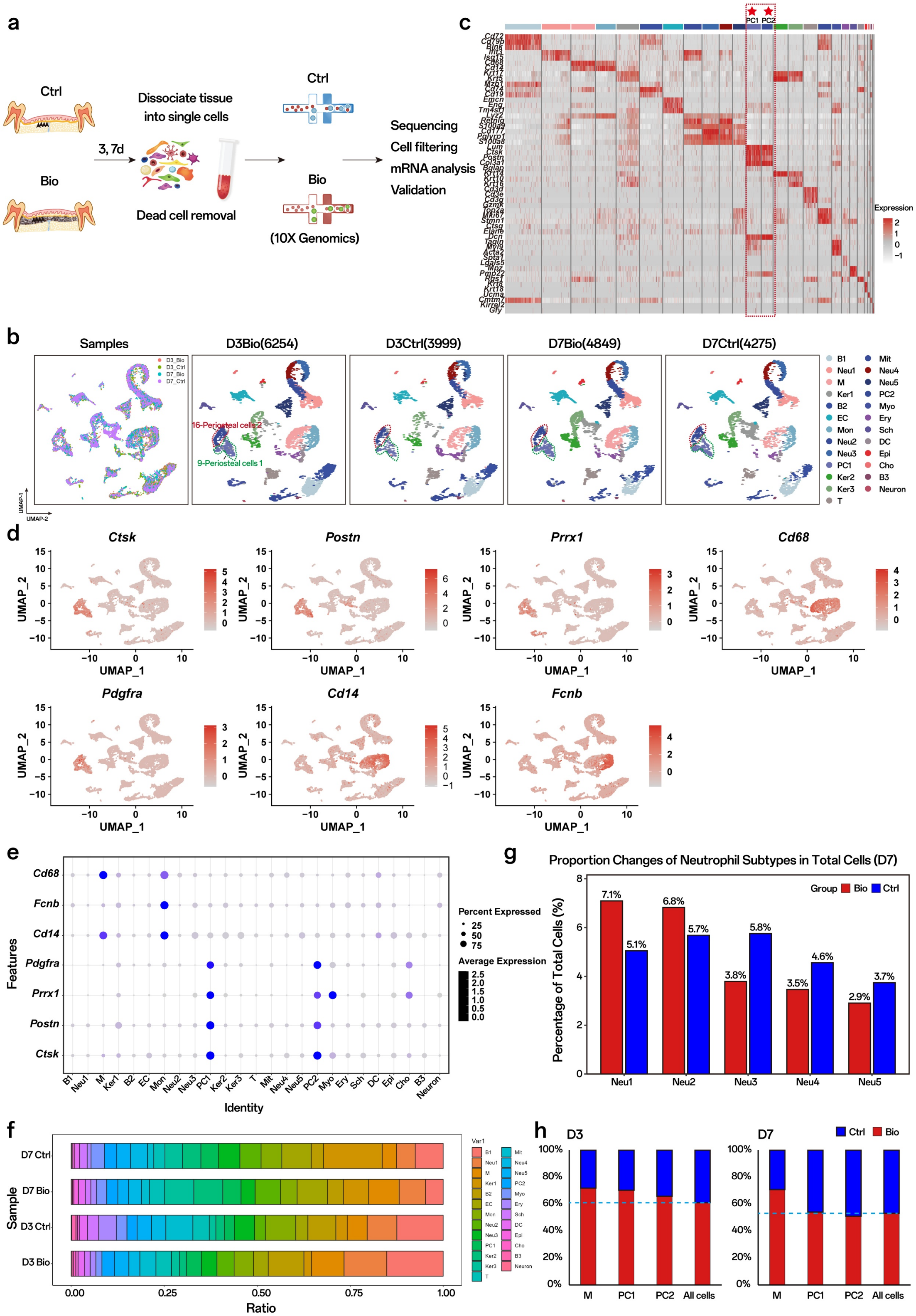

a
Ctrl
Bio
3, 7d
Dissociate tissue into single cells
Dead cell removal
(10X Genomics)
Sequencing
Cell filtering
mRNA analysis
Validation
b
Samples
D3Bio(6254)
D3Ctrl(3999)
D7Bio(4849)
D7Ctrl(4275)
16-Periosteal cells 2
9-Periosteal cells 1
c
PC1
PC2
Expression
d
Ctsk
Postn
Prrx1
Cd68
Pdgfra
Cd14
Fcnb
UMAP_1
UMAP_2
e
Features
Identity
Percent Expressed
Average Expression
f
Sample
Ratio
g
Proportion Changes of Neutrophil Subtypes in Total Cells (D7)
Percentage of Total Cells (%)
Group Bio Ctrl
h
D3
D7
M
PC1
PC2
All cells

Fig. S4.

Overview of the scRNA-seq analysis between different groups. (a) Workflow of scRNA-seq. **(b)** UMAP plots of cells at all time points and at each time point of different group. The green circle indicates periosteal cells 1 and the red circle indicates periosteal cells 2. B-B cells, Neu-Neutrophils, M-Macrophages, Ker-Keratinocytes, EC-Endothelial cells, Mon-Monocytes, PC-periosteal cells, T-T cells, Mit-Mitotic cells, Myo-Myofibroblasts, Ery-Erythrocytes, Sch-Schwann cells, DC-Dendritic cells, Epi-Epithelium cells, Cho-Chondrocytes, Neuron-Neurons. **(c)** Heatmap showed top marker genes for each cluster. The stars showed the periosteal cells. UMAP plots **(d)** and Dot plot **(e)** showing expression levels of selected known marker genes. Bar plots showing proportion of all clusters in different groups **(f)** and selected clusters, including neutrophils **(g)** and macrophages and PCs **(h)** in different groups at different timepoints.

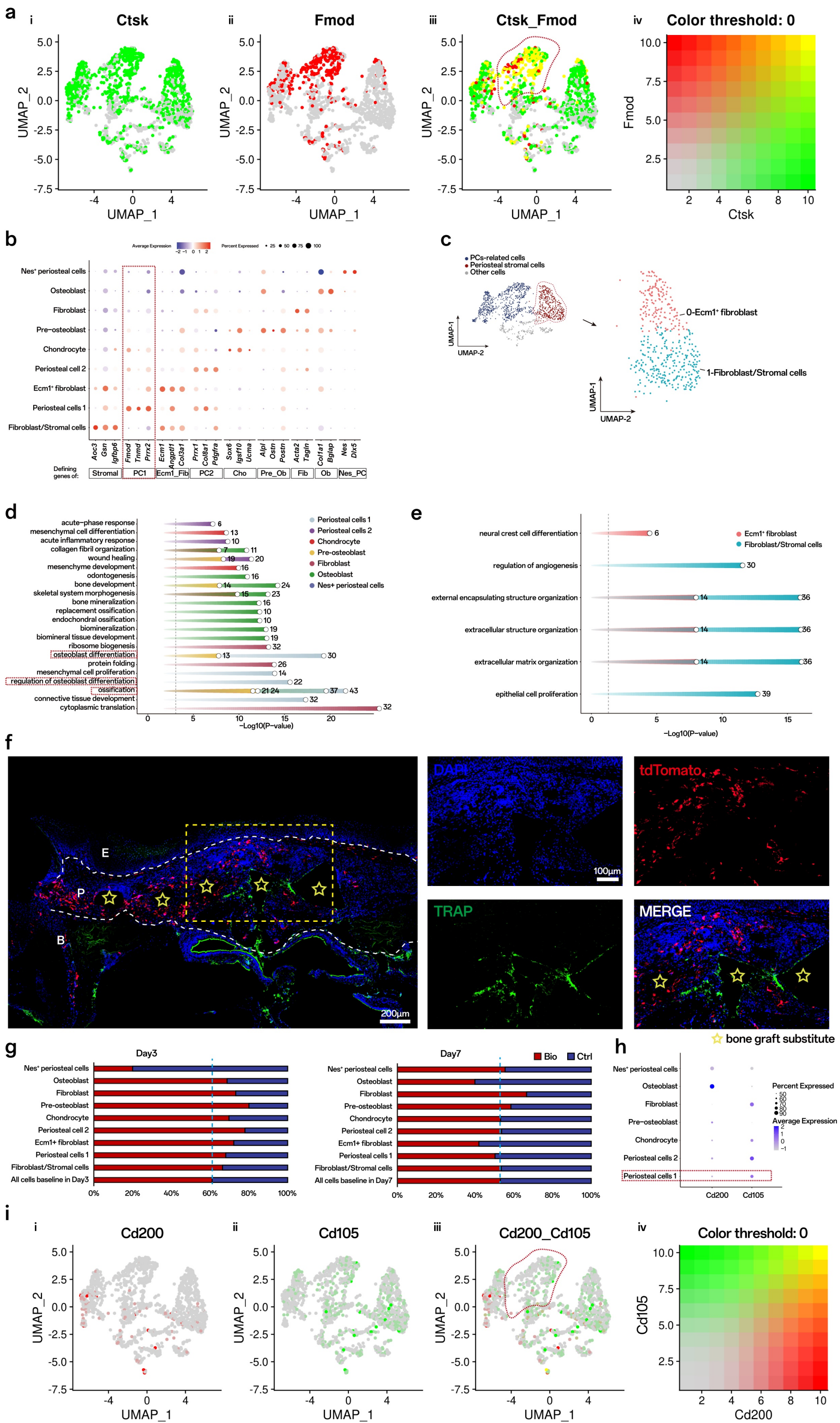
a
Ctsk
Fmod
Ctsk_Fmod
Color threshold: 0
UMAP_1
UMAP_2
b
c
PCs-related cells
Periosteal stromal cells
Other cells
0-Ecm1⁺ fibroblast
1-Fibroblast/Stromal cells
d
e
neural crest cell differentiation
regulation of angiogenesis
external encapsulating structure organization
extracellular structure organization
extracellular matrix organization
epithelial cell proliferation
-Log10(P-value)
f
DAPI
tdTomato
TRAP
MERGE
100μm
200μm
bone graft substitute
g
Day3
Day7
Bio
Ctrl
h
Cd200
Cd105
Periosteal cells 1
i
Cd200
Cd105
Cd200_Cd105
Color threshold: 0

Fig. S5

Further analysis of periosteal cells. **(a)** Blended feature plots showing co-expression of Ctsk and Fmod. (i) The expression of Ctsk; (ii) The expression of Fmod; (iii) The co-expression of Ctsk and Fmod; (iv) The expression level of Fmod (red) and Ctsk (green). These two markers were colocalized in periosteal cells 1 (*Ctsk+Fmod+* PCs). **(b)** Dot plots showing expression percent and average expression of markers in subtypes of PCs. **(c)** UMAP plots showing subclustering results of PCs. **(d)** GO analysis of subclusters of PSCs-related cells. The number in the circle showed the Gene count. GO analysis unveiled that the periosteal cell 1 subcluster was associated with ossification, regulation of osteoblast differentiation and osteoblast differentiation (red boxes). **(e)** GO analysis of subclusters of periosteal stromal cells. The number in the circle showed the Gene count. **(f)** Representative confocal images of sample sections from Bio group. Stars, bone graft substitute. Abbreviations: E, epithelium; P, periosteum; B, bone. **(g)** Bar plots showing proportion of PCs-related cells in different group on Day3 and Day7. **(h)** Dot plots showing expression percentage and average expression of Cd200 and Cd105 in subtypes of PCs. The red box indicates the expression pattern of periosteal cells 1 (*Ctsk+Fmod+* PCs). **(i)** Blended feature plots showing co-expression of Cd200 and Cd105. (i) The expression of Cd200; (ii) The expression of Cd105; (iii) The co-expression of Cd200 and Cd105; (iv) The expression level of Cd200 (red) and Cd105 (green).

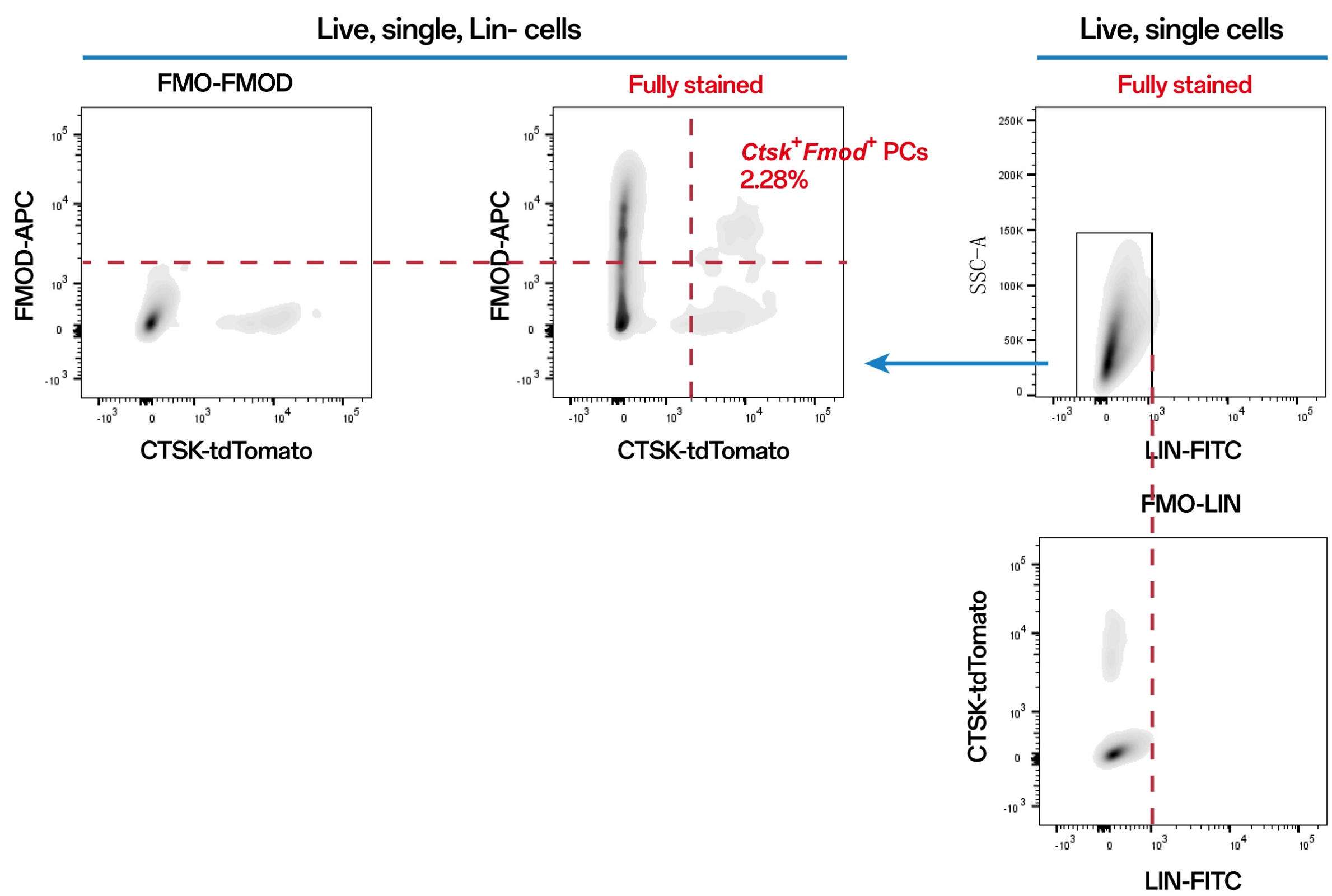

Fig. S6

Gating scheme for *Ctsk+Fmod+* PCs from mice using an optimized fixation and permeabilization-based staining protocol. Multicolor flow cytometry gating strategy to isolate *Ctsk+Fmod+* PCs from single cell suspension. Representative flow cytometry plots of periosteal cells of *Ctsk-cre; Rosa26-tdTomato* mice. FMO-Fluorescence Minus One. Lin-*CD45+CD31+Ter119+*.

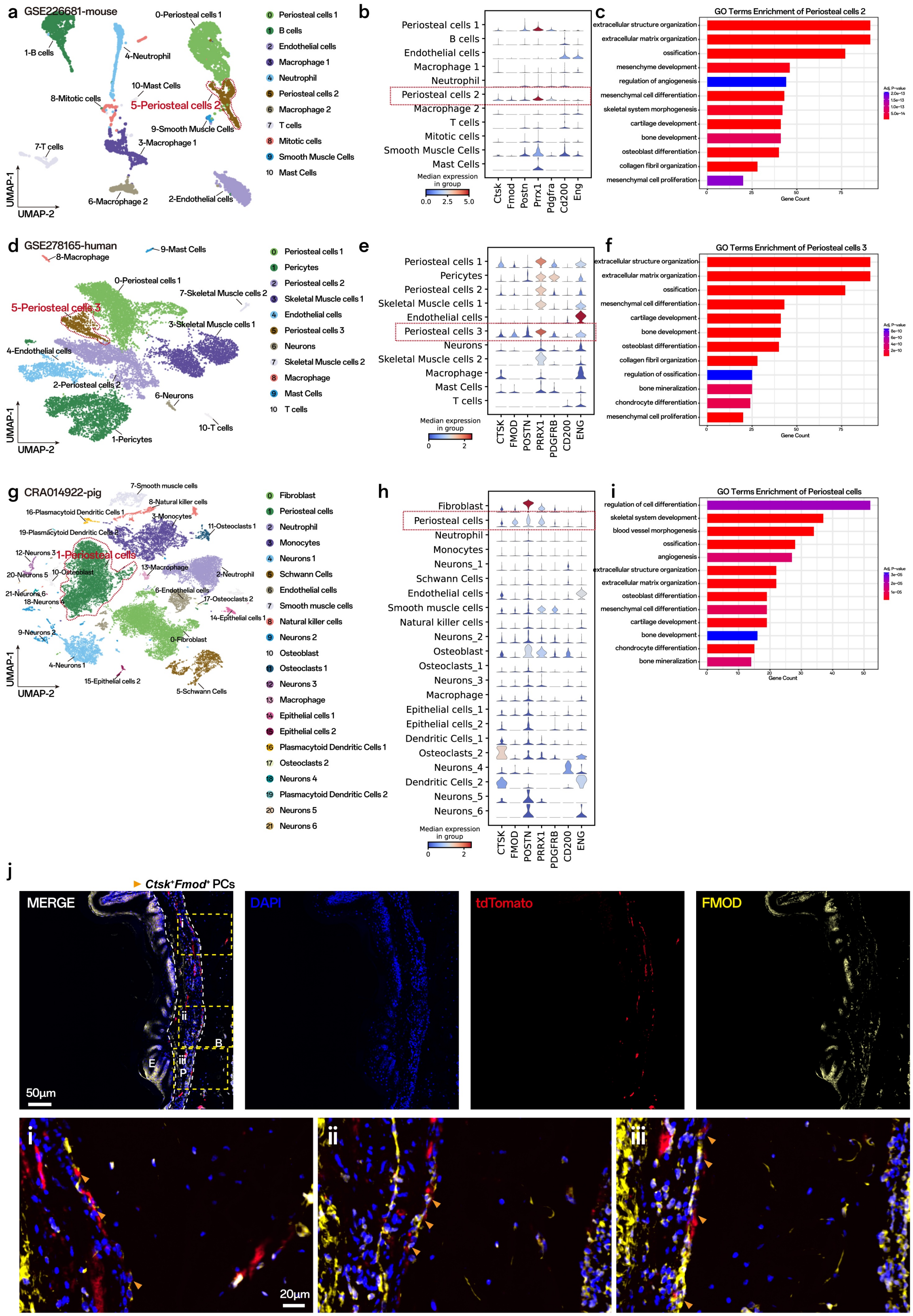
a GSE226681-mouse
b
c GO Terms Enrichment of Periosteal cells 2
d GSE278165-human
e
f GO Terms Enrichment of Periosteal cells 3
g CRA014922-pig
h
i GO Terms Enrichment of Periosteal cells
j Ctsk+Fmod+ PCs
MERGE
DAPI
tdTomato
FMOD
50μm
20μm

Fig. S7.

Analysis of steady-state periosteum. (a) UMAP plots showing clustering results of GSE226681 (mouse). **(b)** Violin plots showing average expression of markers in different cell clusters. **(c)** GO analysis of periosteal cells 2. **(d)** UMAP plots showing clustering results of GSE278165 (human). **(e)** Violin plots showing average expression of markers in different cell clusters. **(f)** GO analysis of periosteal cells 3. **(g)** UMAP plots showing clustering results of CRA014922 (pig). **(h)** Violin plots showing average expression of markers in different cell clusters. **(i)** GO analysis of periosteal cells. **(j)** Representative confocal images of sample sections from no control (NC) group. The periosteum contained *Ctsk+Fmod+* PCs. Yellow arrows, *Ctsk+Fmod+* PCs. Abbreviations: E, epithelium; P, periosteum; B, bone.

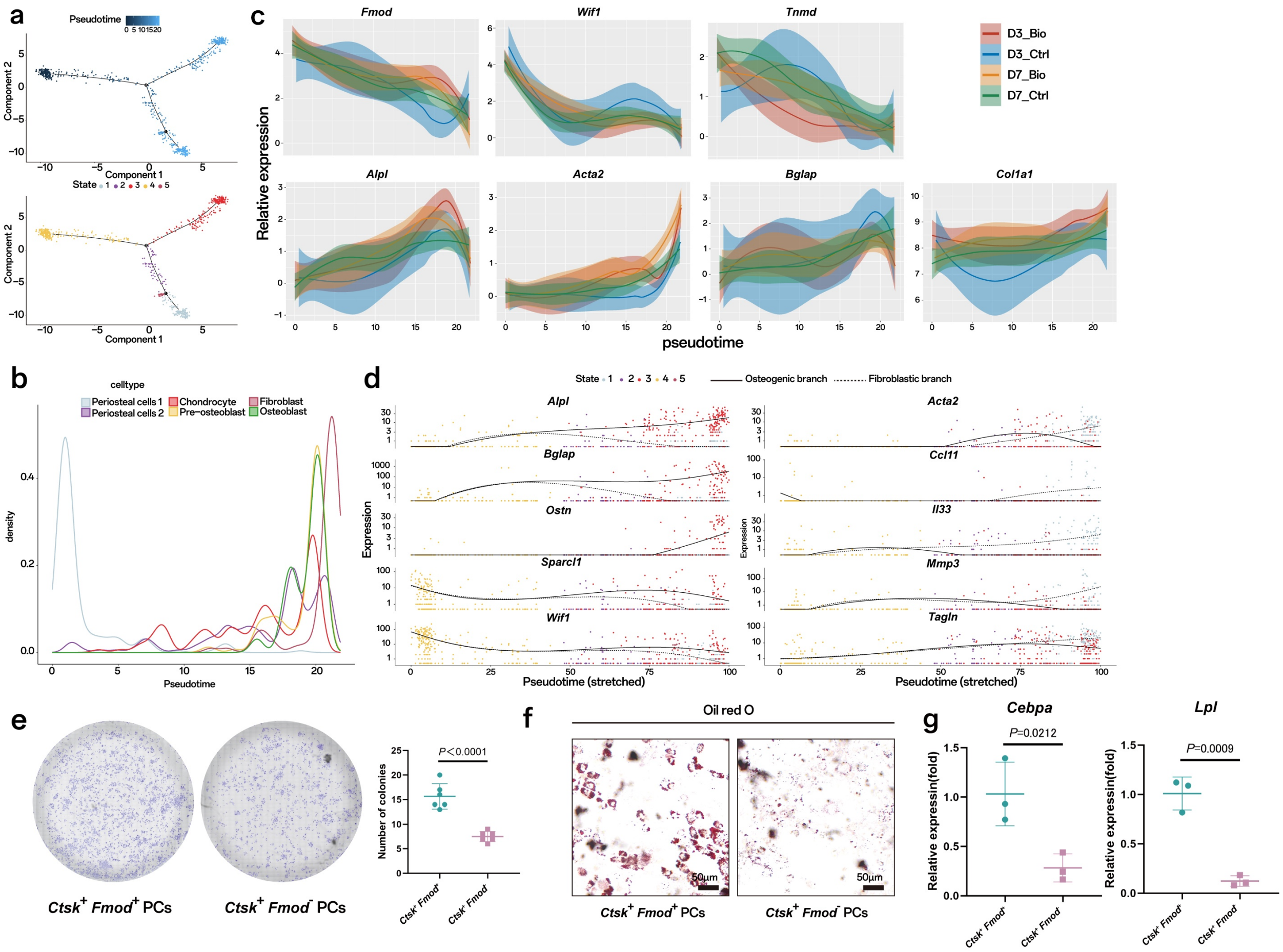

Fig. S8

Analysis of PCs transition states. (a) Trajectory reconstruction of PCs based on a pseudo-temporal order from left to right. **(b)** Plot of cell density over Pseudotime. **(c)** Two-dimensional plots with the dynamic expression of genes related to *Ctsk+Fmod+* PCs, osteogenic or fibroblastic markers in different groups, along with the Pseudotime. **(d)** Relative gene expression dynamics of genes in cells on osteogenic and fibroblastic trajectory. **(e)** Colony formation of *Ctsk+Fmod+* PCs at Day14 via crystal violet staining. **(f)** Oil red O staining of *Ctsk+Fmod+* PCs and *Ctsk+Fmod-* PCs on Day21. **(g)** qPCR analysis of the mRNA levels of *Cebpa* and *Lpl* of *Ctsk+Fmod+* PCs and *Ctsk+Fmod-* PCs.

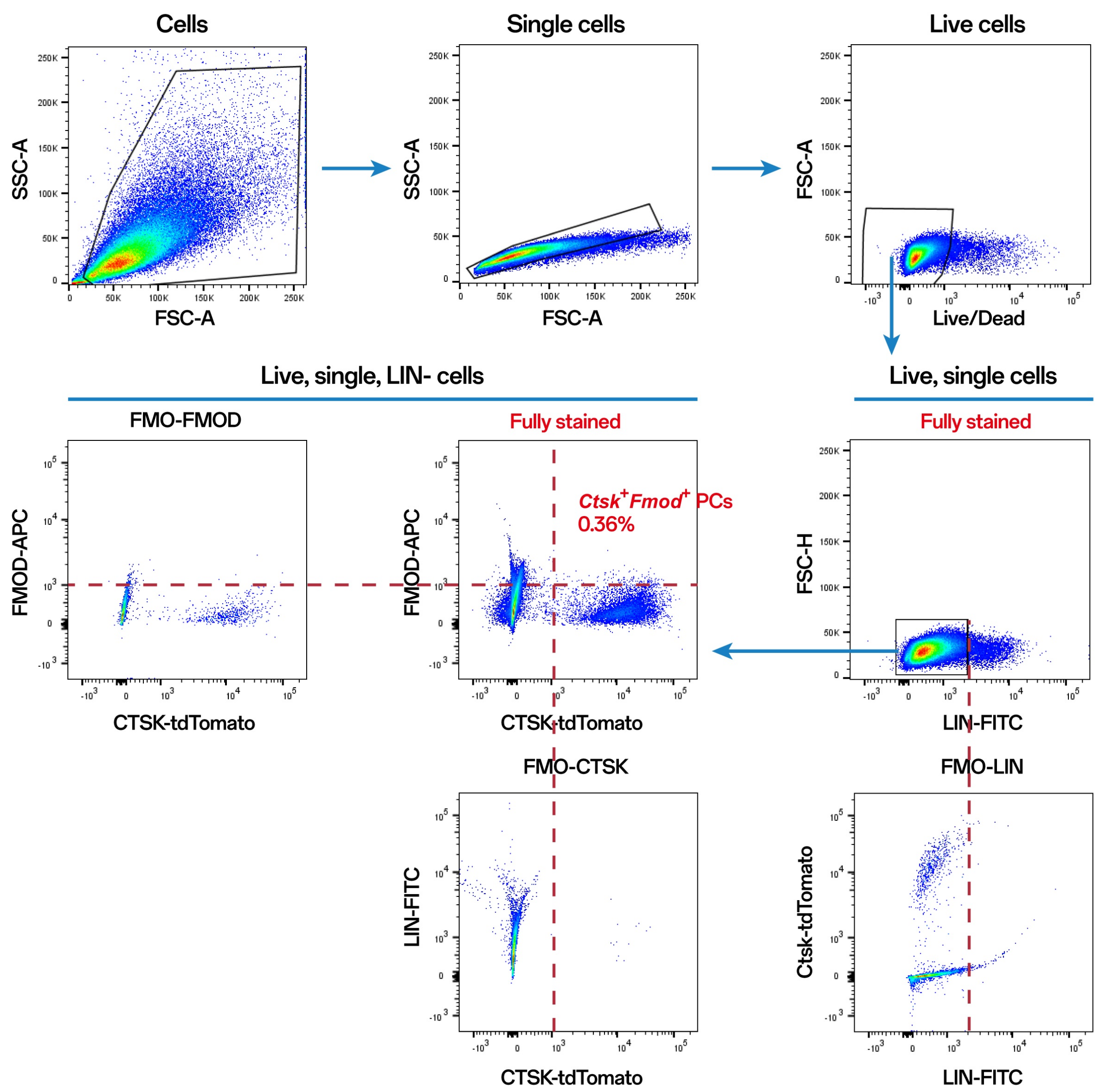

Fig. S9

Gating scheme for *Ctsk+Fmod+* PCs from mice. Multicolor flow cytometry gating strategy to isolate *Ctsk+Fmod+* PCs from single cell suspension. Representative flow cytometry plots of periosteal cells of *Ctsk-cre; Rosa26-tdTomato* mice. FMO-Fluorescence Minus One. Lin-*CD45+CD31+Ter119+*.

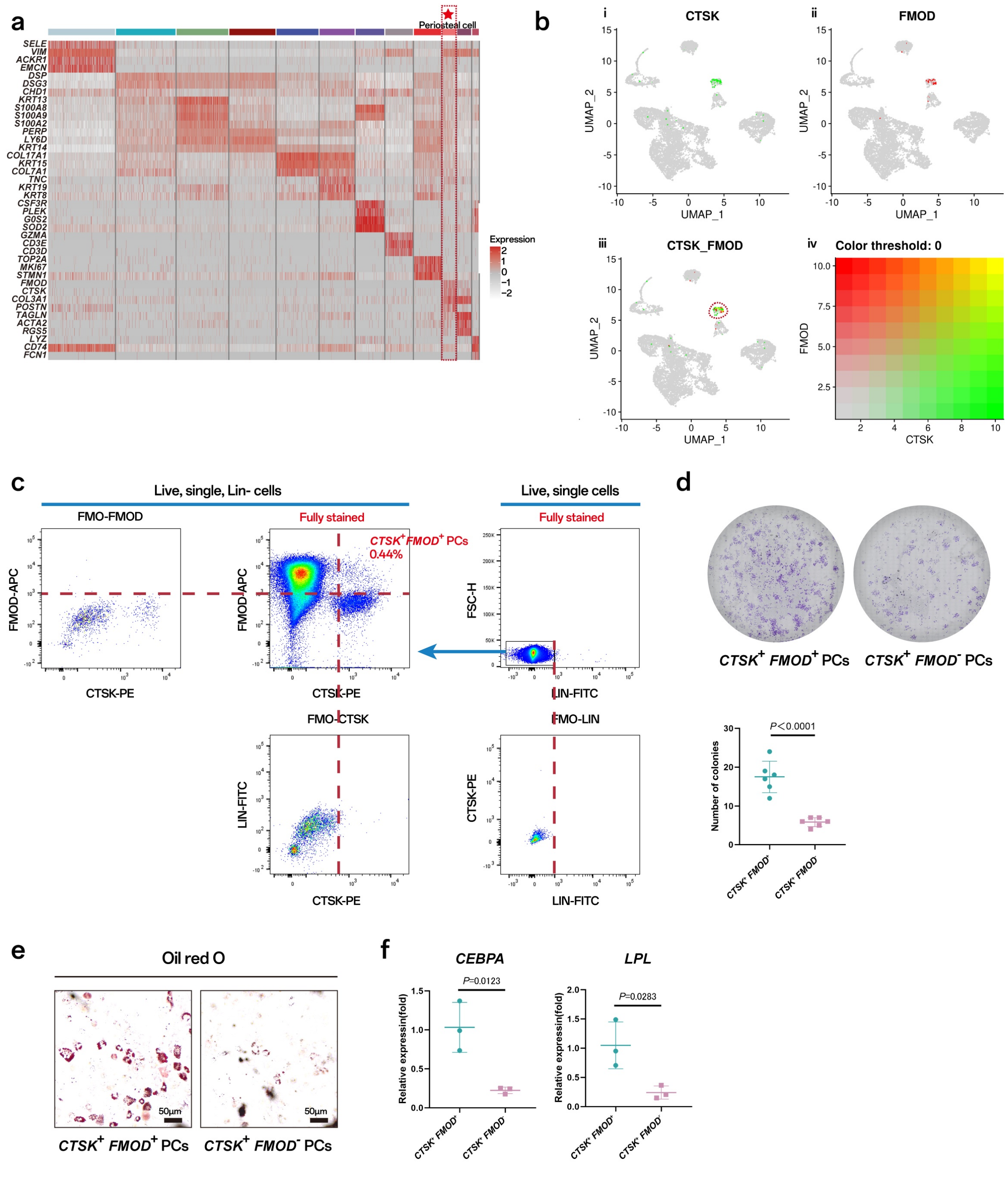

Fig. S10

Characteristics of *CTSK+FMOD+* PCs from human. (a) Heatmap showed top marker genes for each cluster. The stars showed the periosteal cells. **(b)** Blended feature plots showing co-expression of *CTSK* and *FMOD*. (i) The expression of *CTSK*; (ii) The expression of *FMOD*; (iii) The co-expression of *CTSK* and *FMOD*; (iv) The expression level of *FMOD* (red) and *CTSK* (green). These two markers were colocalized in human periosteal cells. Gating scheme for *CTSK+FMOD+* PCs. **(c)** Multicolor flow cytometry gating strategy to isolate *CTSK+FMOD+* PCs from single cell suspension. Representative flow cytometry plots of human. FMO-Fluorescence Minus One. LIN-*CD45+CD31+Ter119+*. **(d)** Colony formation of *CTSK+FMOD+* PCs at Day14 via crystal violet staining. **(e)** Oil red O staining of *CTSK+FMOD+* PCs and *CTSK+FMOD-* PCs on Day21. **(f)** qPCR analysis of the mRNA levels of CEBPA and LPL of *CTSK+FMOD+* PCs and *CTSK+FMOD-* PCs.

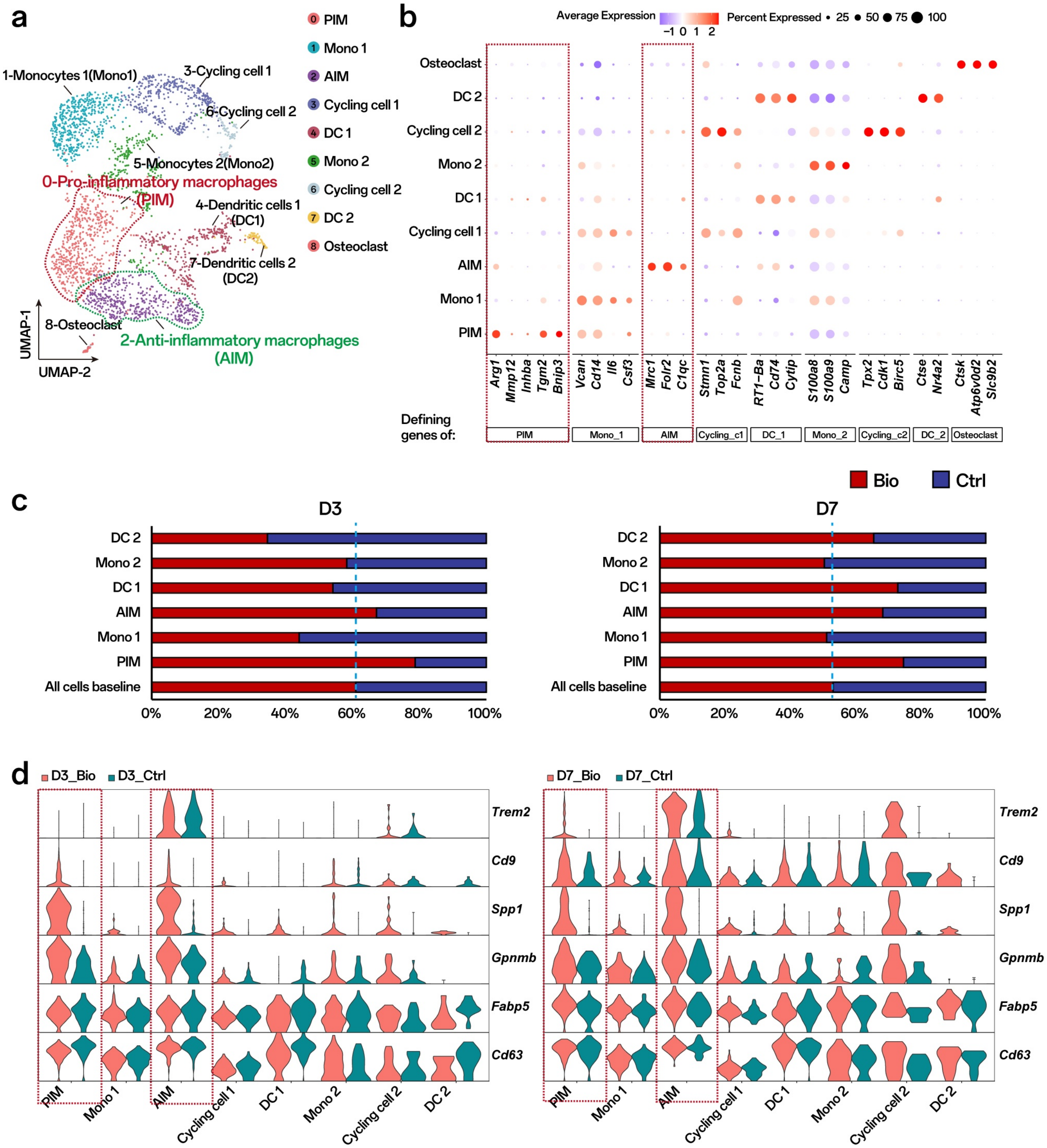
a
PIM
Mono 1
AIM
Cycling cell 1
DC 1
Mono 2
Cycling cell 2
DC 2
Osteoclast
1-Monocytes 1(Mono1)
3-Cycling cell 1
6-Cycling cell 2
5-Monocytes 2(Mono2)
0-Pro-inflammatory macrophages (PIM)
4-Dendritic cells 1 (DC1)
7-Dendritic cells 2 (DC2)
8-Osteoclast
2-Anti-inflammatory macrophages (AIM)
UMAP-1
UMAP-2
b
Average Expression
Percent Expressed
Defining genes of:
PIM
Mono_1
AIM
Cycling_c1
DC_1
Mono_2
Cycling_c2
DC_2
Osteoclast
c
Bio
Ctrl
D3
D7
All cells baseline
d
D3_Bio
D3_Ctrl
D7_Bio
D7_Ctrl
Trem2
Cd9
Spp1
Gpnmb
Fabp5
Cd63

Fig. S11

Subpopulation analysis of macrophages. (a) UMAP plots showing subclustering results of macrophages. The red circle indicates Pro-inflammatory macrophages and the green circle indicates Anti-inflammatory macrophages. PIM-Pro-inflammatory macrophages, Mono-Monocytes, AIM-Anti-inflammatory macrophages, DC-Dendritic cells. **(b)** Dot plots showing expression percentage and average expression of markers in subtypes of macrophages. **(c)** Bar plots showing proportion of macrophages subclusters in different group on Day3 and Day7. **(d)** Violin plots showing expression percentage and average expression of signature of fibroblast-associated markers (*Trem2, Cd9, Spp1, Gpnmb, Fabp5, Cd63*) of macrophages in different groups.

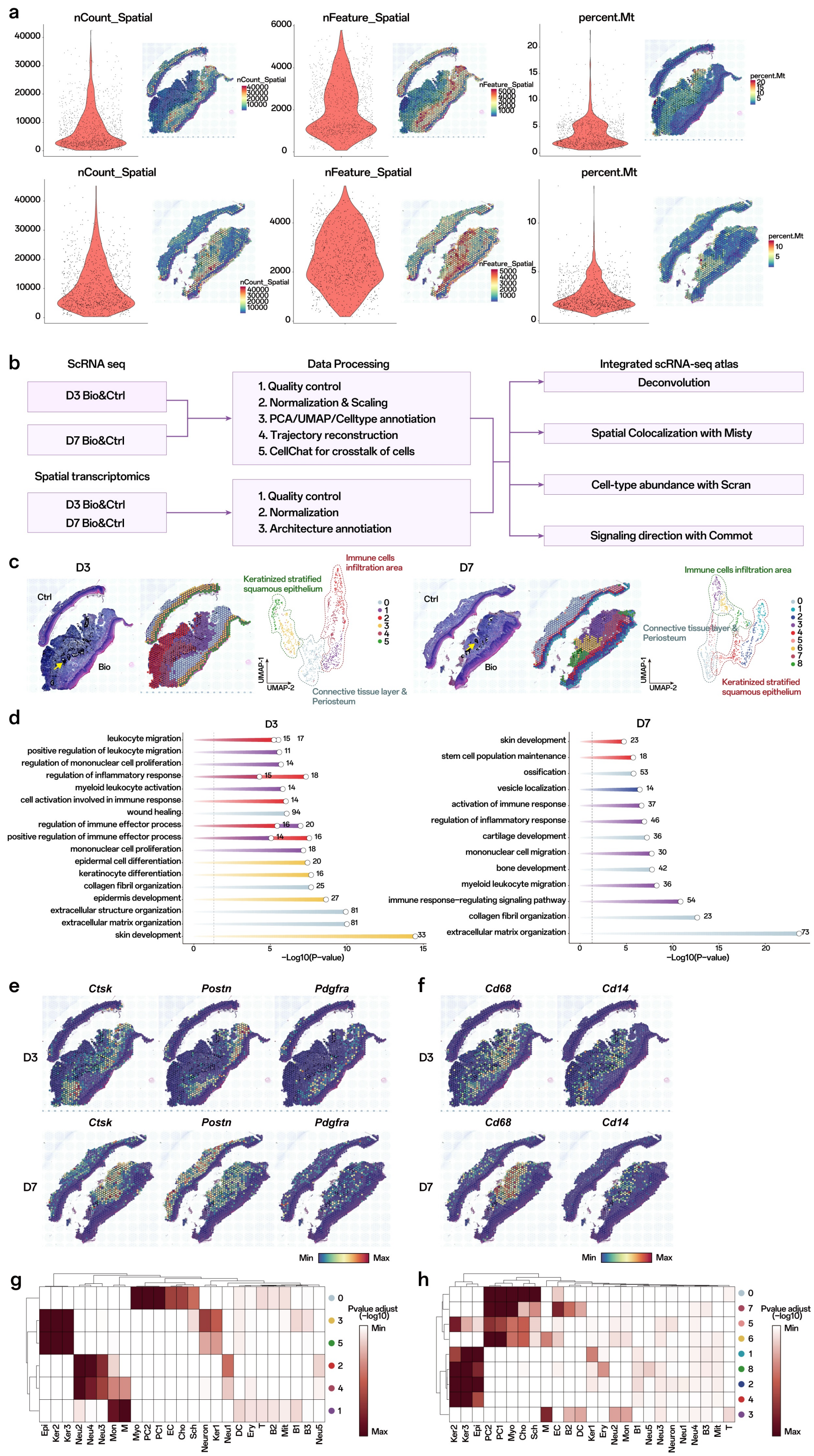

a
nCount_Spatial
nFeature_Spatial
percent.Mt
b
ScRNA seq
D3 Bio&Ctrl
D7 Bio&Ctrl
Data Processing
1. Quality control
2. Normalization & Scaling
3. PCA/UMAP/Celltype annotation
4. Trajectory reconstruction
5. CellChat for crosstalk of cells
Spatial transcriptomics
D3 Bio&Ctrl
D7 Bio&Ctrl
1. Quality control
2. Normalization
3. Architecture annotation
Integrated scRNA-seq atlas
Deconvolution
Spatial Colocalization with Misty
Cell-type abundance with Scran
Signaling direction with Commot
c
D3
Ctrl
Bio
Keratinized stratified squamous epithelium
Immune cells infiltration area
Connective tissue layer & Periosteum
UMAP-1
UMAP-2
D7
Immune cells infiltration area
Connective tissue layer & Periosteum
Keratinized stratified squamous epithelium
d
D3
leukocyte migration
positive regulation of leukocyte migration
regulation of mononuclear cell proliferation
regulation of inflammatory response
myeloid leukocyte activation
cell activation involved in immune response
wound healing
regulation of immune effector process
positive regulation of immune effector process
mononuclear cell proliferation
epidermal cell differentiation
keratinocyte differentiation
collagen fibril organization
epidermis development
extracellular structure organization
extracellular matrix organization
skin development
−Log10(P−value)
D7
skin development
stem cell population maintenance
ossification
vesicle localization
activation of immune response
regulation of inflammatory response
cartilage development
mononuclear cell migration
bone development
myeloid leukocyte migration
immune response−regulating signaling pathway
collagen fibril organization
extracellular matrix organization
−Log10(P−value)
e
Ctsk
Postn
Pdgfra
D3
D7
f
Cd68
Cd14
D3
D7
Min
Max
g
Pvalue adjust (−log10)
Min
Max
Epi
Ker2
Ker3
Neu2
Neu4
Neu3
Mon
M
Myo
PC2
PC1
EC
Cho
Sch
Neuron
Ker1
Neu1
DC
Ery
T
B2
Mit
B1
B3
Neu5
h
Pvalue adjust (−log10)
Min
Max
Ker2
Ker3
Epi
PC2
PC1
Myo
Cho
Sch
M
EC
B2
DC
Ker1
Ery
Neu2
Mon
B1
Neu5
Neu3
Neuron
Neu1
Neu4
B3
Mit
T

Fig. S12

Spatial transcriptomics applied to Bio and Ctrl group. (a) Violin plots of UMI counts, gene features and Mt percent per spot of different timepoints. **(b)** Workflow of scRNA-seq and ST data. **(c)** HE staining of tissue sections (Stars: bone graft substitute) (left) and unbiased clustering of ST spots (middle and right). **(d)** GO analysis of DEGs in Spatial clusters. Spatial feature plot highlighted the expression of marker genes for periosteum cell **(e)** and macrophage **(f)**. MIA enrichment heatmaps of major cell types in the scRNA-seq dataset on Day3 **(g)** and Day7 **(h)**.

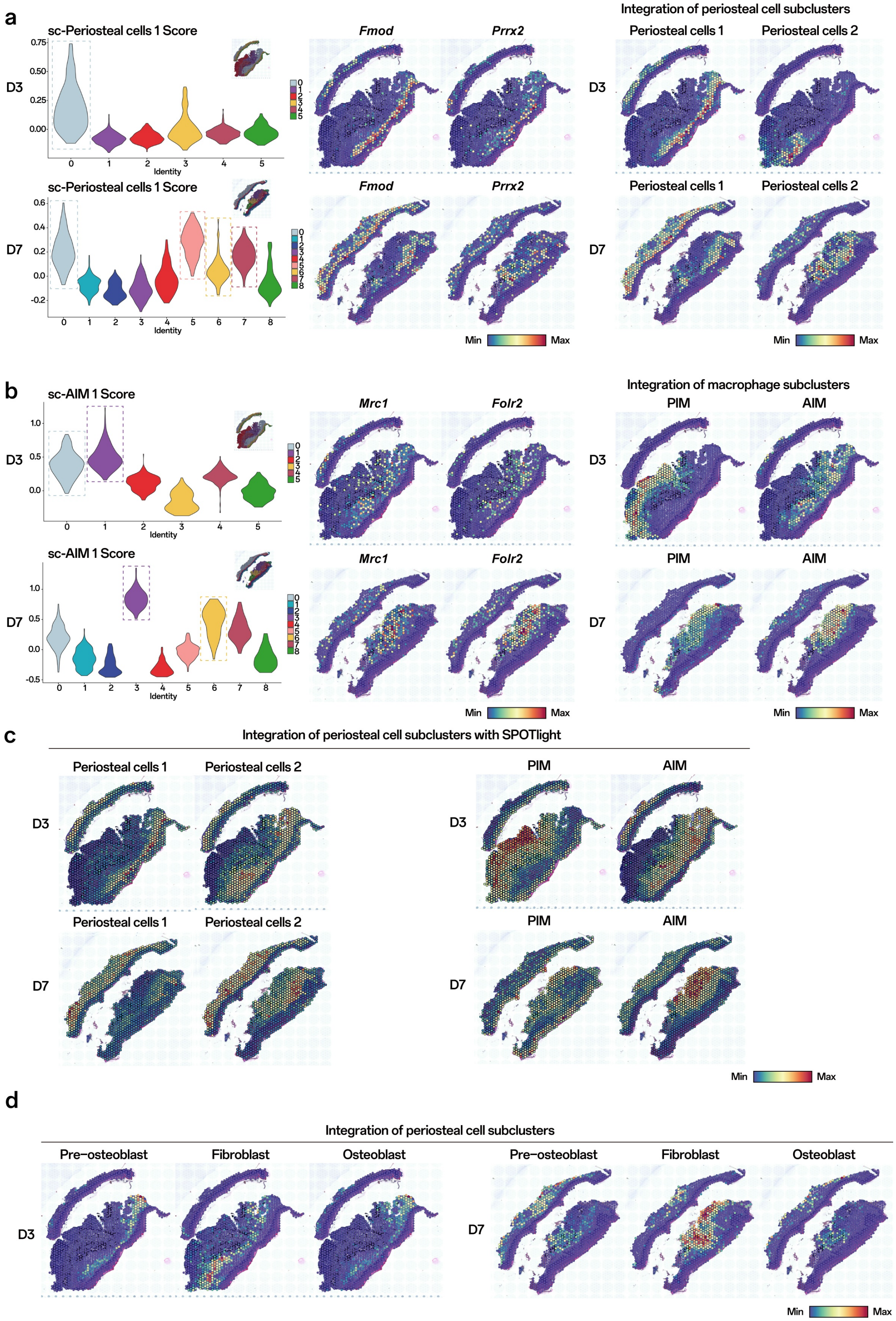

a
Integration of periosteal cell subclusters
sc-Periosteal cells 1 Score
Fmod
Prrx2
Periosteal cells 1
Periosteal cells 2
D3
D7
Identity
Min
Max
b
Integration of macrophage subclusters
sc-AIM 1 Score
Mrc1
Folr2
PIM
AIM
c
Integration of periosteal cell subclusters with SPOTlight
d
Pre−osteoblast
Fibroblast
Osteoblast

Fig. S13

Colocalization of PCs and macrophage subclusters revealed by spatial transcriptomics. (a) Violin plots showing scores of periosteal cell 1 (*Ctsk*+*Fmod*+ PCs) genes of individual spots derived from scRNA-seq for clusters of ST and its marker expression in tissue sections at Day3(up) and Day7(down). Spatial feature plots(right) showing defined subtypes of PCs distribution in tissue sections at Day3 and Day7. **(b)** Violin plots showing scores of AIM genes of individual spots derived from scRNA-seq for clusters of ST and its marker expression in tissue sections at Day3(up) and Day7(down). Spatial feature plots (right) showing defined subtypes of macrophages distribution in tissue sections at Day3 and Day7. **(c)** Predicted proportion within each capture spot for periosteal cell 1 (*Ctsk*+*Fmod*+ PCs), periosteal cell 2 as well as PIM and AIM with SPOTlight. **(d)** Spatial feature plots showing defined subtypes of PCs distribution in tissue sections at Day3 and Day7.

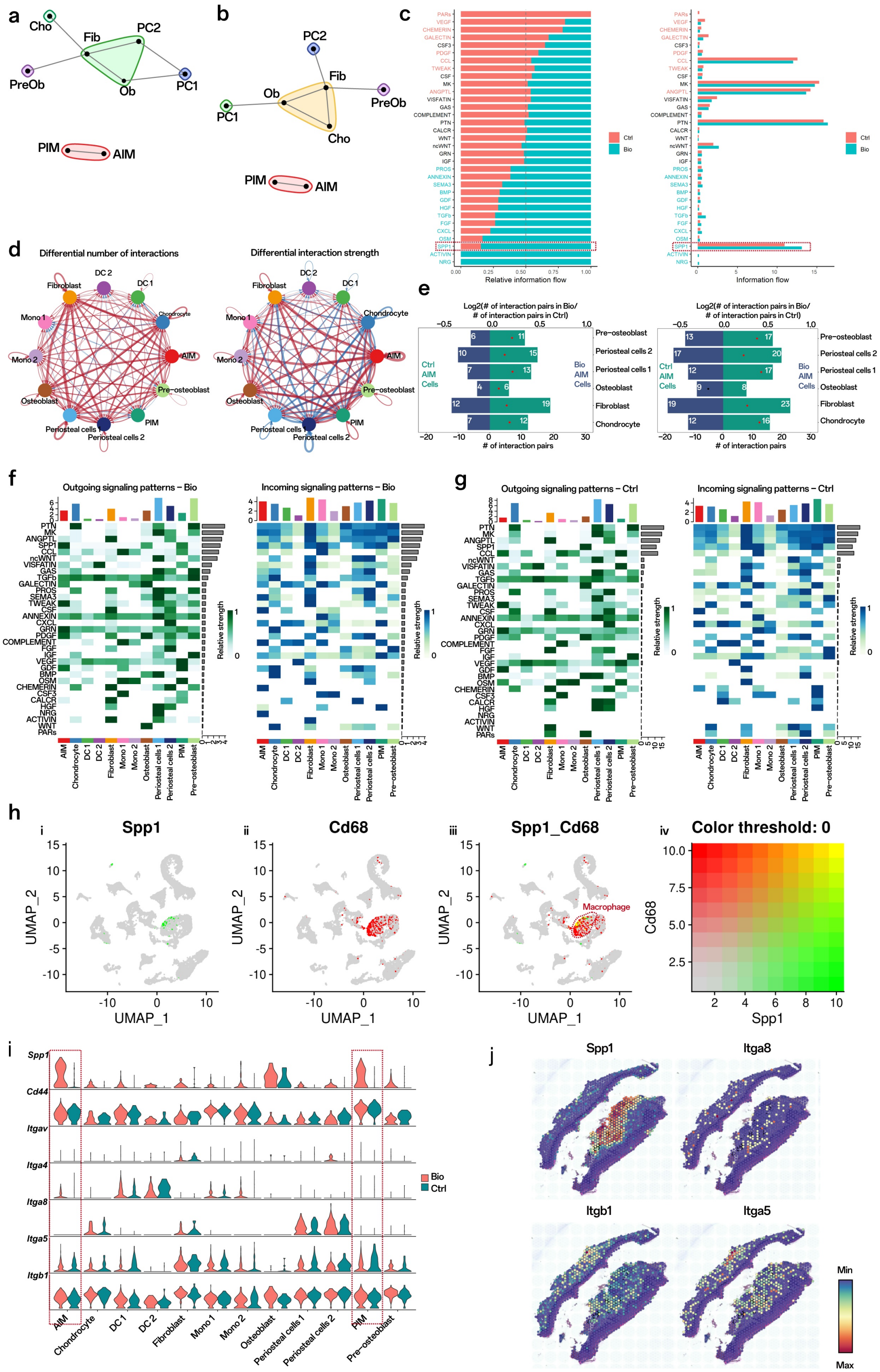
a
b
c
d
Differential number of interactions
Differential interaction strength
e
f
Outgoing signaling patterns – Bio
Incoming signaling patterns – Bio
g
Outgoing signaling patterns – Ctrl
Incoming signaling patterns – Ctrl
h
Spp1
Cd68
Spp1_Cd68
Color threshold: 0
Macrophage
i
j
Spp1
Itga8
Itgb1
Itga5

Fig. S14

Cellular communication landscape between PCs and macrophages. Intra-view cell-cell interaction networks using Misty of Day3 **(a)** and Day7 **(b)**. **(c)** Comparison of the information flow between Bio and Ctrl group. **(d)** Net plot showing the interaction number and strength between each cluster. **(e)** Bar chart showing the number of significant ligand-receptor pairs in PIM (left) and AIM (right) with periosteal cells subclusters in different groups. The dots represent the ratio of the number of interaction pairs between Bio and Ctrl groups and the ratio above 1 in red and below 1 in black. Heatmap with the relative strength of each signaling pathways for clusters of PCs and macrophages in Bio group **(f)** and Ctrl group **(g)**. **(h)** Blended feature plots showing co-expression of Spp1 and Cd68. (i) The expression of Spp1; (ii) The expression of Cd68; (iii) The co-expression of Spp1 and Cd68; (iv) The expression level of Spp1 (red) and Cd68 (green). These two markers were colocalized in macrophages (red circle). **(i)**Violin plots showing the ligand-receptors analysis of SPP1 signaling in Bio and Ctrl group. **(j)** ST showed select ligands and receptors expression of SPP1 signaling.

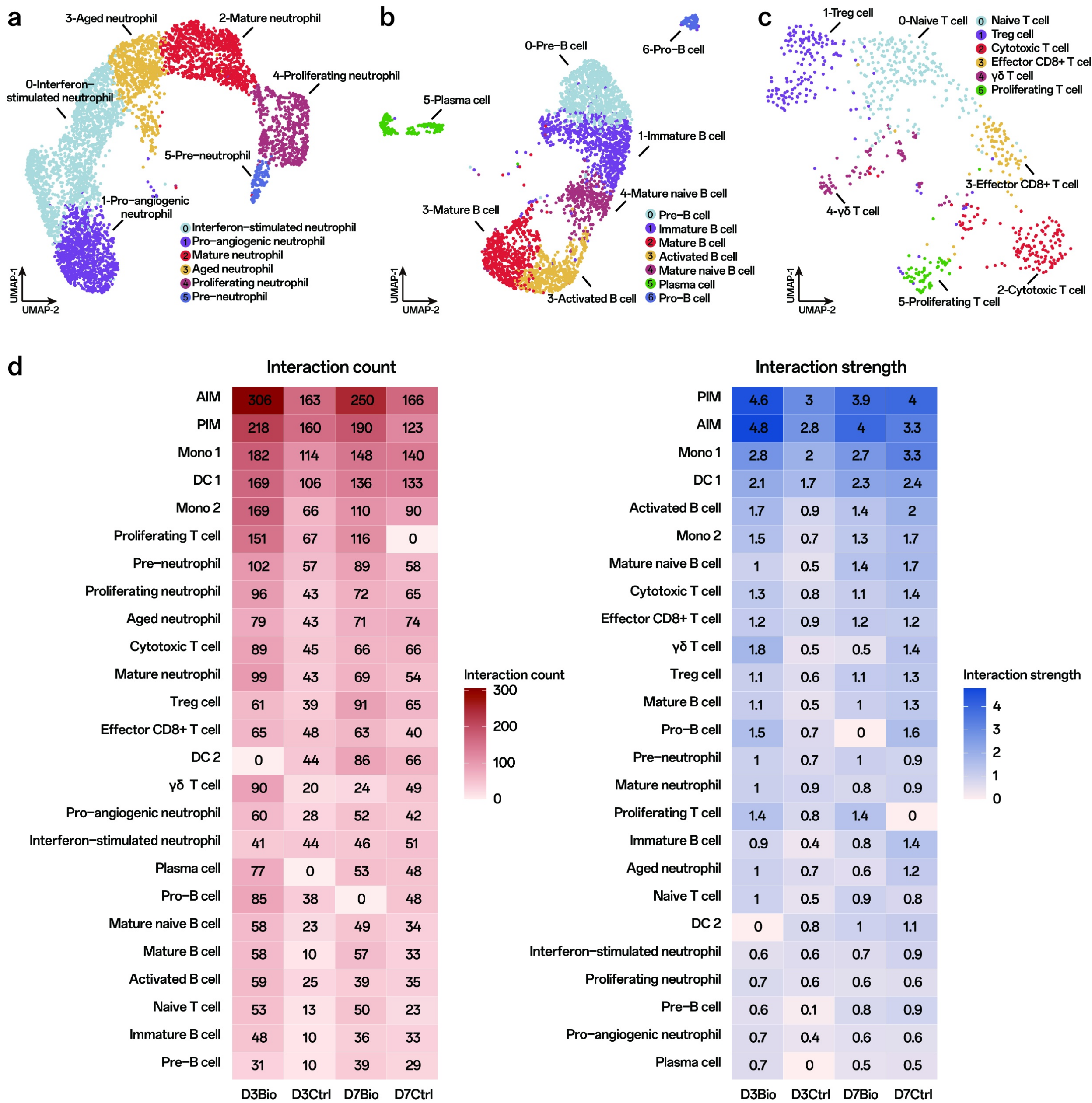

Fig. S15

Cellular communication landscape between PCs and immune cells. UMAP plots showing subclustering results of neutrophils **(a)**, B cells **(b)** and T cells **(c)**. **(d)** The interaction count and interaction strength between PCs and immune cells across different groups.

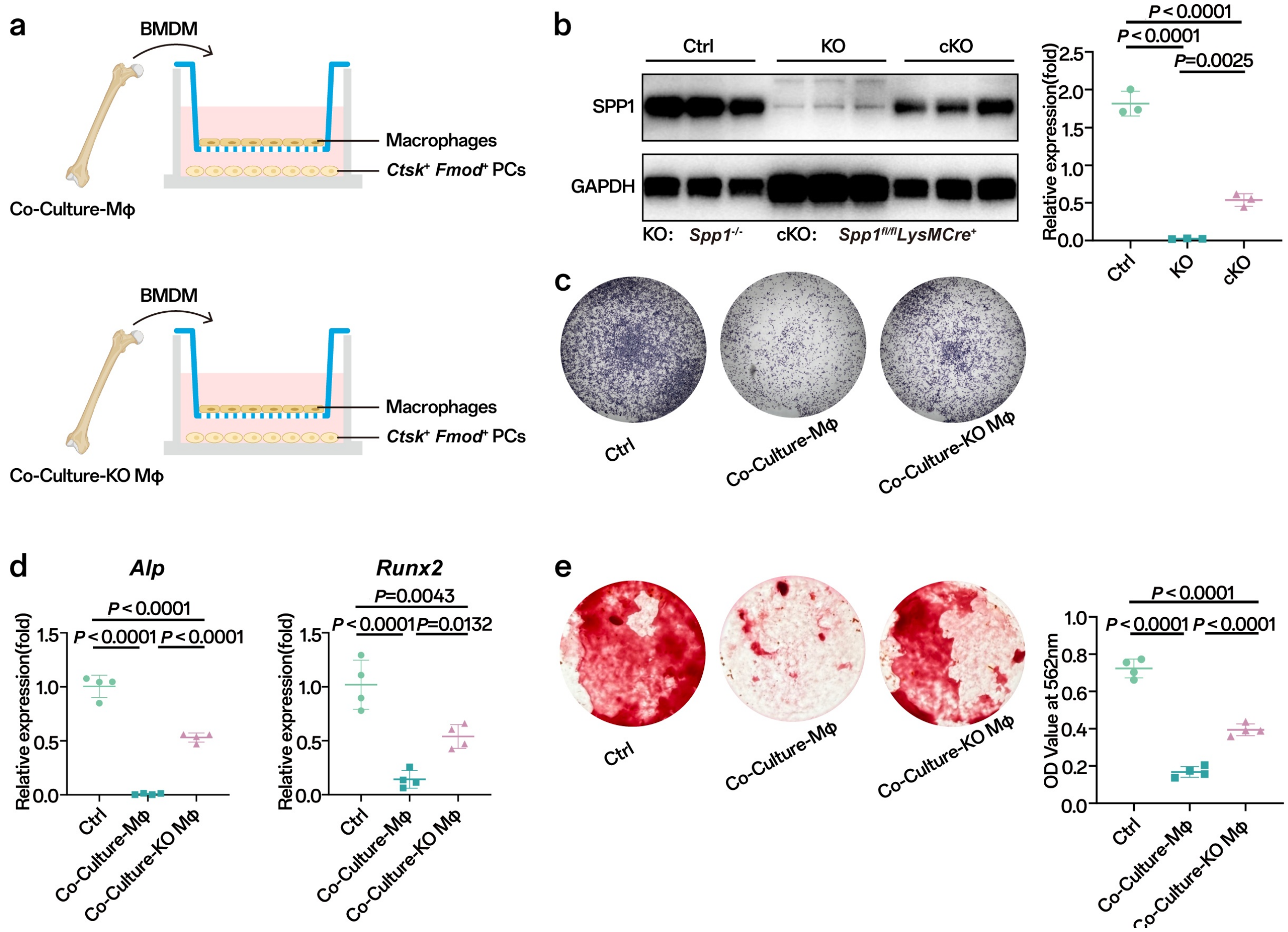

Fig. S16.

Macrophage-derived SPP1 reduced the osteogenic capacity of *Ctsk+Fmod+* PCs. (a) Schematic representation of the co-culture system of macrophages (from WT and SPP1 KO mice) and *Ctsk+Fmod+* PCs. **(b)** Western blot analysis of SPP1 expressed in macrophage from WT, KO and conditional knockout(cKO). GAPDH was used as a reference protein and corresponding quantification of the SPP1 level. **(c)** ALP staining of PCs in different group on Day7. **(d)** qPCR analysis of the mRNA levels of *Alp* and *Runx2* of *Ctsk+Fmod+* PCs in different groups. **(e)** ARS staining and semiquantitative analysis of PCs in different groups on Day14.

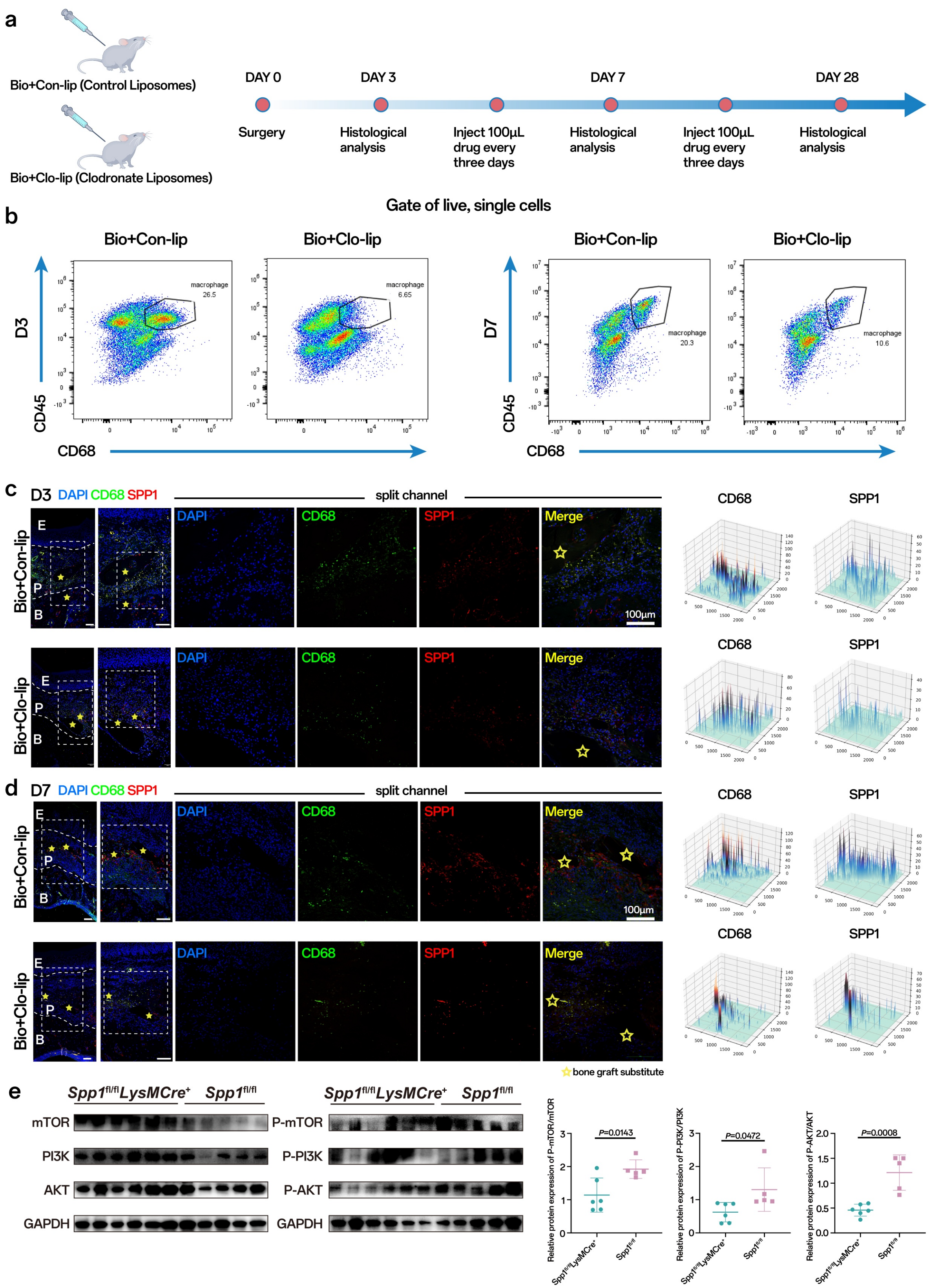

a
Bio+Con-lip (Control Liposomes)
Bio+Clo-lip (Clodronate Liposomes)
DAY 0
DAY 3
DAY 7
DAY 28
Surgery
Histological analysis
Inject 100μL drug every three days
Histological analysis
Inject 100μL drug every three days
Histological analysis
b
Gate of live, single cells
Bio+Con-lip
Bio+Clo-lip
Bio+Con-lip
Bio+Clo-lip
D3
D7
CD45
CD68
macrophage 26.5
macrophage 6.65
macrophage 20.3
macrophage 10.6
c
D3 DAPI CD68 SPP1
split channel
Bio+Con-lip
Bio+Clo-lip
E
P
B
DAPI
CD68
SPP1
Merge
100μm
d
D7 DAPI CD68 SPP1
split channel
Bio+Con-lip
Bio+Clo-lip
bone graft substitute
e
Spp1fl/flLysMCre+
Spp1fl/fl
mTOR
PI3K
AKT
GAPDH
P-mTOR
P-PI3K
P-AKT
Relative protein expression of P-mTOR/mTOR
Relative protein expression of P-PI3K/PI3K
Relative protein expression of P-AKT/AKT
P=0.0143
P=0.0472
P=0.0008

Fig. S17.

Macrophage depletion at the surgical site and the dectetion of PI3K/AKT/mTOR pathway of cKO and Ctrl mice. (a) Workflow of macrophages depletion *in vivo*. **(b)** *In vivo* administration of clodronate liposome depleted macrophages. Immunofluorescence staining of CD68 and SPP1 in operation areas at Day3 **(c)** and Day7 **(d)**, and normalized fluorescence intensity at Day3 and Day7. Stars, bone graft substitute. Abbreviations: E, epithelium; P, periosteum; B, bone. Scale bar, 100µm. **(e)** Western blot analysis of protein of PI3K/AKT/mTOR pathway expressed in macrophage from cKO and Ctrl mice. GAPDH was used as a reference protein and corresponding quantification of the protein level of PI3K/AKT/mTOR pathway.

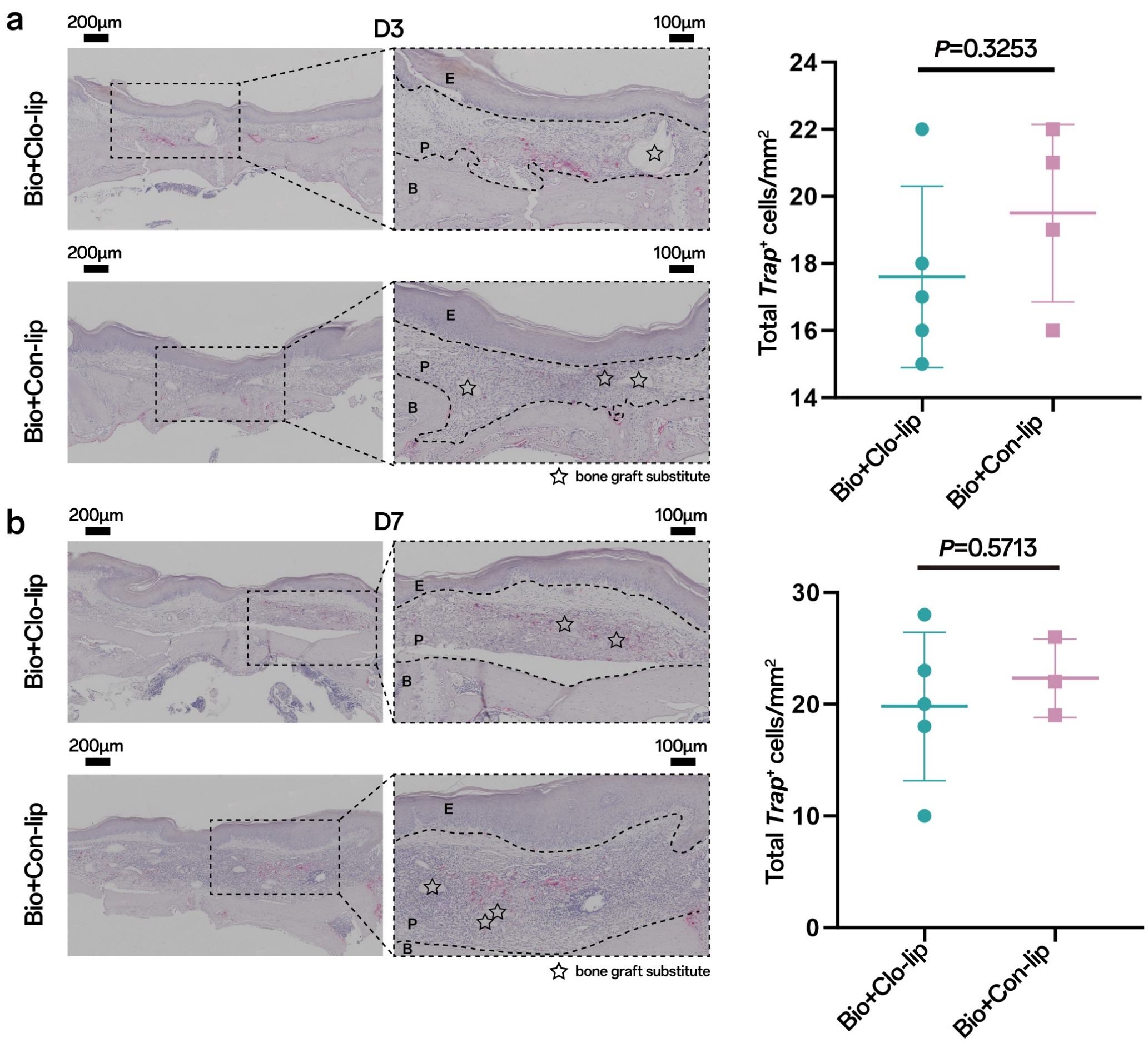

Fig. S18.

Trap Staining of different group. Representative images of TRAP staining of the two groups on Day3 **(a)**, Day7 **(b)** and the quantitative analysis of the TRAP staining. Stars, bone graft. Abbreviations: E, epithelium; P, periosteum; B, bone.

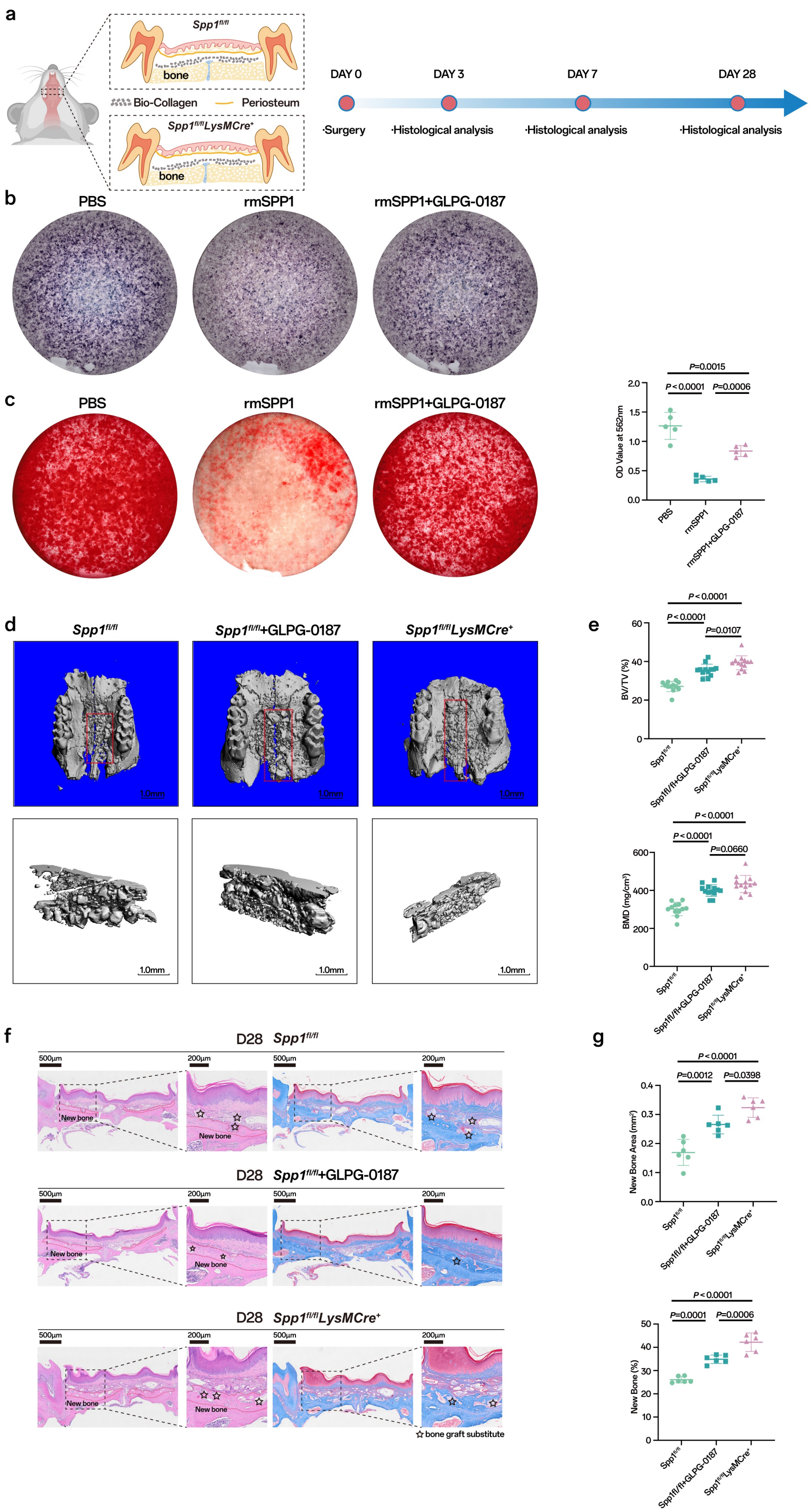
a
Spp1fl/fl
bone
Bio-Collagen
Periosteum
Spp1fl/flLysMCre+
bone
DAY 0
DAY 3
DAY 7
DAY 28
·Surgery
·Histological analysis
·Histological analysis
·Histological analysis
b
PBS
rmSPP1
rmSPP1+GLPG-0187
c
PBS
rmSPP1
rmSPP1+GLPG-0187
OD Value at 562nm
P=0.0015
P<0.0001
P=0.0006
d
Spp1fl/fl
Spp1fl/fl+GLPG-0187
Spp1fl/flLysMCre+
1.0mm
e
BV/TV (%)
P<0.0001
P<0.0001
P=0.0107
BMD (mg/cm³)
P<0.0001
P<0.0001
P=0.0660
f
D28 Spp1fl/fl
D28 Spp1fl/fl+GLPG-0187
D28 Spp1fl/flLysMCre+
500μm
200μm
New bone
bone graft substitute
g
New Bone Area (mm²)
P<0.0001
P=0.0012
P=0.0398
New Bone (%)
P<0.0001
P=0.0001
P=0.0006

Fig. S19.

Inhibition of integrin with GLPG-0187 improved the osteogenic capacity of *Ctsk+Fmod+* PCs. (a) Workflow of mice subperiosteal bone grafting model establishment and sample analysis. **(b)** ALP staining of PCs in different group on Day7. **(c)** ARS staining and semiquantitative analysis of PCs in different groups on Day14. **(d)** Representative images of micro-CT reconstruction volume of ROI in different groups. **(e)** Quantitative micro-CT analyses of BV/TV (%) and BMD (mg/cm3). **(f)** Histological staining of H&E; and Masson after 28 days in different groups. Stars, bone graft substitute. Red dotted line, new bone area. **(g)** Semiquantitative analysis of new bone area and the percentage of new bone.

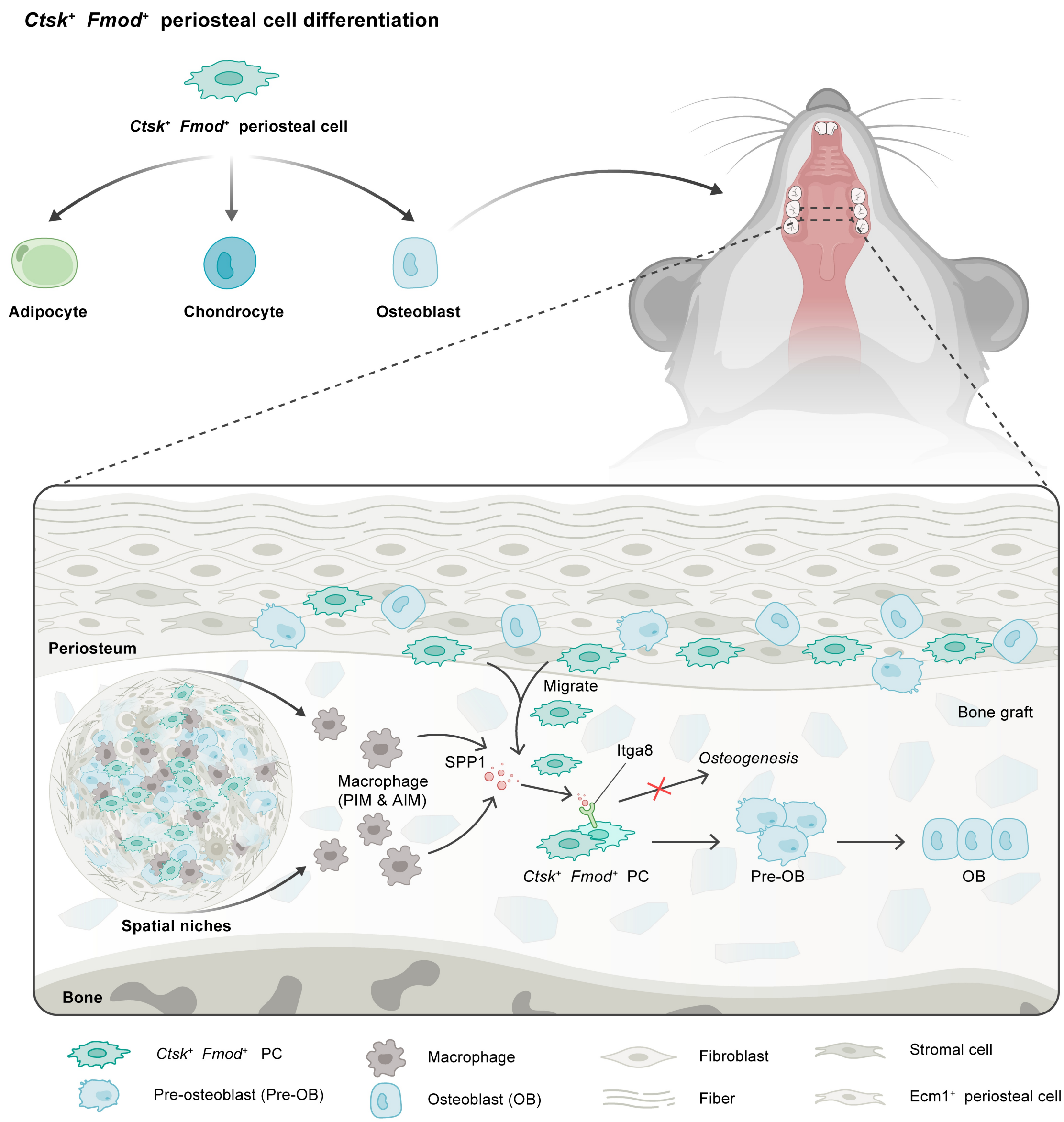

Fig. S20.

Schematic of jaw-bone periosteal spatial architecture. The *Ctsk+Fmod+* periosteal cells (PCs) of jaw bones have osteoprogenitor potential. After biomaterial grafting, *Ctsk+Fmod+* PCs were activated. And macrophage subpopulations and periosteal cell subpopulations establish intricate ecological niches surrounding the bone graft substitute. SPP1 secreted by recruited macrophages could promote fibrosis and impair the osteogenic capacity of *Ctsk+Fmod+* PCs.
